# Microbiome-host communication via vaccenic acid modulates LRH-1/NR5A2 activity and ameliorates metabolic liver disease

**DOI:** 10.1038/s44321-026-00516-3

**Published:** 2026-09-17

**Authors:** Anna Pia Plazzo, Verena Filz, Michaela Prothiwa, Rebekka Lambrecht, M Eugenia Delgado, Jennifer R Fleming, Jerome Duschek, Elia Pilgram, Juliane Friedrich, Raphael Eisenring, Lea Kurtz, Kerstin Stemmer, Lotta K Stenman, Fabian Amaya Garcia, Miriam Unterlass, Olga Mayans, Thomas Böttcher, Thomas Brunner

**Affiliations:** 1 Biochemical Pharmacology, Department of Biology, University of Konstanz, Konstanz, Germany; 2 Department of Microbiology and Ecosystem Science and Department of Biological Chemistry, University of Vienna, Vienna, Austria; 3 Department of Systems Immunology, Weizmann Institute of Science, Rehovot, Israel; 4 Department of Pharmacology, Toxicology and Therapeutic Chemistry, Faculty of Pharmacy and Food Science, University of Barcelona, Barcelona, Spain; 5 Biophysics and Structural Biology, Department of Biology, University of Konstanz, Konstanz, Germany; 6 Division of Molecular Cell Biology, Institute of Theoretical Medicine, Faculty of Medicine, University of Augsburg, Augsburg, Germany; 7 DuPont Nutrition & Biosciences, Kantvik, Finland; 8 Fraunhofer Institute for Silicate and University of Würzburg, Würzburg, Germany

**Keywords:** Digestive System, Metabolism, Microbiology, Virology & Host Pathogen Interaction

## Abstract

Interactions between microbiota and the host can profoundly affect human health. In particular, microbiota-derived metabolites influence liver physiology and contribute to the development of metabolic diseases, yet the molecular mechanisms underlying microbiome-host communication remain incompletely understood. Here, we identify bacterial lipids as modulators of the nuclear receptor Liver Receptor Homolog-1 (LRH-1/NR5A2), a regulator of hepatic metabolism. Lipid extracts from multiple bacterial species, including the probiotic *Bifidobacterium animalis* subsp. *lactis* B420^TM^, activated LRH-1, leading to the identification of the long-chain fatty acid vaccenic acid (VA) as a previously unrecognized endogenous LRH-1 ligand. VA directly bound the LRH-1 ligand-binding domain and stimulated receptor-dependent transcriptional activity. In high-fat diet-induced obese and hyperglycemic mice, VA-mediated LRH-1 activation improved glucose homeostasis and attenuated metabolic liver disease. Transcriptomic analyses revealed suppression of hepatic de novo lipogenesis as a principle downstream response to LRH-1 activation. Together, these findings establish a microbiota-LRH-1 signaling axis that links bacterial lipid metabolism to host metabolic regulation, identify VA as a functional LRH-1 agonist, and provide a mechanistic rationale for targeting LRH-1 in metabolic liver disease.

## Synopsis

Microbiota-host nuclear receptor communication is increasingly recognized to regulate human health and disease. This study describes vaccenic acid, isolated from gut bacteria, as a novel LRH-1 ligand and activator, capable of improving metabolic disease development when administered in vivo.

- LRH-1 is a nuclear receptor, regulating metabolic processes.
- Lipids from gut bacteria were found to regulate activity of LRH-1.
- Vaccenic acid (VA), a natural fatty acid found mainly in meat and dairy products, was identified from these lipids as a novel LRH-1 activator.
- In a mouse model of diet-induced metabolic syndrome, VA improved glucose homeostasis and reduced hepatic lipogenesis.
- These findings reveal a new microbiota-liver communication pathway through VA-mediated LRH-1 activation, and suggest potential therapeutic approaches employing diet, probiotics, prebiotics, or VA-based therapies.

## Abstract (web summary)

Microbiota-host nuclear receptor communication is increasingly recognized to regulate human health and disease. This study describes vaccenic acid, isolated from gut bacteria, as a novel LRH-1 ligand and activator, capable of improving metabolic disease development when administered in vivo.

## Graphical abstract

**Figure:**
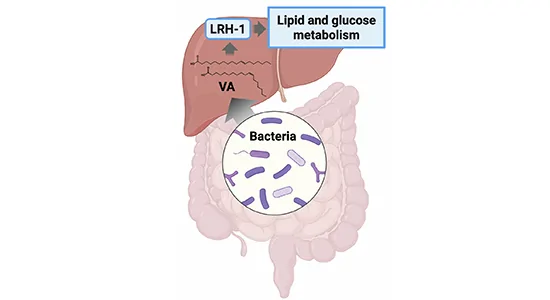

The paper explained **Problem**

Our body hosts trillions of microbes, especially in the gut, collectively known as the microbiota. These microorganisms can produce small molecules that can travel through the body and influence the health of different organs, including the liver. Communication with the host occurs in part via binding to certain proteins, called nuclear receptors. As disturbances in the equilibrium of such populations of microorganisms has been linked to several diseases, such as obesity, type 2 diabetes and fatty liver disease, understanding the mechanisms how bacteria-derived molecules can activate nuclear receptors is fundamental to develop new therapeutic opportunities.

**Results**

In this study, we investigated how gut bacteria communicate with the liver through the nuclear receptor LRH-1, which is a protein that plays a central role in controlling blood sugar levels, fat metabolism, and liver health, among others. We discovered that lipids isolated from certain commensal and probiotic bacteria can regulate LRH-1 activity. Most importantly, we identified vaccenic acid (VA), a natural lipid molecule mostly found in meat and dairy products, as a new activator of LRH-1.

To test the medical relevance of this finding, we studied obese mice with high blood sugar caused by a high-fat diet. Activating LRH-1 with VA in these mice significantly improved glucose control and reduced liver disease. At the molecular level, VA decreased production of new fats by the liver, a process that contributes to fatty liver disease and insulin resistance.

**Impact**

These findings reveal a new microbiota-liver communication pathway, where molecules produced by health-promoting bacteria help to regulate host metabolism through LRH-1 activation. Clinically, this opens the possibility of treating metabolic disorders, linked to impaired LRH-1 function, using targeted diets, probiotics, prebiotics, or VA-based therapies.

## Introduction

Host-microbiota interactions are being increasingly recognized to critically regulate various physiological and pathological processes in the host. Dysbiosis, the disturbance of the equilibrium of the microbiome, has been linked to several pathological conditions, such as gastrointestinal and hepatic inflammatory disorders, metabolic and cardiovascular diseases, autoimmune reactions and allergies (Hou et al, 2022; Fan and Pedersen, 2021; Gomaa, 2020). Bacteria populating the gastrointestinal tract represent the vast majority of the human microbiome. These gut bacteria contribute significantly to intestinal health by maintaining epithelial homeostasis and supporting the integrity of the intestinal barrier, as described for the probiotic *Lactobacillus*, *Bifidobacterium*, *Streptococcus* and some *Escherichia coli* strains (Eckburg et al, 2005; King et al, 2019). In addition, microbial metabolites and secretory factors can enter the portal circulation and shape the health status and lipid storage profile of the liver.

Bacterial metabolites interact with host receptors, including nuclear receptors (NRs), to regulate transcriptional programs and enzymatic functions in target tissues. Disruption of these microbiota-host interactions is the underlying cause of several pathological conditions, such as inflammatory bowel disease (IBD), hepatic steatosis and type-2 diabetes (Ranhotra, 2017; Wang et al, 2021).

NRs belong to a superfamily of ligand-activated transcription factors with major roles in the regulation of a wide variety of physiological processes. NR ligands are, in general, small lipophilic natural or synthetic compounds. One of the first discovered examples of bacteria-host NR communication is represented by the Farnesoid-X-Receptor (FXR, NR1H4) and its cognate ligands bile acids (BA). Primary BA are synthesized from cholesterol by hepatocytes in a series of enzymatic steps that include the two key enzymes cholesterol 7α-hydroxylase (CYP7A1) and sterol 12α-hydroxylase (CYP8B1). These BA act as FXR ligands, which regulate their own synthesis and transport via a well-established feedback mechanism (Goodwin et al, 2000; Lu et al, 2000). Hepatic, ligand-stimulated FXR induces the expression of Small Heterodimer Partner (SHP, NR0B2), which in turn inhibits Liver Receptor Homolog-1 (LRH-1, NR5A2)-mediated expression of *CYP7A1* and *CYP8B1* (Lee and Moore, 2002). In addition, FXR-regulated expression of Fibroblast Growth Factor FGF15/19 further represses BA synthesis in the liver by inhibiting CYP7A1 (Inagaki et al, 2005). Primary BA are biotransformed in the intestinal tract by the resident bacteria into secondary BA with different affinities for FXR. Consequently, gut bacteria populations can differentially regulate BA synthesis and efficacy of intestinal lipid absorption (Sayin et al, 2013; Li et al, 2013). Thus, nutrients affecting microbiota composition can also indirectly regulate NR-regulated host processes. For example, theabrownin, a pigment in Pu-erh tea, modulates the gut microbiome and thereby regulates BA homeostasis via FXR/FGF15 signaling (Huang et al, 2019).

LRH-1 is a NR primarily expressed in tissues of endodermal origin, including the liver, intestine, pancreas and ovaries (Bookout et al, 2006), where it regulates embryonic development, intestinal glucocorticoid (GC) synthesis and inflammation, cell proliferation and metabolic processes (Michalek and Brunner, 2021). Dysfunction and/or overexpression of LRH-1 is associated with pathological conditions, including type-2 diabetes, non-alcoholic fatty liver disease (NAFLD, also referred to as metabolic-associated fatty liver disease, MAFLD), IBD and cancer (Antwi et al, 2024; Michalek and Brunner, 2021; Sun et al, 2021; Wu et al, 2024). For this reason, pharmacological targeting of this receptor with agonistic and antagonistic ligands represents a promising therapeutic strategy (Lang et al, 2021). LRH-1 is still considered an orphan nuclear receptor, as thus far no bona fide endogenous ligand has been identified. Yet, current research efforts identified novel natural and synthetic LRH-1 ligands and led to the development of synthetic LRH-1 modulators (recently reviewed in (Lang et al, 2021; Wu et al, 2024). Various of these ligands have already been tested in preclinical mouse models for their activities to modulate LRH-1-regulated physiological and pathophysiological processes. For example, administration of the LRH-1 agonist 1,2-dilauroyl-*sn*-glycero-3-phosphocholine (DLPC) improved serum glucose and insulin sensitivity, and mitigated hepatic lipid accumulation in mouse models of insulin resistance (Lee et al, 2011). Additionally, “phospholipid mimics”, synthetic molecules that combine the phospholipid interactions with the mouth of LRH-1 ligand-binding pocket (LBP) with the deep pocket interactions of the synthetic ligand RJW100, have been shown to reduce lipogenic gene expression in the liver of mice overexpressing human LRH-1 (Cato et al, 2022). We have previously demonstrated that in the intestine LRH-1 regulates steroidogenic enzyme expression, thereby controlling extra-adrenal GC synthesis (Mueller et al, 2006; Coste et al, 2007; Ahmed et al, 2023; Noti et al, 2010). Accordingly, synthetic LRH-1 ligands have been found to promote intestinal steroidogenesis and lower hepatic lipogenic genes such as *Fasn*, *Scd1* and *Acacb* (Mays et al, 2022).

Given that the intestinal epithelium and the liver are directly exposed to bacteria and their products, we hypothesized that specific microbiota-derived metabolites can modulate LRH-1 activity, thereby influencing intestinal and hepatic physiology. To explore a potential cross-talk between intestinal bacteria and LRH-1, and evaluate the ability of bacterial metabolites to serve as ligands for LRH-1, we screened crude lipid extracts from various bacterial strains for their ability to modulate LRH-1 transcriptional activity. From *Bifidobacterium animalis* subsp. *lactis* B420^TM^ extracts, which transactivated LRH-1, we isolated and identified the fatty acid vaccenic acid (VA, C18:1) as a bacterial LRH-1 ligand. Both the *cis*- and *trans*-isomers of VA directly bound to the LRH-1 ligand-binding domain (LBD) and stimulated LRH-1 transcriptional activity. Accordingly, VA was able to regulate LRH-1 target genes in the intestinal epithelium and the liver. The significance of VA-induced LRH-1 regulation was further validated in a mouse model of diet-induced metabolic syndrome, where VA administration improved glucose homeostasis and reduced hepatic lipogenesis. Our results establish a novel biochemical link between microbiota-derived metabolites, LRH-1 activation and regulation of metabolic processes, suggesting an interesting chemical crosstalk between the intestinal microbiome and LRH-1. They further highlight the therapeutic potential of microbiome-derived compounds in the LRH-1-regulated management of metabolic diseases.

## Results

### *Bifidobacterium animalis* subsp. *lactis* B420 lipid extracts stimulate LRH-1 transcriptional activity

**Figure EV1:**
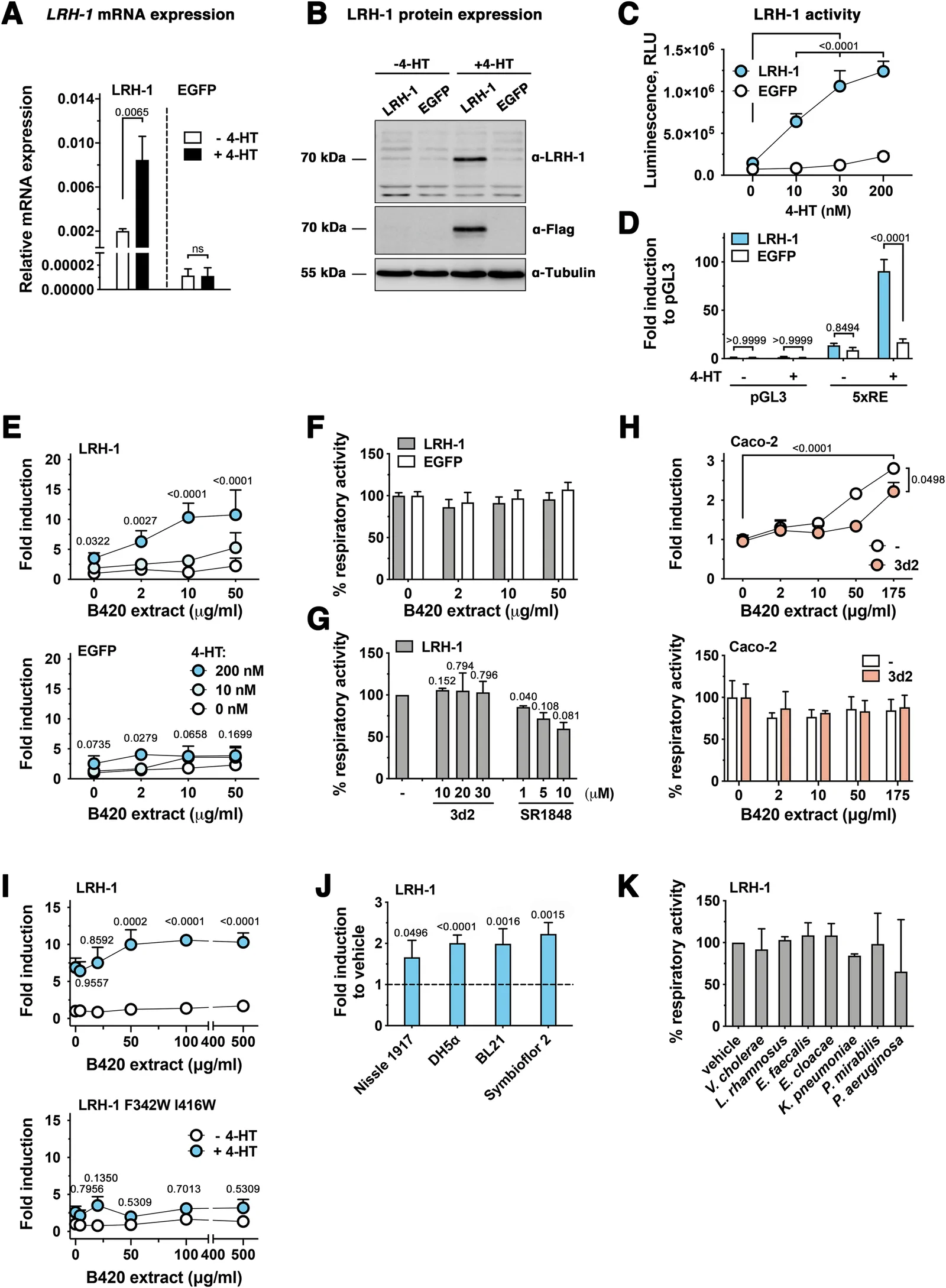
(related to Fig. 1): Characterization of the 4-HT-inducible expression system and control experiments related to LRH-1 transactivation. All experiments were performed in LS174T cells, except those shown in panel H. LRH-1 and EGFP cells refer to cells in which human Flagged-tag LRH-1 or EGFP expression was induced by 4-HT treatment (4 h, unless differently indicated). *P* values are indicated in the figures. (**A**) Quantification of human *LRH-1* mRNA in LRH-1 or EGFP cells. Cells were treated with 4-HT for 6 h and *LRH-1* mRNA was measured by RT-qPCR. A representative result showing mean values ± SD of three replicates of two independent experiments is shown. *P* values were calculated with a two-tailed *t* test, ns: not significant (*P* = 0.9514). (**B**) Immunoblots of human LRH-1 in cell lysates after expression of LRH-1 or EGFP with 4-HT. Cells were treated for 6 h with 4-HT or with its vehicle. LRH-1 expression was detected with anti-LRH-1 or anti-Flag antibodies. Tubulin was used as loading control. (**C**) LRH-1 transcriptional activity in LRH-1 or EGFP cells after o.n. induction with the indicated concentrations of 4-HT. Mean values ± SD of technical triplicates are shown. Two-way ANOVA, *P* values compared to vehicle-treated sample are indicated. RLU relative luciferase units. (**D**) Luciferase activity in LRH-1 or EGFP cells transfected with the pGL3 control vector or LRH-1 reporter. Mean values ± SD. Two-way ANOVA. (**E**) LRH-1 transcriptional activities in cells with 4-HT-inducible LRH-1 or EGFP expression, stimulated o.n. with B420 extract. Mean values of technical triplicates ± SD of a typical experiment (*n* = 2) are shown. Two-way ANOVA. (**F**) Cell viability of LRH-1 and EGFP cells treated with increasing concentrations of B420 extracts. Respiratory activity as measured by MTT is shown (mean ± SD of a representative experiment, *n* = 3). Comparison of all means by two-way ANOVA revealed no significant differences among any of the groups (all *P* > 0.1609). (**G**) Cell viability (MTT assay) of LRH-1 cells treated with the LRH-1 inhibitors 3d2 or SR1848. Mean values ± SD of a representative experiment (*n* = 2) are shown. Paired two-tailed *t* tests were performed to compare each treatment with the control (−). (**H**) Transcriptional activities of endogenous LRH-1 and cell viability (respiratory activity, MTT assay) in Caco-2 cells stimulated with B420 extracts in the absence or presence of the LRH-1 inhibitor 3d2. Mean ± SD (*n* = 2). Two-way ANOVA analysis. Comparison of all cell viability means revealed no differences among any of the groups (all *P* > 0.5292). (**I**) LRH-1 transcriptional activities in LS174T with 4-HT-inducible expression of wild-type LRH-1 or LRH-1 F342W/I416W stimulated with increasing doses of B420 extract. Mean ± SD of one experiment performed in triplicates. Two-way ANOVA (comparison of mean values of B420 treatments to vehicle-treated control, +4-HT group). (**J**) Effect of lipid extracts (50 μg/mL) from different *E. coli* strains on LRH-1 transcriptional activity in LRH-1-expressing LS174T cells. Mean ± SD of at least three independent experiments performed in technical triplicates. Unpaired two-tailed *t* tests were performed to compare each treatment with the control. (**K**) Cell viability (MTT assay) in LRH-1-expressing LS174T cells treated with lipid extracts from bacterial strains indicated (200 μg/mL).

To explore the hypothesis that metabolites derived from intestinal bacteria in general, and probiotic in particular, may act as LRH-1 ligands and thereby modulate LRH-1-mediated transcriptional regulation of these processes, we first investigated whether lipid extracts from *Bifidobacterium animalis* subsp. *lactis* B420^TM^ (hereafter B420) could modulate LRH-1 activity using a reporter assay. We generated LS174T cells that overexpress human Flag-tagged LRH-1 upon induction with 4-hydroxytamoxifen (4-HT) (Fig. EV1A,B), and transiently transfected them with a luciferase reporter plasmid containing 5 repeats of the LRH-1 response element (5xRE). This colorectal epithelial cell line, unlike hepatic cell lines, has the advantage of low endogenous LRH-1 expression, making it well suited for investigating the effects of induced LRH-1 overexpression.

Induction of LRH-1 expression by 4-HT led to a dose-dependent activation of the 5xRE reporter (Fig. EV1C), whereas no significant effects were observed in a control cell line with 4-HT-induced expression of EGFP, or in cells transfected with the empty luciferase reporter control plasmid pGL3 (Fig. EV1D).

**Figure 1:**
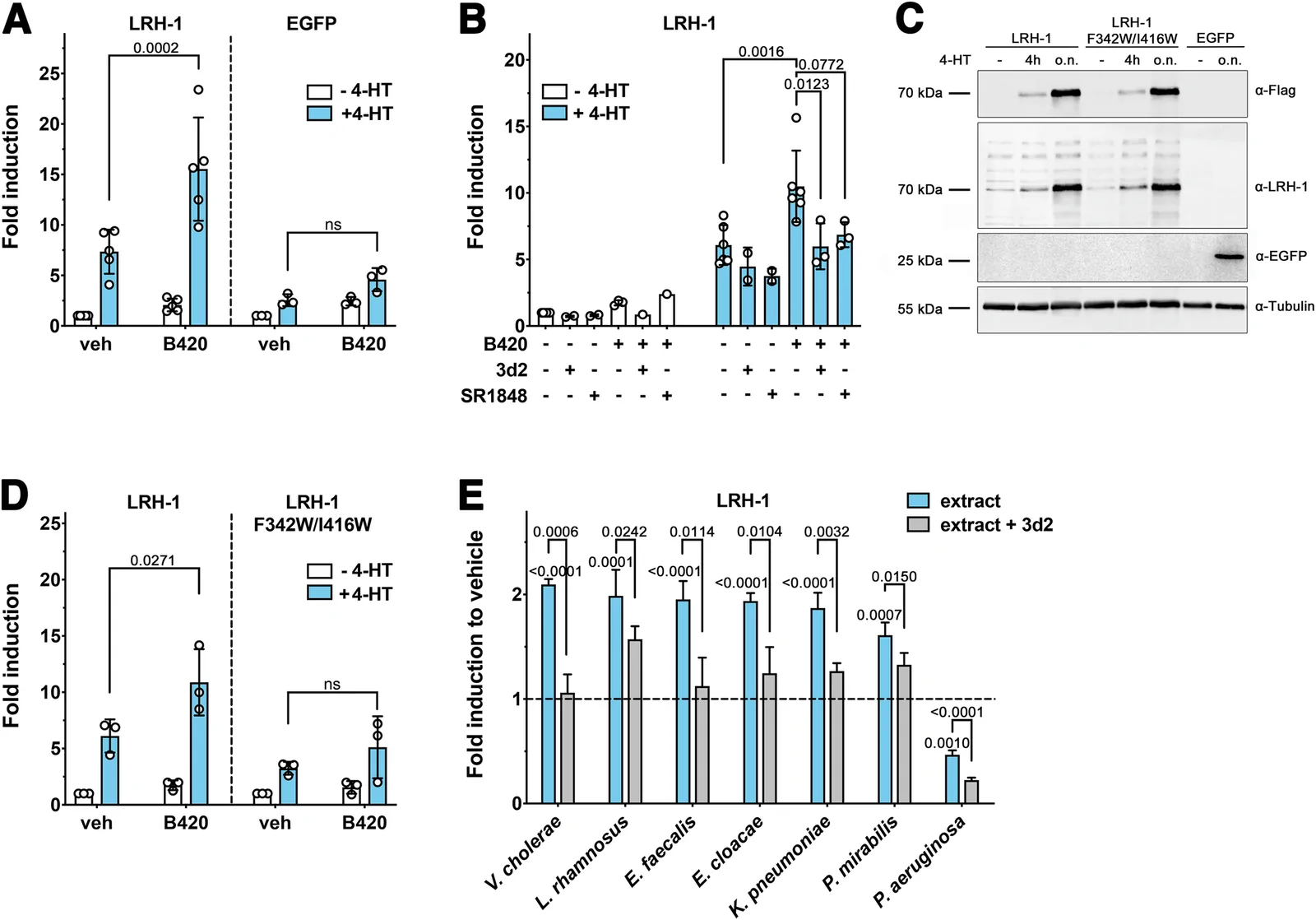
Bacterial lipid extracts modulate LRH-1 transcriptional activity. (**A**, **B**) LS174T cells with inducible expression of human Flag-tagged LRH-1 or EGFP were transfected with the LRH-1 response element luciferase reporter (5xRE), and treated with buffer control (-4-HT) or 200 nM 4-HT to induce LRH-1 or EGFP expression. Cells were then stimulated with vehicle control (veh, ethanol) or 50 μg/mL *Bifidobacterium animalis* subsp. *lactis* B420^TM^ extracts (B420) (**A**). B420-stimulated LRH-1 activity after treatment with the LRH-1 inhibitors 3d2 (10 μM) and SR1848 (1 μM) (**B**). Data are presented as mean ± SD, where dots represent values of single independent experiments (*n* = 3–6 experiments). Statistical differences were analyzed by two-way ANOVA analysis with Tukey´s multiple comparisons test. *P* values are indicated in the figures, ns: not significant (**A**: *P* = 0.9551). (**C**) LRH-1 or EGFP expression analyzed by Western blotting after induction of human Flag-tagged wild-type LRH-1, Flag-tagged F342W/I416W mutant LRH-1 or EGFP with 4-HT for 4 h or overnight (o.n.). Tubulin was used as loading control. (**D**) LRH-1 transcriptional activity in LS174T cells with inducible expression of human Flag-tagged wild-type LRH-1 or F342W/I416W mutant after treatment with B420 extracts (50 μg/mL) or veh control (*n* = 3). Two-way ANOVA with Tukey´s multiple comparisons test, ns: not significant (*P* = 0.9001). (**E**) LS174T cells treated with 4-HT (4 h) to induce expression of LRH-1 were stimulated with 200 μg/mL lipid extracts of the indicated bacterial strains, with or without pretreatment with 3d2, or DMSO control. LRH-1 reporter luciferase activity was normalized to vehicle-treated controls. Means of at least technical triplicates ± SD of a representative experiment (*n* = 2) are shown. Statistical differences were analyzed using multiple unpaired *t* tests. Source data are available online for this figure.

Lipid extracts isolated from B420 at a concentration of 50 μg/mL strongly enhanced LRH-1 reporter activity in LRH-1-overexpressing cells (Fig. 1A). This effect was minimal in EGFP-overexpressing cells and can be attributed to the low levels of endogenous LRH-1 in LS174T cells that can be detected by RT-PCR (Fig. EV1A). LRH-1 transactivation by B420 extracts was dose-dependent and more pronounced at higher levels of 4-HT-induced LRH-1 expression (Fig. EV1E).

To confirm the specificity of LRH-1 in the B420-induced transcriptional activation, we used two well-characterized LRH-1 pharmacological inhibitors, 3d2 and SR1848. 3d2 binds to the LRH-1 LBD, induces a conformational change that impairs its interaction with co-regulatory proteins (Benod et al, 2013) and reduces LRH-1 basal transcriptional activity in cell lines (Michalek et al, 2022). SR1848 promotes rapid translocation of LRH-1 from the nucleus to the cytoplasm (Corzo et al, 2015). Both molecules effectively reduced the B420-stimulated LRH-1 activity (Fig. 1B), suggesting that the stimulatory effect of B420 is dependent on LRH-1. Neither B420 extracts nor the LRH-1 inhibitors affected cell viability, as monitored by measuring cellular respiration by MTT assay (Fig. EV1F,G). To evaluate whether endogenous LRH-1 could also be activated by the bacterial extracts, we employed human Caco-2 cells, which express higher levels of endogenous LRH-1 (Sidler et al, 2011). Again, we could confirm that B420 extracts dose-dependently stimulated LRH-1 transcriptional activity, which was inhibited by 3d2 (Fig. EV1H).

In order to investigate whether the effect of B420 extracts on LRH-1 transcriptional activity was mediated via binding of potential ligands to the LBP, we made use of a mutant LRH-1, in which the mutations F342W and I416W prevent ligand interaction with the LBP (Ortlund et al, 2005; De Jesus Cortez et al, 2016; Lee et al, 2011). The Flag-tagged mutant LRH-1 F342W/I416W protein was induced in LS174T cells, resulting in comparable expression levels to those of wild-type LRH-1 after 4 h and overnight induction (Fig. 1C). Expression of mutant LRH-1 reduced basal activity and markedly impaired responsiveness to B420 extracts (Figs. 1D and  EV1I), indicating that B420 extracts contain ligands that directly activate LRH-1.

Interestingly, the LRH-1-activating capacity was not restricted to B420 nor limited to probiotic strains, as extracts from various Gram-negative and Gram-positive commensal and pathogenic bacteria also promoted LRH-1 transactivation. Among the strains tested (*Lactobacillus rhamnosus*, *Proteus mirabilis*, *Enterobacter cloacae*, *Vibrio cholerae*, *Klebsiella pneumoniae*, *Enterococcus faecalis, Pseudomonas aeruginosa*), all extracts modulated LRH-1 activity (Fig. 1E). LRH-1 transcriptional activity was stimulated by all bacterial extracts, with the exception of extracts from *P. aeruginosa*. Pre-treatment with the LRH-1 inhibitor 3d2 reduced this response to varying degrees. Additionally, lipid extracts from different *Escherichia coli* strains, including commonly used laboratory strains, also transactivated LRH-1 (Fig. EV1J). Interestingly, extracts from *P. aeruginosa* inhibited LRH-1 transactivation, which may be partly attributable to a decrease in reporter cell viability (Fig. EV1K). No major effects on cell viability were observed for the other bacterial extracts.

In summary, our findings demonstrate that lipid ligands capable of activating LRH-1 transcriptional activity are present in different human gut bacteria, including the probiotic strain B420.

### Identification of vaccenic acid as a novel LRH-1-activating ligand

**Figure EV2:**
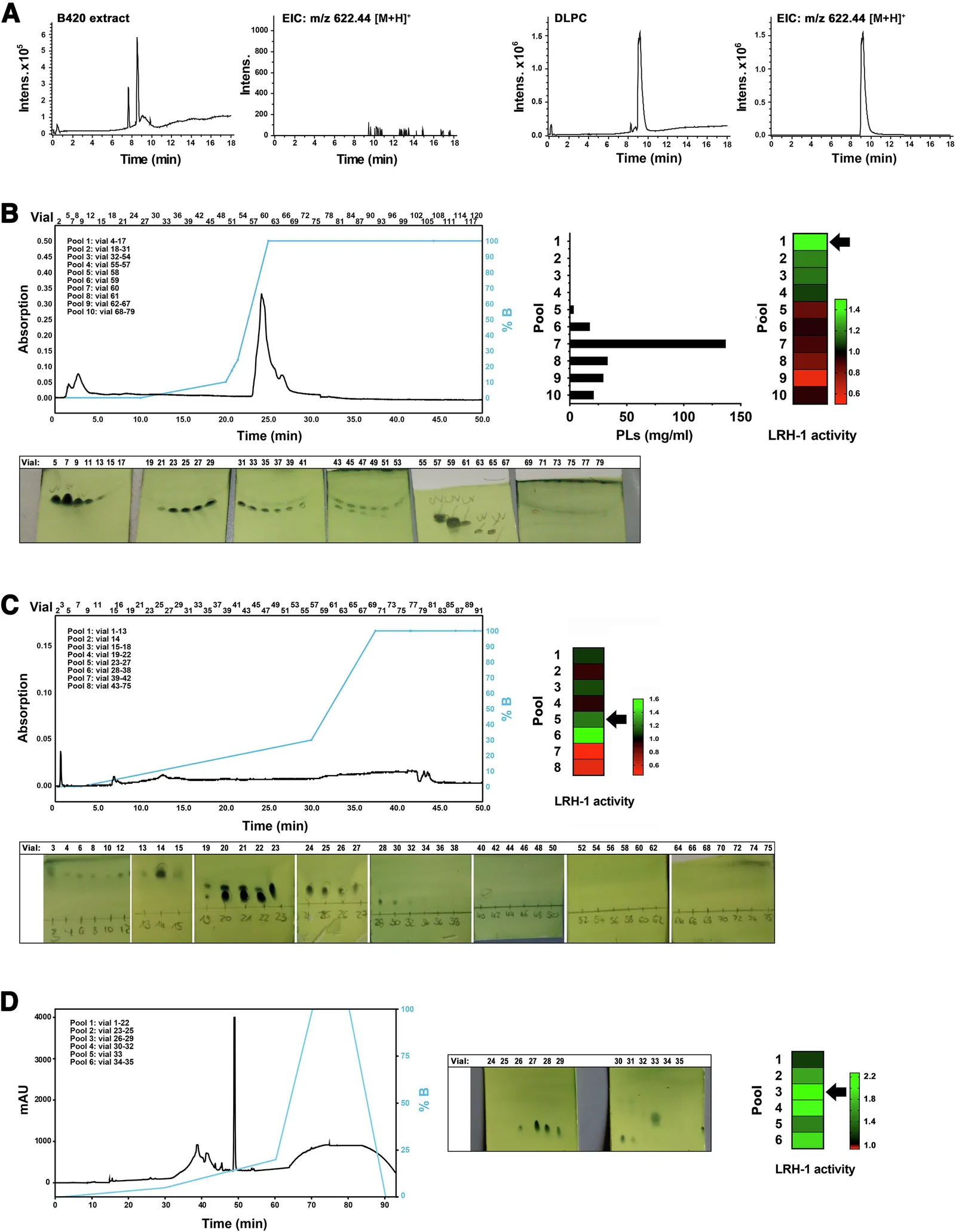
(related to Fig. 2): Chromatographic analysis of B420 extracts and identification of LRH-1-stimulating compounds. (**A**) Chromatograms and extracted ion chromatograms (EIC) for *m/z* 622.44 (right panel) of the complete B420 extract (left) or of DLPC (right). A MicrOTOF II ESI mass spectrometer was used with an electrospray ionization (ESI) source operating in positive ion mode. Parameters were set as follows: capillary voltage: 4500 V; nebulizer pressure: 12 bar; drying temperature: 180 °C; drying gas flow: 10 l/min; End plate offset: 80 V. (**B**) Silica flash column chromatography of crude B420 extract using a 0–100% gradient of chloroform (**A**) and chloroform/methanol 7:3 (**B**) in 50 min and a flow rate of 40 ml/min. Detection at 254 nm. An aliquot of every second vial was analyzed by thin layer chromatography (TLC) using phosphomolybdic acid stain. Vials with similar content were combined as indicated resulting in ten pools. Analysis of phospholipid (PL) content in these pools revealed that PL content peaked in pool 7, whereas pool 1 (arrow) promoted the strongest LRH-1 transactivation. (**C**) Silica flash column chromatography of pool 1 using a 0–100% hexane and ethyl acetate gradient (% B) in 50 min and a flow rate of 30 mL/min. Individual vials were collected and analyzed by TLC using phosphomolybdic acid stain. Vials with similar content were combined into 8 pools as indicated. Pools 5 and 6 showed strongest LRH-inducing activity. (**D**) Pool 5 was further purified by HPLC (Shimadzu LC20-AT) with a preparative Reprosil 100 silica column using hexane and ethyl acetate as mobile phase (0–100% in 90 min, % B) and a flow rate of 4 mL/min. Vials containing lipids were combined into six pools as indicated and tested for LRH-1 transactivation. Pool 3 (arrow) was further purified by a second HPLC (see text and Fig. 2A, B).

While an increasing number of compounds have been described to bind to and/or activate LRH-1, at present a definite physiological ligand remains elusive. Notably, the synthetic lipid DLPC was shown to bind to the LRH-1 LBD and to modulate its activity both in cell lines and mice (Lee et al, 2011; Musille et al, 2012). To explore whether DLPC might be responsible for the observed effects, mass spectrometry of B420 extracts was performed. The analysis of the chromatogram indicated the absence of DLPC, since the corresponding mass (622.44 *m/z*) could not be identified (Fig. EV2A).

**Figure 2:**
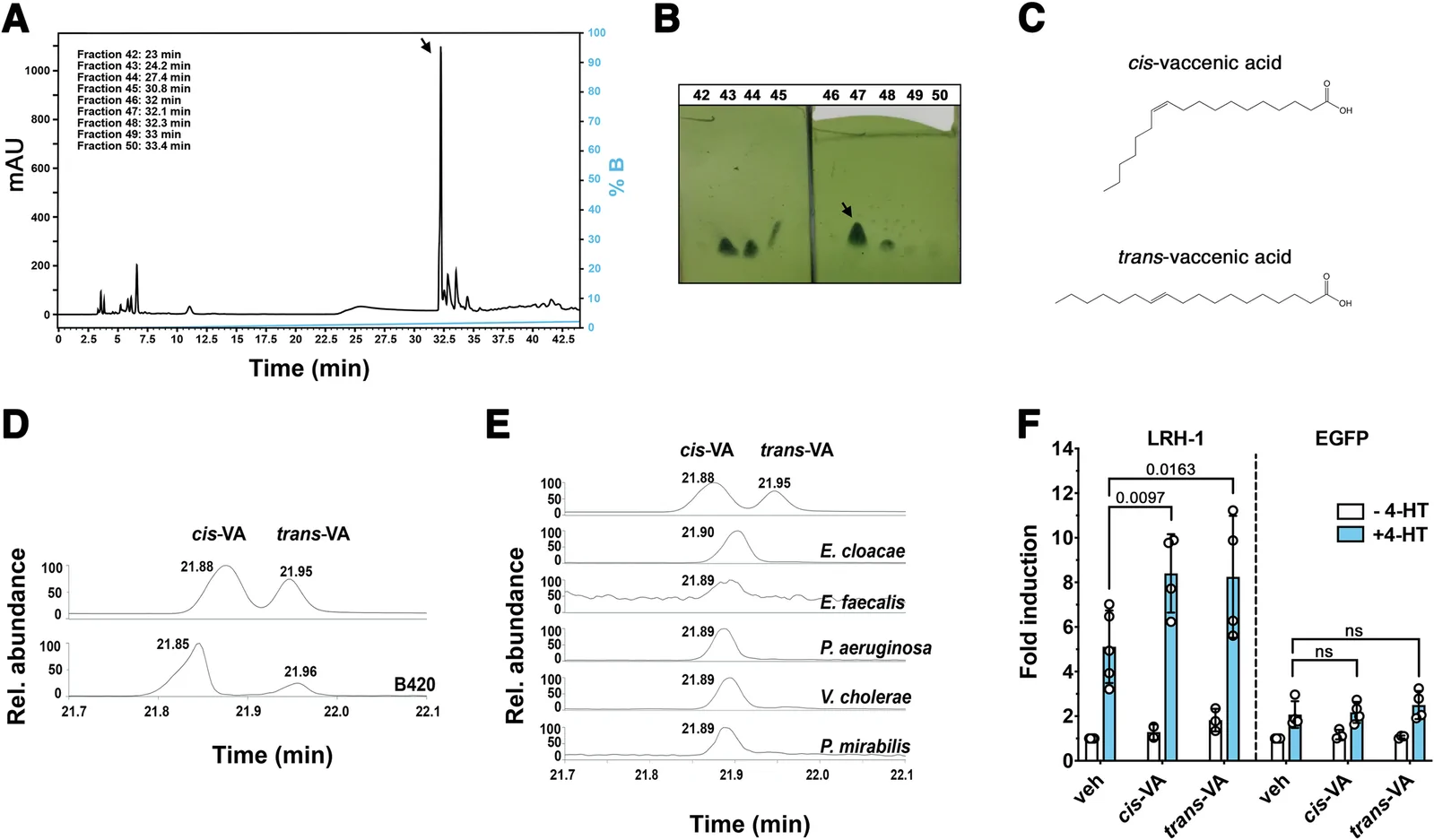
The monounsaturated fatty acid vaccenic acid is present in different bacteria and stimulates LRH-1 transcriptional activity. (**A**, **B**) Purification of pool 1.5.3 by HPLC with dichloromethane (**A**)/methanol (**B**) as mobile phase (0–2.5% B in 45 min) in a flow rate of 4 mL/min, detection at 254 nm (**A**). Active fraction 1.5.3.47, eluted at 1% B after 32.1 min (arrow), and showed a single spot after TLC analysis (**B**). (**C**) Chemical structure of *cis*- and *trans*-vaccenic acid (VA). (**D**) Analysis of *cis*- and *trans*-VA FAMEs in B420 extracts (bottom panel). Top panel: reference lipids. (**E**) Analysis of *cis*- and *trans*-VA FAMEs in the indicated bacteria extracts (top panel: reference lipids). (**F**) LRH-1 transcriptional activities in LS174T cells treated with 4-HT to induce expression of Flag-tagged LRH-1 or EGFP, and subsequently stimulated with 100 μM *cis*-VA or *trans*-VA, or the vehicle (ethanol, veh). Luciferase reporter activity was normalized to the vehicle-treated condition in the absence of 4-HT. Data are presented as mean ± SD of *n* = 3–5 experiments, where dots represent values of individual experiments. Two-way ANOVA analysis with Tukey´s multiple comparisons test, ns: not significant (*P* > 0.9999). Source data are available online for this figure.

In the attempt to isolate and identify the bioactive molecules present in the B420 extracts, we combined chromatographic separation of lipid classes with screening for activity on LRH-1 transactivation using the luciferase reporter assay (Fig. EV2B–E). The B420 extract was separated by silica flash column chromatography over 50 min, resulting in 120 vials. A small sample from every second vial was analyzed by thin-layer chromatography (TLC) and vials with similar retention factor (R*f*) were combined resulting in ten pools. These were evaporated, resuspended in ethanol and used to screen for LRH-1 transactivation in Caco-2 cells transfected with the 5xRE LRH-1 luciferase reporter, which express high levels of endogenous LRH-1 compared to LS174T cells (Fig. EV2B). Pool 1, which induced the highest LRH-1 transactivation, was further fractionated with a second silica flash column chromatography. This active pool did not contain phospholipids (PLs), which were collected in pool 5 to 10 (Fig. EV2B). As in the previous step, individual vials were collected, combined into eight pools based on TLC analysis, and pooled fractions were tested for LRH-1 activity in Caco-2 cells (Fig. EV2C). The most active pool 1.5 was further purified by HPLC (Fig. EV2D). Samples were combined into six pools based on TLC analysis and tested for LRH-1 transactivation. The active pool 1.5.3 was further purified by a second HPLC, and fraction 1.5.3.47, eluting with a clear peak at 32.1 min (Fig. 2A) and showing a strong TLC spot (Fig. 2B), was analyzed by 1D NMR (^1^H, ^13^C), 2D NMR (COSY, HMBC, HSQC) and GC-MS (Appendix Figs S1–S7). These identified the bioactive compound as vaccenic acid (VA, Δ^11^-octadecanoic acid), a C18:1 fatty acid, present in both *cis* and *trans* isomeric forms (Fig. 2C).

To confirm the presence of *cis*- and *trans*-VA in active B420 extracts, we analyzed these fatty acids as FAMEs (fatty acid methyl esters) by GC-MS/MS. Comparison of retention times and fragmentation patterns with a reference mixture of *cis*- and *trans*-VA methyl esters confirmed the presence of both isomers in B420 extracts (Fig. 2D). We further investigated the presence of *cis*- and *trans*-VA in the other bacterial extracts that showed LRH-1 transactivation, namely *P. mirabilis*, *E. cloacae*, *V. cholerae*, *E. faecalis*, and *P. aeruginosa*. *Cis*-VA was found in all bacteria extracts tested, whereas the *trans* isomer was only detected in B420 (Fig. 2E).

**Figure EV3:**
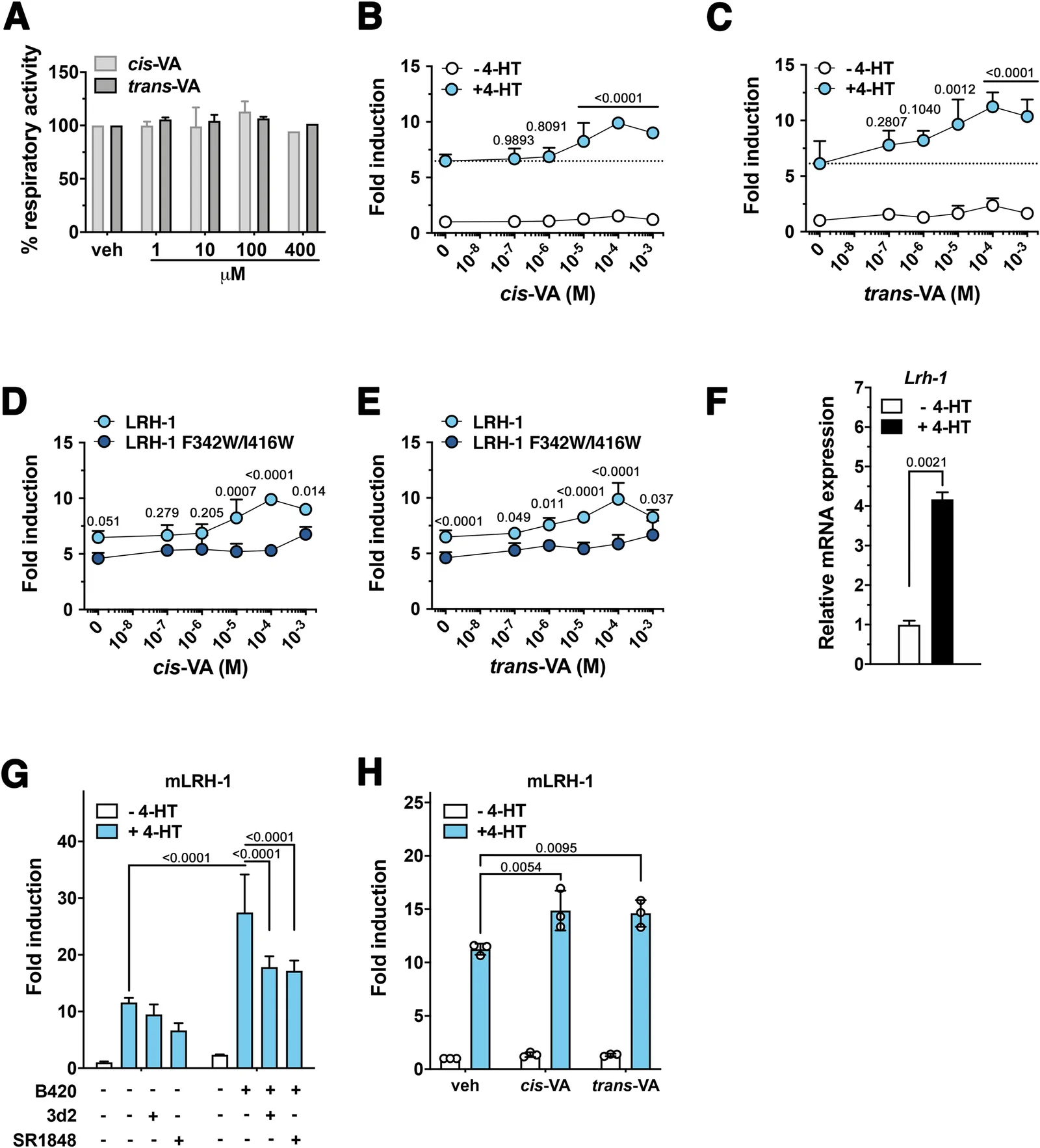
(related to Fig. 2): Characterization of *cis*- and *trans*-VA-mediated LRH-1 transactivation; verification of murine LRH-1 transactivation by B420 and VA. Experiments were performed in LS174T cells with 4-HT-inducible expression of wild-type or mutant human Flagged-tag LRH-1, or murine LRH-1 (4 h, unless differently indicated). (**A**) Cell viability (MTT assay) in LRH-1 cells treated with the indicated concentrations of *cis*- or *trans*-VA. Mean ± SD of two independent experiments performed in duplicates. (**B**,** C**) Effects of *cis*- (**B**) or *trans*-VA (**C**) on the transcriptional activity of LRH-1 in cells with uninduced (-4-HT) or induced expression of LRH-1 ( + 4-HT). Mean values of technical triplicates ± SD of one representative experiment (*n* = 3) are shown. Two-way ANOVA. (**D**, **E**) Effects of *cis*- (**D**) or *trans*-VA (**E**) on the transcriptional activity of wild-type or F342W/I416W mutant LRH-1. Mean values of technical triplicates ± SD of one experiment are shown. Two-way ANOVA comparing LRH-1 vs LRH-1 F342W/I416W for each concentration. (**F**) Quantification of murine *Lrh-1* mRNA by RT-qPCR in mLRH-1 with 200 nM 4-HT for 6 h. Mean ± SD of one experiment performed in duplicates. Unpaired two-tailed *t* test. (**G**,** H**) LRH-1 transcriptional activity in cells with 4-HT-inducible expression of mLRH-1. Cells were incubated with 200 μg/mL B420 extract (**G**), with 100 μM *cis*-VA or *trans*-VA (**H**) or the vehicle (ethanol, veh) for 16 h. For inhibition of LRH-1 activity, cells were treated with 3d2 or SR1848. Mean ± SD of one experiment performed in triplicates (**G**) or of three independent experiments in triplicates (**H**). Two-way ANOVA.

As the active fraction 1.5.3.47 contained both *cis*- and *trans*-VA, the individual isomers were purchased and tested separately for LRH-1 transactivation and potential cytotoxicity (Fig. EV3A). Both VA isomers stimulated LRH-1 transcriptional activity in LRH-1-overexpressing LS174T cells, whereas no activation was observed in EGFP-expressing control cells (Fig. 2F). The response to VA was dose-dependent with lowest activities already detected at 0.1–1 μM and maximal effect at a concentration of 100 μM (Fig. EV3B,C). Basal activity and responsiveness to both *cis*- and *trans*-VA was reduced in mutant LRH-1 F342W/I416W (Fig. EV3D,E) compared to wild-type LRH-1, validating the hypothesis that VA directly engages the LBP of LRH-1.

The murine LRH-1 protein is considered constitutively active and less dependent on agonist binding compared to human LRH-1 (Sablin et al, 2003; Krylova et al, 2005). Nevertheless, many studies have demonstrated that LRH-1 modulation by agonistic molecules is also effective in mice (Cobo-Vuilleumier et al, 2018b; Mays et al, 2022; Lee et al, 2011). For this reason, we were interested in understanding whether murine LRH-1 activity could also be modulated by bacterial extracts and VA. In LS174T cells with inducible mLRH-1 overexpression (Fig. EV3F), transactivation of murine LRH-1 was induced by B420 extracts, and inhibited by 3d2 and SR1848 (Fig. EV3G). Both *cis*- and *trans*-VA stimulated murine LRH-1 activity, although to a lesser extent than human LRH-1 (Fig. EV3H).

In conclusion, our results identify the fatty acids *cis*- and *trans*-VA present in bacterial extracts as novel ligands for LRH-1, and demonstrate their ability to modulate LRH-1 activity in both human and murine models.

### Binding to and modulation of LRH-1 activity by VA in comparison to other long-chain fatty acids

**Figure 3:**
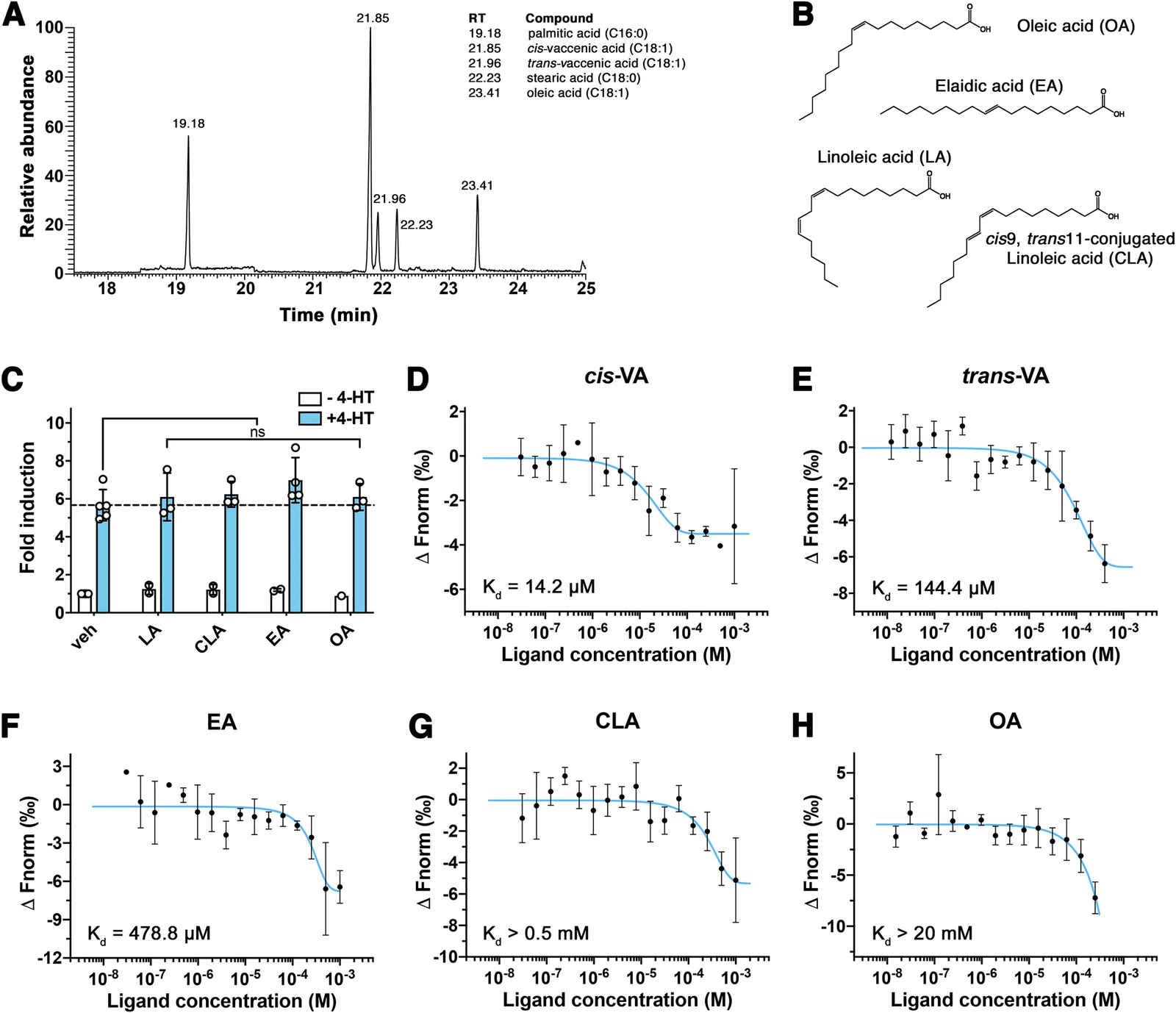
Specific binding and modulation of LRH-1 activity by vaccenic acid compared to other long-chain fatty acids. (**A**) Fatty acid composition of B420 after lipid extraction and total ion GC-MS. The peak labels represent the retention times (RT) of the identified FAME components indicated. (**B**) Chemical structures of oleic acid (OA), elaidic acid (EA), linoleic acid (LA) and *cis*9, *trans*11-conjugated linoleic acid (CLA). (**C**) LS174T cells with inducible LRH-1 expression were treated with buffer control or 4-HT and then stimulated with the indicated fatty acids (100 μM). Data are presented as mean ± SD, where dots represent values of single independent experiments (*n* = 1–5). Two-way ANOVA analysis, ns: not significant. (**D**–**H**) Microscale thermophoresis (MST) dose-response curves and K_d_ of LRH-1 LBD and the indicated fatty acids. The change in normalized fluorescence (∆Fnorm) was derived by analyzing the 1 s region before the T-Jump and 0.5–1.5 s after the T-Jump in the MST traces (Fig. EV4D), and was plotted against the concentrations of ligand. K_d_ values were derived with the K_d_ model offered in the MO. Affinity Analysis software. Mean ± SD of three independent measurements. Source data are available online for this figure.

As bacteria can produce different fatty acids, we were interested in which other long-chain fatty acid species could be detected in B420 and whether they could also induce LRH-1 activity. Thus, fatty acids were extracted from B420 and analysed with GC-MS/MS after derivatization to their methyl-ester forms. Besides confirming the presence of *cis*- and *trans*-VA (C18:1), several other long-chain fatty acids, including palmitic acid (PA, C16:0), stearic acid (SA, C18:0), and oleic acid (OA, C18:1), were identified as the main lipid species (Fig. 3A). This profile is in accordance with that reported for other *B*. *lactis* strains (Oberg et al, 2013).

**Figure EV4:**
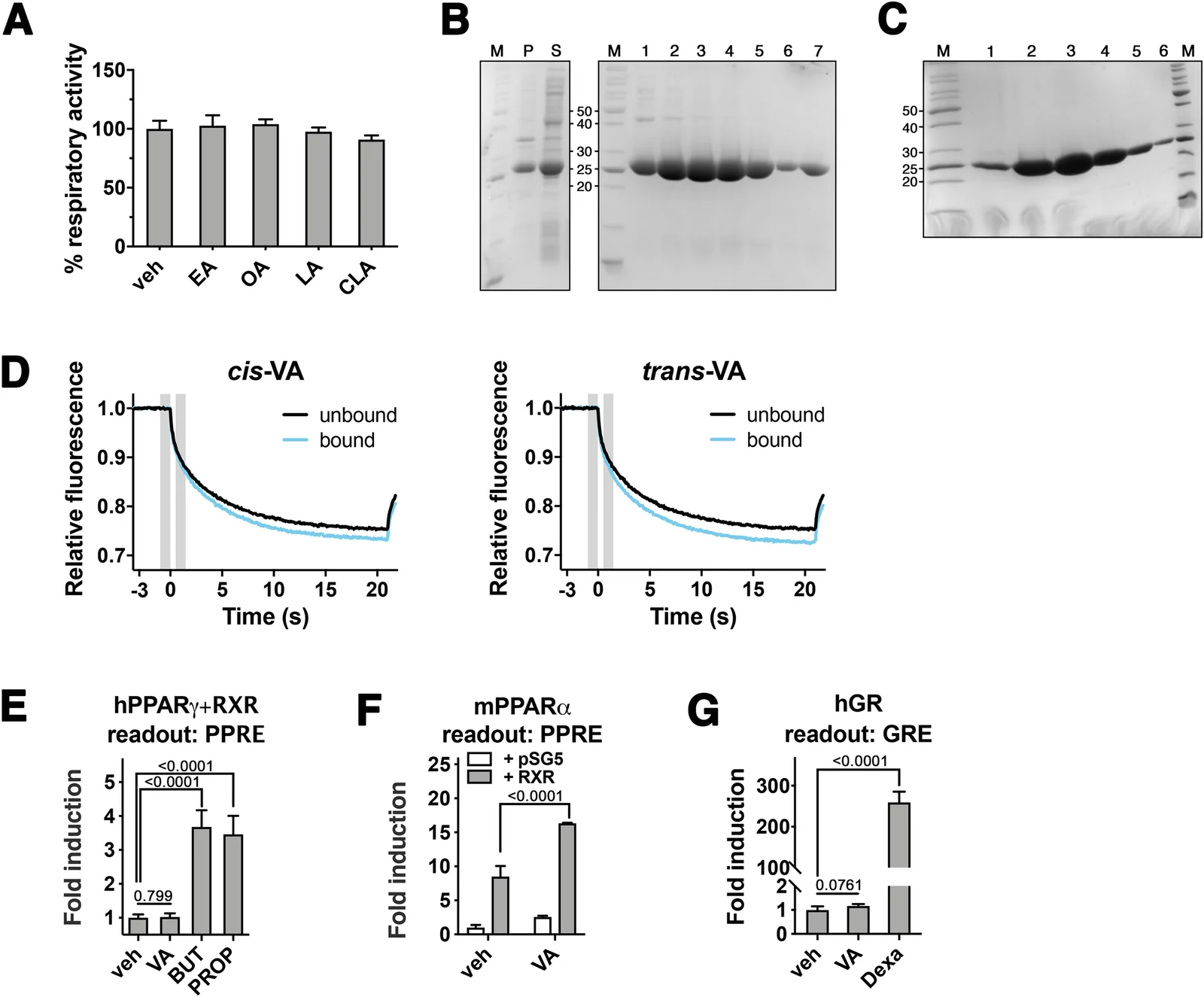
(related to Fig. 3): LRH-1 LBD purification and control experiments. (**A**) Cell viability (MTT assay) of LRH-1 cells treated with different fatty acids (100 μM) (elaidic acid, EA; oleic acid, OA; linoleic acid, LA; *cis*9, *trans*11-conjugated linoleic acid, CLA) or their vehicle (veh, ethanol). Mean values of triplicates ± SD of one experiment. (**B**) Coomassie-stained SDS-PAGE of different fractions obtained after Ni^2+^-affinity chromatography to capture His-tagged LRH-1 LBD and elution with increasing concentrations of imidazole (see “Methods” section). M =  Unstained Protein Standard, Broad Range (10–200 kDa, New England Biolabs); *P* = Bacterial pellet before purification; S = Supernatant before purification; 1–7 = LRH-1 LBD-containing fractions collected with an increasing gradient of imidazole. (**C**) Coomassie-stained SDS-PAGE of fractions of pure LRH-1 LBD after additional subtractive HisTrap chromatography and gel filtration. (**D**) Representative microscale thermophoresis (MST) traces for *cis*- and *trans*-VA. The “bound” curve represents the trace with 1 mM ligand and the “unbound” one the condition with the lowest ligand concentration (30.5 nM). The change in normalized fluorescence was derived by analyzing the 1 s region before the T-Jump and 0.5–1.5 s after the T-Jump in the MST traces (grey bars) and was plotted against the concentrations of ligand. (**E**–**G**) Transcriptional activities in LS174T cells after transfection of the indicated NRs and the cognate response element-containing luciferase reporter. *P* values after statistical analysis are indicated in the figures. (**E**) Human PPARγ/RXR activity after incubation with VA (30 μM, mixture of *cis* and *trans*). Butyrate (BUT) and propionate (PROP) (3 mM) were used as positive controls. PPRE PPARγ Response Element. Unpaired *t* test. (**F**) Murine PPARα activity after cotransfection with RXR or empty vector pSG5 control, and incubation with VA (30 μM, mixture of *cis* and *trans*). PPRE: PPAR Response Element. Two-way ANOVA. (**G**) Transcriptional activity of the glucocorticoid receptor (GR) after incubation with VA (150 μM, mixture of *cis* and *trans*) or 500 nM dexamethasone (Dexa) (GR agonist). Unpaired *t* test. GRE Glucocorticoid Response Element.

We then sought to determine whether unsaturated C18 fatty acids might exhibit similar LRH-1-stimulating activity to VA. OA contains one double bond between C9 and C10 in *cis* configuration, while elaidic acid (EA) is the *trans*-isomer of OA at the same position. Linoleic acid (LA) is a polyunsaturated fatty acid with two double bonds at position 9 and 12, both in *cis* configuration, whereas conjugated linoleic acid (CLA, *cis*-9, *trans*-11) is derived from *trans*-VA by the action of stearoyl-CoA desaturase in tissue (Reynolds et al, 2008) (Fig. 3B). Interestingly, none of these fatty acids stimulated LRH-1 activity in the LRH-1 reporter assay in LS174T cells (Fig. 3C), nor did they affect cell viability (Fig. EV4A). One possible explanation of the dissimilarity with VA could be that the position or configuration of the double bond, or the presence of an additional double bond, may hinder binding of these fatty acids to the LRH-1 LBP.

To further investigate this hypothesis, we expressed and purified the LBD of human LRH-1 as a His_6_-tagged protein (Fig. EV4B,C) and quantified the binding affinity of the different long-chain fatty acids with microscale thermophoresis (MST). The analysis confirmed binding of *cis*- and *trans*-VA to LRH-1 LBD at dissociation constants (K_d_) of 14.2 μM and 144.4 μM, respectively, whereas the other fatty acids tested did not show binding at relevant K_d_ (Figs. 3D–H and  EV4D).

Saturated and unsaturated fatty acids are well-known regulators/activators of the peroxisome proliferator-activated receptor (PPAR) transcription factors of the NR superfamily, namely PPARα (NR1C1), PPARβ/δ (NR1C2), and PPARγ (NR1C3). These NRs form heterodimers with their obligate partner RXR (retinoid X receptor), and bind to specific DNA sequences, known as PPAR response element (PPRE), thereby regulating lipid metabolism and inflammation (reviewed in (Varga et al, 2011)). In our experiments, VA failed to transactivate PPARγ/RXR heterodimers, but was able to stimulate transcriptional activity of PPARα, which was notably enhanced by cotransfection with RXR. No effect was observed on the transcriptional activity of the glucocorticoid receptor (GR, NR3C1) (Fig. EV4E–G). These results highlight the specificity of VA in modulating LRH-1 activity.

### In vivo activation of LRH-1 by VA modulates intestinal and hepatic gene expression

**Figure 4:**
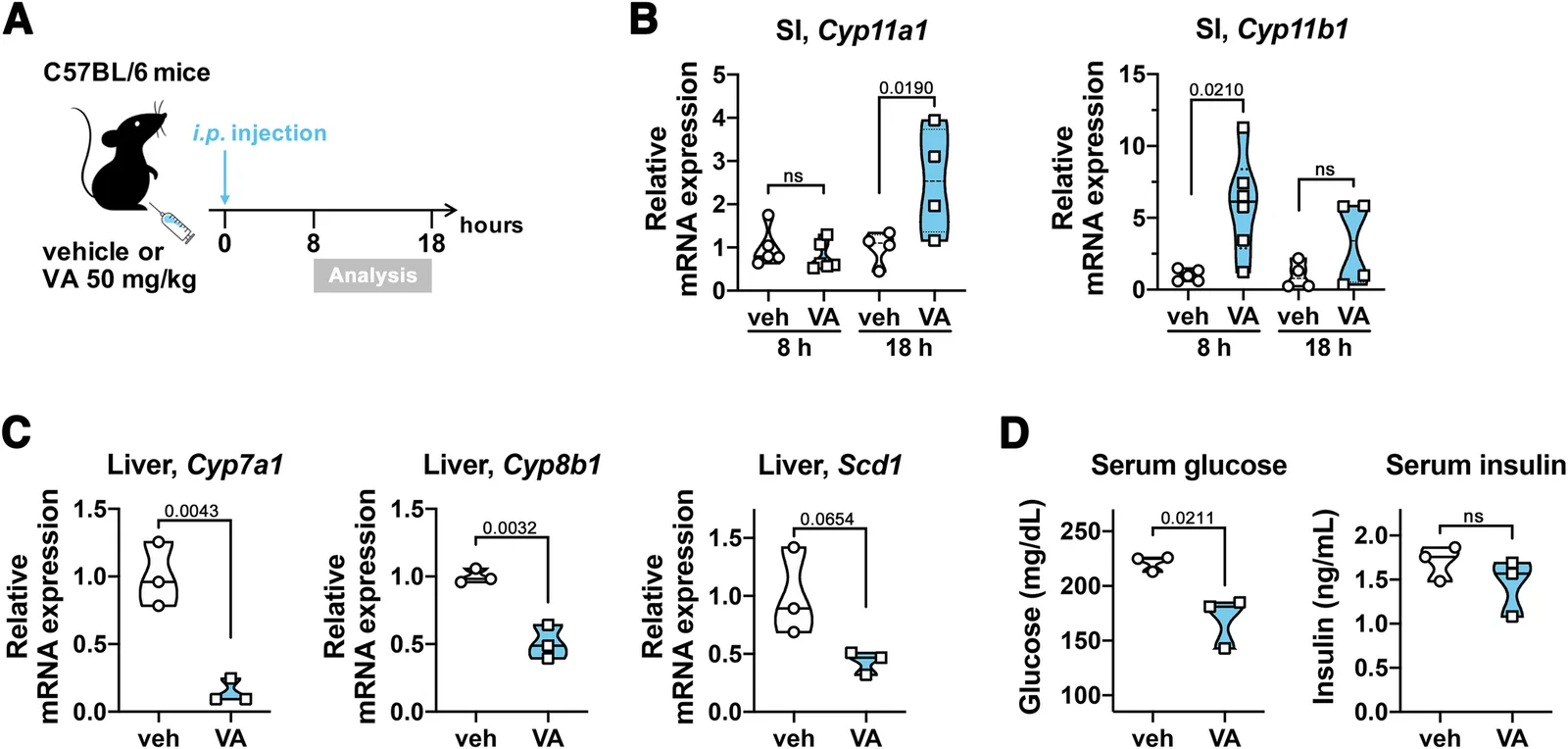
VA regulates expression of intestinal and hepatic LRH-1 target genes. (**A**) Schematic representation of the animal experiment. Male C57Bl/6 mice were injected with a mixture of *cis*- and *trans-*VA (50 mg/kg) or vehicle control intraperitoneally (i.p.). Tissues were collected after 8 or 18 h, and gene expression was analyzed by RT-qPCR. (**B**) VA induced mRNA expression of the steroidogenic enzymes *Cyp11a1* and *Cyp11b1* in intestinal tissue, assessed 8 and 18 h post injection. SI small intestine, veh vehicle. One-way ANOVA (8 h: veh *n* = 5, VA *n* = 6; 18 h: veh *n* = 4, VA *n* = 4), ns: not significant, *P* = 0.9450 (*Cyp11a1*), *P* = 0.5761 (*Cyp11b1*). (**C**) RT-qPCR analysis of the LRH-1 target genes *Cyp7a1*, *Cyp8b1* and of *Scd1* in the liver of male C57Bl/6 mice 18 h after injection of the mixture of *cis*- and *trans*-VA or its vehicle control (unpaired two-tailed *t* test, *n* = 3 per condition). (**D**) Serum glucose and insulin levels ca. 18 h after i.p. injection of VA or its vehicle control (unpaired two-tailed *t* test, ns: not significant, *P* = 0.3049, *n* = 3 per condition). Source data are available online for this figure.

**Figure EV5:**
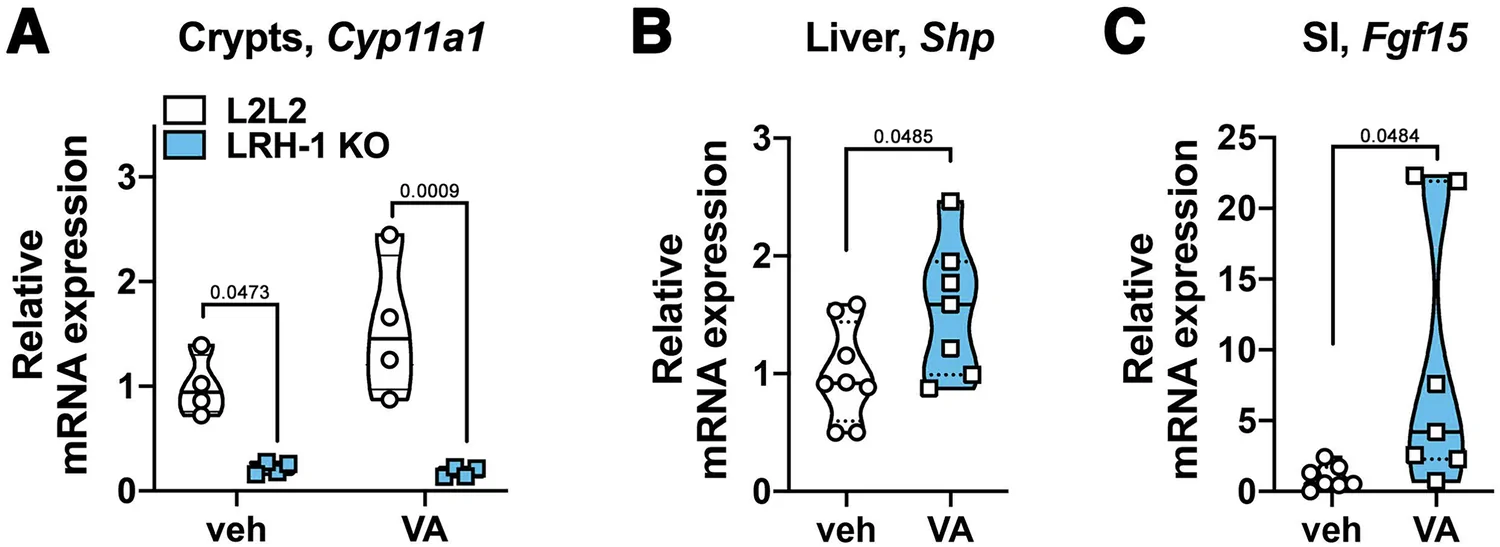
(related to Fig. 4): VA-induced expression of steroidogenic enzymes in primary intestinal crypt cells requires the presence of LRH-1; investigation on FXR-SHP-FGF15 axis contribution to VA-induced effects. (**A**) Primary crypt cells from small intestine (SI) of control LRH-1 L2L2 and LRH-1 VilCre mice with LRH-1 deletion in intestinal epithelial cells (LRH-1 KO) were isolated and cultured for 4 h ex vivo with 240 μM mixture of *cis*- and *trans*-VA or its vehicle control (veh). *N* = 4, two-way ANOVA with Tukey´s correction. Isolation and culture of intestinal crypts was done as described in Noti et al, 2010. (**B**, **C**) Increased expression of hepatic *Shp* (**B**) and intestinal *Fgf15* (**C**) in mice treated for 18 h with VA compared to its vehicle control, suggesting a complex crosstalk between VA-induced LRH-1 activation and the FXR-SHP-FGF15 axis. *n* = 7–8, each dot represents an individual mouse. Unpaired two-tailed *t* test.

LRH-1 is an established regulator of local intestinal GC synthesis via the transcriptional regulation of *Cyp11a1* and *Cyp11b1* (Coste et al, 2007; Mueller et al, 2006). To evaluate whether VA could function as an LRH-1 agonist in vivo, we administered a mixture of *cis*- and *trans*-VA (50 mg/kg) or its vehicle via intraperitoneal (i.p.) injection into male C57Bl/6 mice (Fig. 4A). Mice had *ad libitum* access to food and water. Tissue was collected either 8 h or 18 h post-injection, as indicated in the figure legends. In the small intestine, VA induced expression of both *Cyp11a1* and *Cyp11b1* (Fig. 4B), consistent with previous studies employing LRH-1 agonists (Mays et al, 2019, 2022) and describing *Cyp11a1* and *Cyp11b1* as LRH-1 targets in the intestine (Mueller et al, 2006, 2007). To assess whether these changes require the presence of LRH-1, we employed mice with LRH-1 deletion in intestinal epithelial cells (LRH-1 VilCre) and their littermates with floxed LRH-1 gene, but lacking Cre expression, as controls (LRH-1 L2L2) (Coste et al, 2007; Ahmed et al, 2023). Primary intestinal crypt cells isolated from the small intestine of these mice confirmed reduced *Cyp11a1* expression in LRH-1 KO cells, and showed that VA incubation induced expression of *Cyp11a1* in control LRH-1 L2L2, but not LRH-1 KO cells, although this difference did not reach significance at the condition used (Fig. EV5A).

Activation of LRH-1 in the liver has been associated with improvement of glucose and insulin homeostasis, likely through transcriptional regulation of de novo lipogenesis (Lee et al, 2011; Mays et al, 2022). Specifically, several key lipogenic genes, including sterol regulatory element binding transcription factor 1 (*Srebf1*, also called *Srebp1-c*), acetyl-CoA carboxylase 2 (*Acacb*), fatty acid synthase (*Fasn*), and stearoyl-CoA desaturase-1 (*Scd1*), are downregulated following treatment with the LRH-1 agonists DLPC and 10CA, an optimized derivative of the synthetic LRH-1 agonist RJW100 (Flynn et al, 2018). Similarly, treatment of HepG2 cells with the LRH-1 agonist 6N reduced the expression of *SCD1*, the rate-limiting enzyme in de novo lipogenesis (Mays et al, 2019; Cato et al, 2022).

To assess endogenous LRH-1 transcriptional activity in vivo in response to VA, we measured the hepatic expression of *Scd1*, as well as of the established hepatic LRH-1 targets *Cyp7a1* and *Cyp8b1* in C57Bl/6 mice following a single i.p. injection of VA. Consistent with prior studies, VA administration decreased expression of *Scd1*, as well as *Cyp7a1* and *Cyp8b1* (Fig. 4C). Notably, a single VA injection also significantly lowered serum glucose, although serum insulin levels remained unchanged under these conditions (Fig. 4D). These results indicate that VA can activate endogenous LRH-1 and regulate metabolic pathways in the liver. Interestingly, hepatic *Shp* and intestinal *Fgf15* were upregulated after VA treatment (Fig. EV5B,C), suggesting involvement of FXR signaling and the existence of a complex cross-talk between LRH-1 and the FXR-SHP-FGF15 axis.

### VA administration improves metabolic liver disease

**Figure 5:**
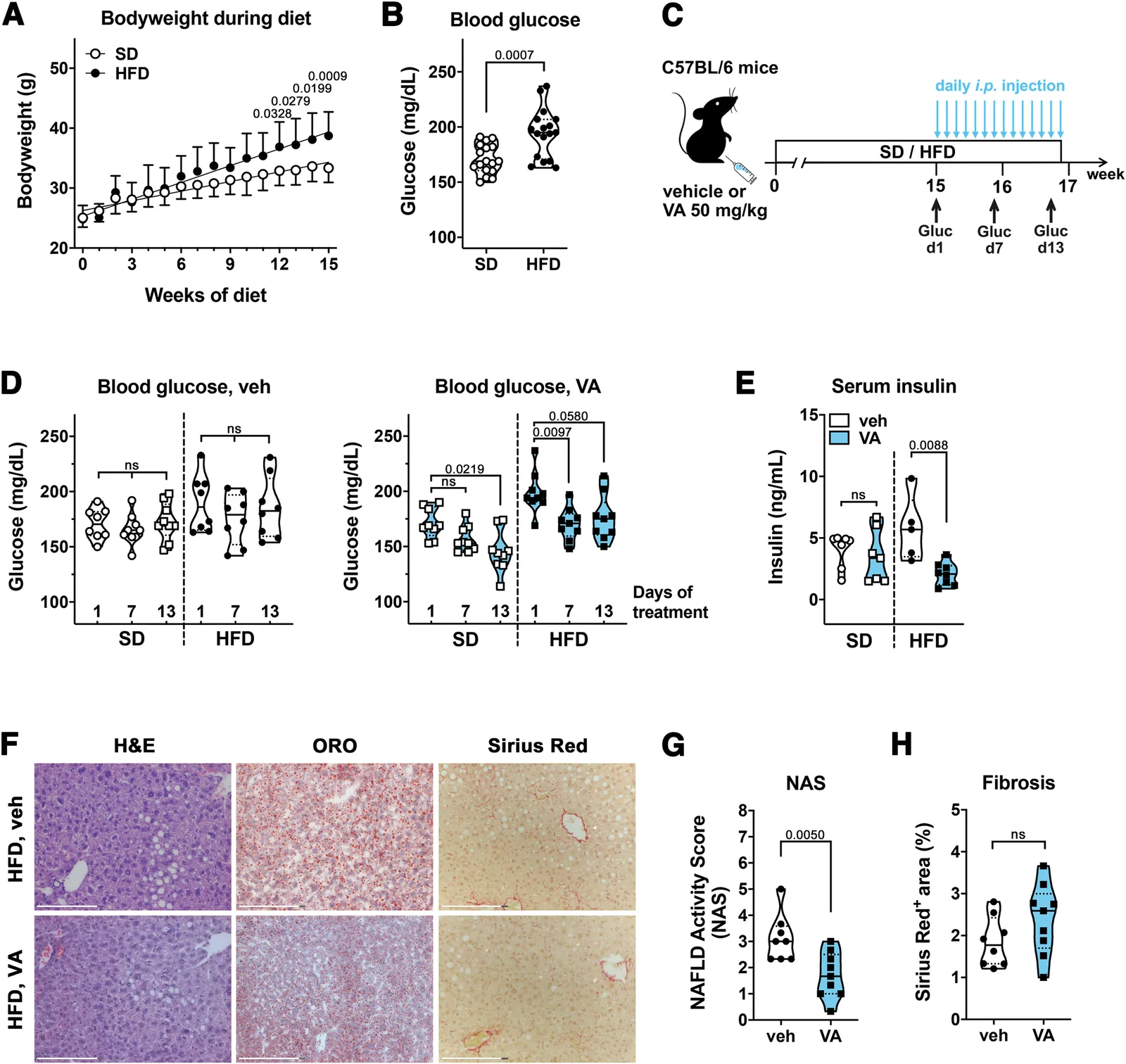
VA improves glucose homeostasis and hepatic steatosis. (**A**) Bodyweight increase during the 15 weeks of high fat diet (HFD) or standard diet (SD). Values represent mean ± standard deviation of SD- (*n* = 18) and HFD-fed (*n* = 17) mice. Data were analyzed using a repeated measures mixed-effects model with Sidak’s multiple comparisons test. (**B**) Blood glucose levels in SD- and HFD-fed mice at the end of the diet. Dots represent individual mice (SD: *n* = 17; HFD: *n* = 17). Unpaired two-tailed *t* test. (**C**) Scheme of the experimental protocol. After 15 weeks of diet, mice received a daily i.p. injection for 2 weeks of a mixture of *cis*- and *trans*-VA or vehicle control (veh). Blood glucose (Gluc) values were assessed right before the first injection (day 1), and after 7 and 13 days of treatment. (**D**) Blood glucose levels in SD- and HFD-fed mice. The left panel shows values in vehicle-treated mice, the right panel in VA-treated mice (veh: SD day 1 *n* = 8; SD day 7 and 13 *n* = 9; HFD day 1, 7 and 13 *n* = 8; VA: *n* = 9 for all conditions). One-way ANOVA analysis with Tukey´s correction for multiple comparisons. ns: not significant, vehicle: SD: day 1 *vs* day 7: *P* = 0.995, day 1 *vs* day 13: *P* > 0.999; HFD: day 1 *vs* day 7: *P* = 0.777, day 1 *vs* day 13: *P* = 0.999. VA: SD: day 1 *vs* day 7: *P* = 0.463. (**E**) Serum insulin levels measured at the end of the experiment. Two-way ANOVA analysis with Sidak´s correction for multiple comparisons (SD: veh *n* = 7, VA *n* = 7; HFD: veh *n* = 5, VA *n* = 8; ns: not significant, *P* = 0.996. (**F**) Representative liver sections from HFD-fed mice treated with VA or veh, and analyzed by hematoxylin–eosin (H&E), Oil Red O (ORO) or Sirius Red staining, to visualize tissue structure, lipid accumulation and collagen deposition, respectively. Scale bars represent 150 µm. (**G**, **H**) NAFLD Activity Score (NAS) (**G**) and fibrosis (**H**) in HFD-fed mice, calculated from H&E-stained and Sirius Red-stained liver sections, respectively. Unpaired two-tailed *t* test (veh *n* = 8, VA *n* = 9), ns: not significant (*P* = 0.1538). Source data are available online for this figure.

**Figure EV6:**
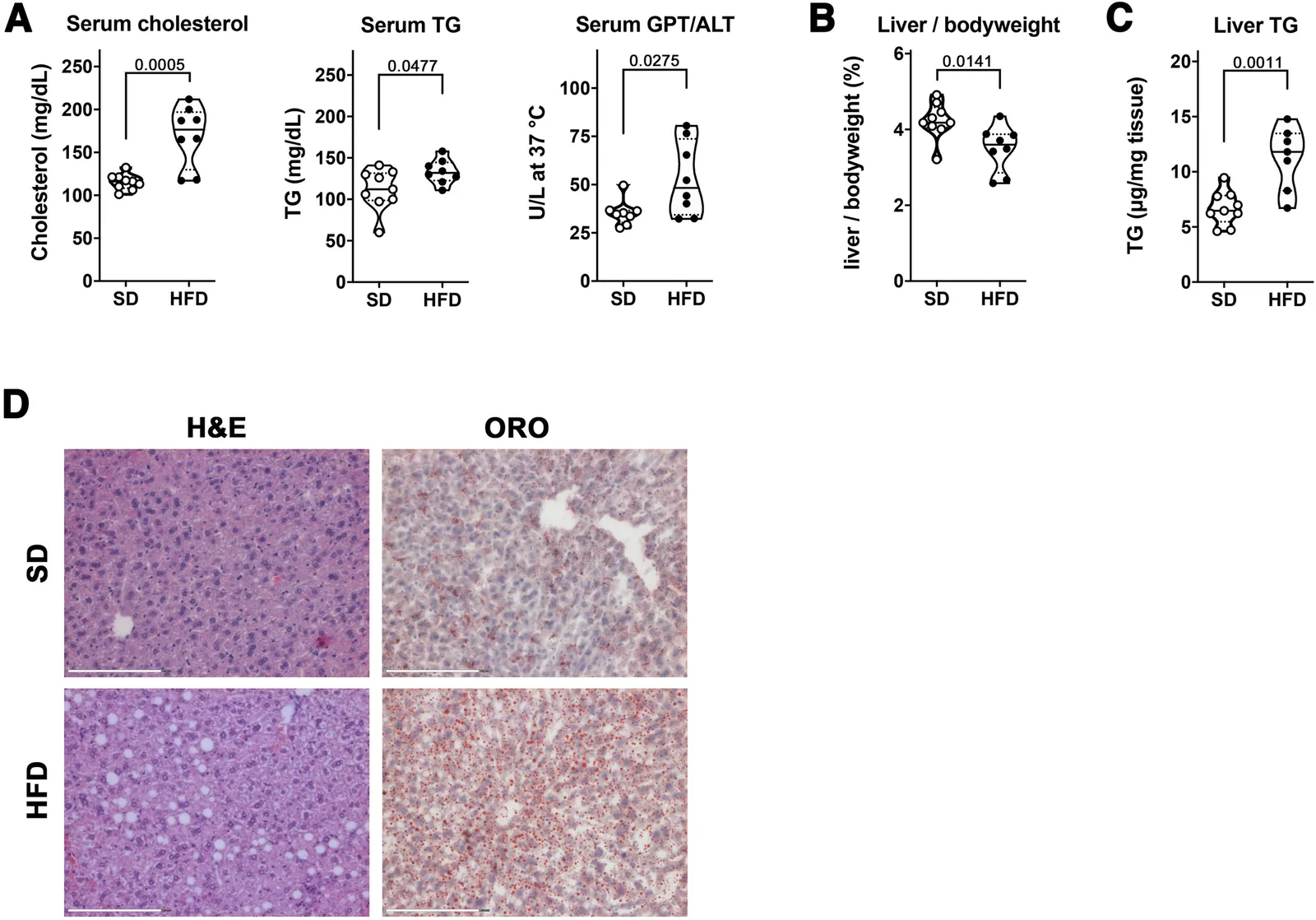
(related to Fig. 5): Comparison of SD- and HFD-fed mice. (**A**) Serum cholesterol, triglyceride (TG) and glutamic pyruvic transaminase/alanine transaminase (GPT/ALT) values in male C57BL/6 mice fed either with standard diet (SD) or a high-fat diet (HFD) for 15 weeks. Each dot represents an individual mouse. Unpaired two-tailed *t* test, SD: *n* = 8–9, HFD: *n* = 8. (**B**) Percentage of liver weight normalized by body weight. Unpaired two-tailed *t* test, SD: *n* = 9, HFD: *n* = 8. (**C**) Total liver TG. Unpaired two-tailed *t* test, SD: *n* = 9, HFD: *n* = 7. (**D**) Representative hematoxylin-eosin (H&E)- and Oil Red O (ORO)-stained liver sections from SD- or HFD-fed mice at the end of the 15-week diet. Scale bars represent 150 µm.

To evaluate the potential benefits of VA-induced LRH-1 activation in metabolic disorders, we employed a mouse model of diet-induced obesity and hyperglycemia (diet-induced obese (DIO) mice), as LRH-1 has been previously shown to regulate glucose and lipid metabolism (Lee et al, 2011). Mice were randomly assigned to receive either a high-fat diet (HFD) or a standard diet (SD) for 15 weeks. By week 12, the HFD group exhibited significantly higher body weight (Fig. 5A), along with elevated blood glucose (Fig. 5B), serum cholesterol, triglycerides (TG), and the hepatic transaminase GPT/ALT (Fig. EV6A). Additionally, normalized liver weight was altered (Fig. EV6B), hepatic TG content was increased (Fig. EV6C), and histological analysis revealed a fatty liver with enhanced lipid deposition (Fig. EV6D) at the experimental endpoint.

**Figure EV7:**
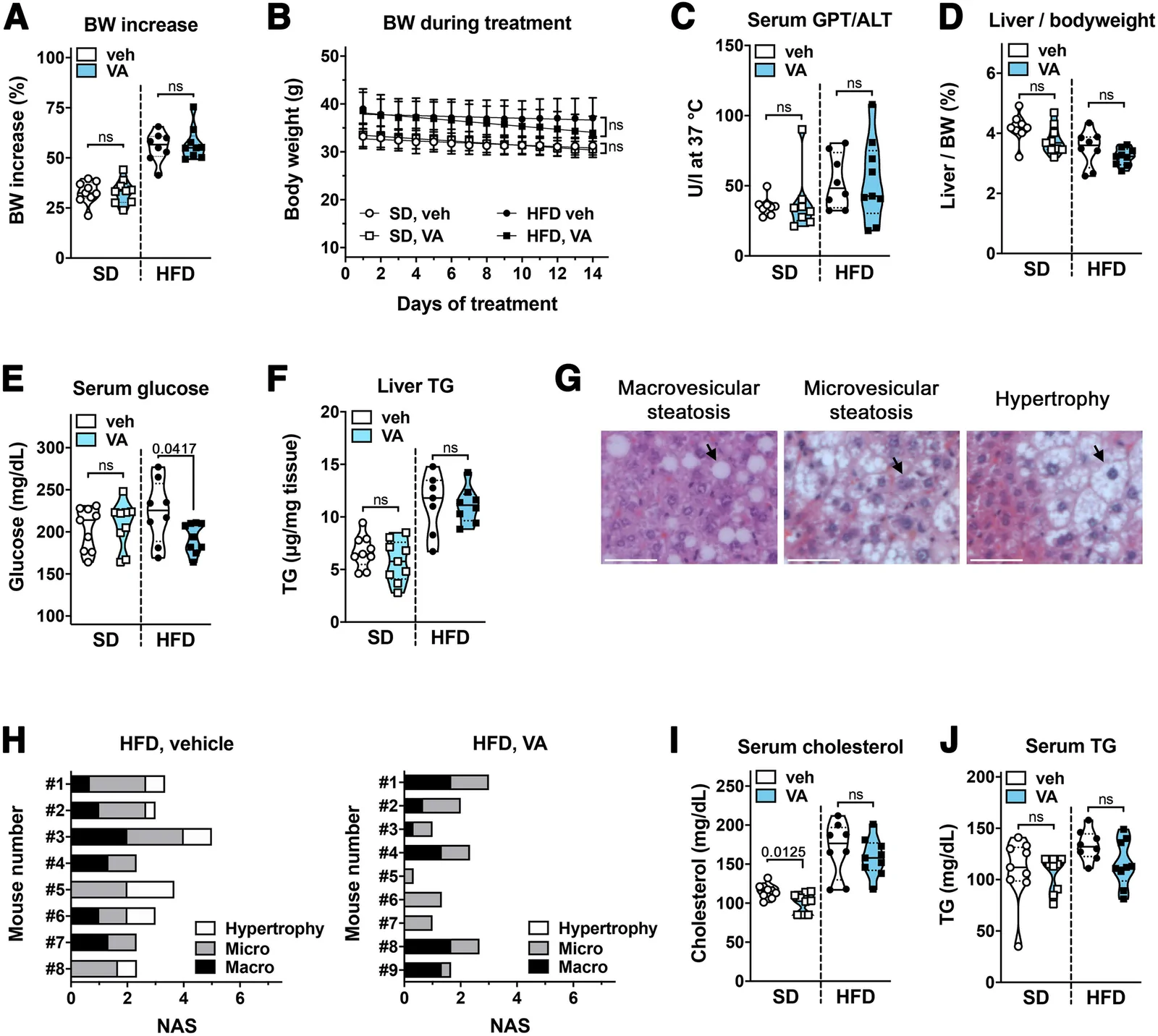
(related to Fig. 5): Effects of VA administration in SD- or HFD-fed mice. (**A**) Comparison of the bodyweight (BW) increase at the end of the SD or HFD between vehicle (veh)- and VA-treated mice. Unpaired two-tailed *t* tests, SD: veh *n* = 9, VA *n* = 9; HFD: veh *n* = 8, VA *n* = 9. (**B**) BW changes during the 2 weeks of treatment with vehicle control or VA. Values represent mean ± SD of *n* = 18 (SD) and *n* = 17 (HFD) mice. Mixed-effects analysis with Sidak’s multiple comparisons test; ns: not significant. (**C**–**F**) Serum GPT/ALT levels (**C**), normalized liver weights (**D**), serum glucose (**E**) and liver triglycerides (TG) (**F**) after vehicle or VA treatment in SD- or HFD-fed mice. Unpaired two-tailed *t* tests, ns: not significant, SD: veh *n* = 8–9, VA *n* = 9; HFD: veh *n* = 8, VA *n* = 9. (**G**) Enlargements of H&E-stained liver sections to highlight different aspects of steatosis. Macrovesicular steatosis is characterized by hepatocytes containing a single large fat droplet or multiple smaller, well-defined droplets, which expand in the cell and displace the nucleus to the periphery. Microvesicular steatosis is defined by hepatocytes with distended, foamy cytoplasm and small lipid vesicles. Hypertrophy defines enlarged hepatocytes compared to neighboring normal hepatocytes, with an increase in their cytoplasmic volume. Scale bars represent 50 µm. Arrows indicate representative cells. (**H**) NAFLD activity score (NAS) in individual mice with relative contribution of macrovesicular steatosis, microvesicular steatosis and hepatocyte hypertrophy, as analyzed in H&E-stained liver sections. (**I**, **J**) Serum cholesterol (**I**) and TG (**J**) levels after vehicle or VA treatment in SD- or HFD-fed mice. Unpaired two-tailed *t* tests, ns: not significant, SD: veh *n* = 9, VA *n* = 9; HFD: veh *n* = 8, VA *n* = 9.

After 15 weeks, mice were randomly assigned to receive daily i.p. injections of VA (50 mg/kg) or vehicle for 2 weeks, while maintaining their respective diets. VA treatment had neither a significant effect on body weight or weight gain during treatment (Fig. EV7A,B), nor did it induce hepatic toxic effects, as both serum GPT/ALT levels and normalized liver weights remained comparable to controls (Fig. EV7C,D). Blood glucose was measured via tail vein puncture right before the first i.p. injection (day 1) and after 7 and 13 days of treatment (Fig. 5C). VA administration reduced blood glucose in the HFD group at both day 7 and day 13 of treatment (Fig. 5D), with the latter approaching statistical significance (*P* = 0.058). A similar trend was observed in the SD group at day 13, whereas no change in glucose levels was observed in either group after vehicle administration. Serum glucose levels assessed at the end of the experiment were also lower in VA-treated HFD mice (Fig. EV7E), consistent with the tail vein blood measurements. VA also decreased serum insulin levels in the HFD mice, but not in the SD group (Fig. 5E).

LRH-1 plays a central role in liver lipid homeostasis by coordinating several metabolic pathways. Accordingly, disturbances in these LRH-1-regulated pathways result in the development of metabolic disorders, such as NAFLD and type 2 diabetes (Sun et al, 2021). Given that activation of LRH-1 by DLPC protected mice from hepatic steatosis (Lee et al, 2011), we examined the impact of VA administration on liver lipid accumulation in HFD-fed mice. Histological analysis of H&E- and Oil Red O (ORO)-stained liver sections revealed a mild reduction in lipid deposition following VA treatment (Fig. 5F), although no significant change in hepatic TG levels was detected (Fig. EV7F). We performed a more detailed analysis by quantifying macro- and microvesicular steatosis and hepatocyte hypertrophy separately (Fig. EV7G) and calculated the NAFLD activity score (NAS) (Liang et al, 2014). VA administration significantly improved the NAS value (Fig. 5G), predominantly by reducing the contribution of microvesicular steatosis and hepatocyte hypertrophy in comparison to vehicle-treated mice (Fig. EV7H). No effects were observed on collagen deposition and fibrosis, assessed by Sirius Red staining (Fig. 5H), or on serum cholesterol and TG levels (Fig. EV7I,J).

### VA decreases the expression of lipogenic genes in the liver

**Figure 6:**
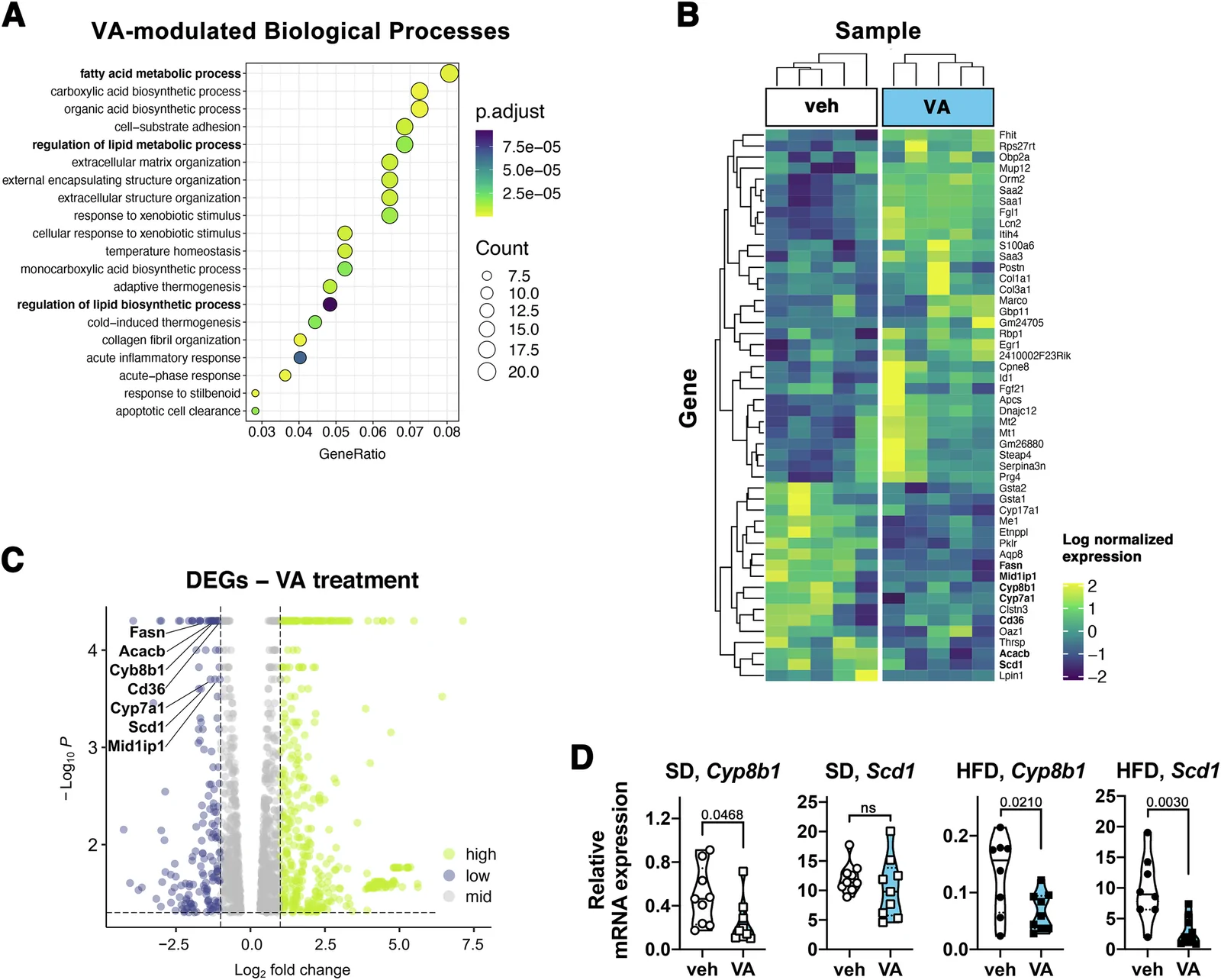
VA modulates hepatic lipid metabolic pathways and inhibits de novo lipogenesis. (**A**, **B**) Top 20 enriched gene ontologies (GO) (**A**) and heatmap of the top 50 differentially expressed genes (DEGs, both up- and downregulated) (**B**), identified from bulk RNA sequencing of liver samples from HFD mice treated with either vehicle control (veh, *n* = 5) or VA (*n* = 5). GO enrichment was performed using a set of significantly up- and downregulated genes, reflecting biological processes modulated by VA treatment. (**C**) Volcano plot of top VA-induced DEGs (same dataset shown as in (**A**, **B**)), highlighting genes involved in de novo lipogenesis and lipid biosynthesis processes. Differential expression was assessed using the DESeq2 Wald test with Benjamini–Hochberg correction for multiple testing. Thresholds for significance were set at adjusted *P* value < 0.05 and log_2_ fold change (FC) of ±1 (low: log_2_ FC < −1, mid: −1< log_2_FC < 1, high: log_2_FC > 1). (**D**) Validation of selected DEGs by RT-qPCR analysis in liver tissue samples from the same mice. Dots represent individual mice (SD: veh *n* = 9, VA *n* = 8; HFD: veh *n* = 8, VA *n* = 9). Unpaired two-tailed *t* test. ns: not significant, *P* = 0.3608. Source data are available online for this figure.

**Figure EV8:**
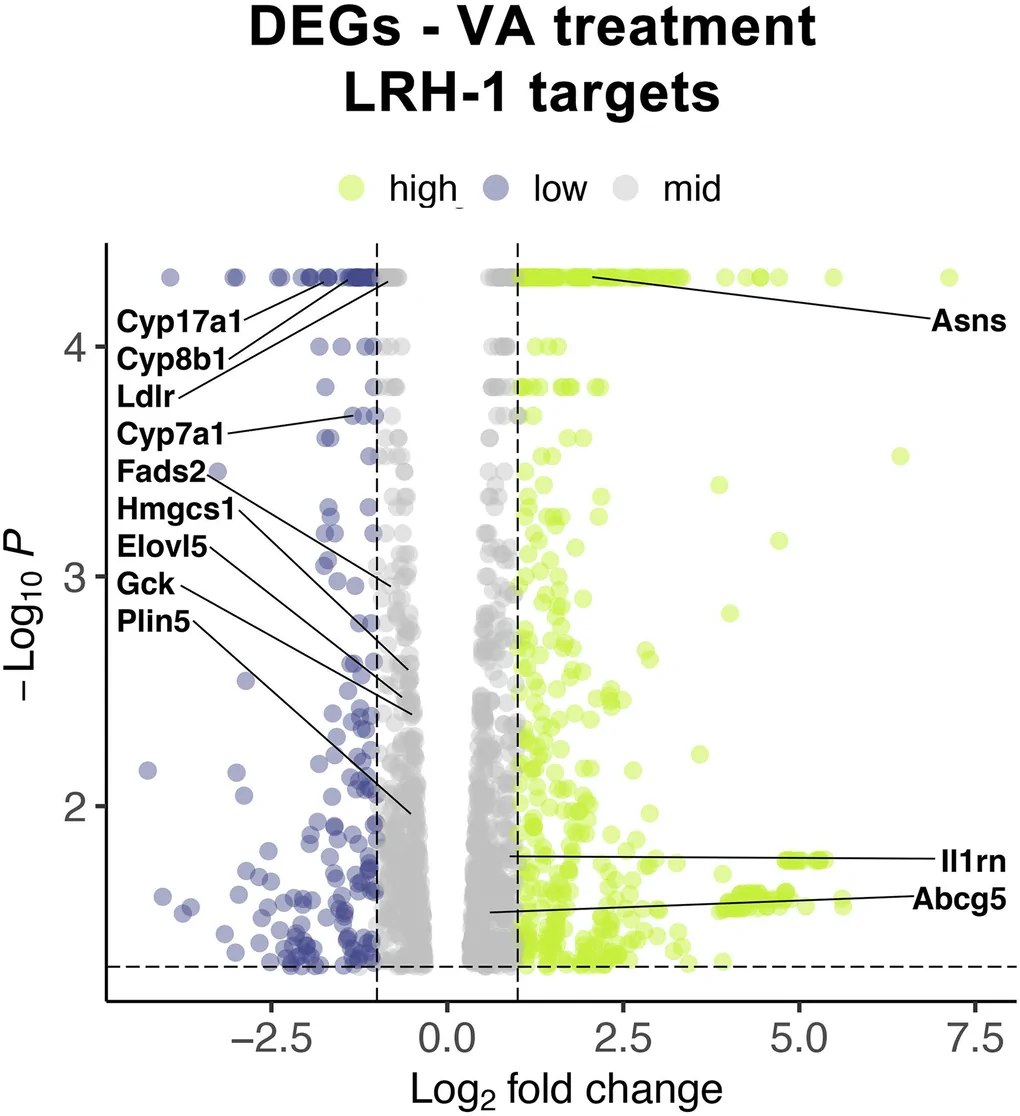
(related to Fig. 6): VA modulates expression of different LRH-1 target genes. Volcano plot of the differentially expressed genes (DEGs) in VA-treated HFD-fed mice compared to vehicle-treated mice. Annotated genes indicate established LRH-1 targets and are listed in Table EV1. Thresholds for significance were set at adjusted *P* value < 0.05 and log2 fold change of ±1 (low: *P* adj < −1, mid: −1<*P* adj<1, high: *P* adj>1).

To explore the molecular mechanisms underlying the improvements in glucose and lipid homeostasis induced by VA, we performed RNA-sequencing analyses on the liver samples from VA- or vehicle-treated HFD mice. Gene ontology analysis of significantly differentially expressed genes revealed that VA treatment modulates pathways related to lipid biosynthesis and fatty acid metabolism (Fig. 6A), alongside altered expression of LRH-1 target genes (Fig EV8 and Table EV1). Notably, VA treatment suppressed expression of key de novo lipogenesis genes, including *Acacb*, *Fasn*, and *Scd1* (Fig. 6B,C). These findings align with previous studies showing downregulation of lipogenic genes following LRH-1 activation with DLPC or with the synthetic agonists 10CA and 6N (Lee et al, 2011; Mays et al, 2022; Cato et al, 2022). Additionally, the expression of *Mid1ip1*, a gene involved in the positive regulation of fatty acid biosynthesis via the nuclear receptor LXR (Izumi et al, 2021; Inoue et al, 2011), and *Cd36*, which encodes a fatty acid transporter, was also downregulated in VA-treated mice. Interestingly, VA treatment also decreased expression of the LRH-1 targets *Cyp7a1* and *Cyp8b1*. RT-qPCR analysis of *Cyp8b1* and *Scd1* confirmed their downregulation upon VA treatment (Fig. 6D). Notably, while the downregulation of *Scd1* was restricted to the context of the HFD, a reduction in *Cyp8b1* expression was also detected in SD-fed mice. Overall, these results suggest that a reduction in lipogenic flux underlies the beneficial effects of VA on LRH-1-regulated glucose and lipid homeostasis.

## Discussion

It is increasingly appreciated that the gut microbiota plays a central role in the modulation of host physiological processes, with different bacteria having health-promoting effects. At least in part these effects may be mediated by soluble factors activating NR-regulated processes in the host. One example is provided by Bifidobacteria, Gram-positive bacteria commonly found in the human oral cavity, gastrointestinal tract and dairy products (Hidalgo-Cantabrana et al, 2017). Among these, the strain B420 of *Bifidobacterium animalis* subsp. *lactis* has been shown to provide various health benefits (reviewed in (Uusitupa et al, 2020)), including enhancement of mucosal integrity (Putaala et al, 2008; Mokkala et al, 2016; Zhang et al, 2020), improvement of glycemic control (Stenman et al, 2014), and increased host resistance to pathogens (Amar et al, 2011) in different preclinical studies. Furthermore, B420 administration in mice mitigated liver damage in the context of autoimmune hepatitis (Zhang et al, 2020). A clinical trial showed that B420 supplementation reduced body fat mass, waist circumference, energy intake and body weight compared to placebo in overweight or obese individuals (Stenman et al, 2016). These effects were later associated with changes in the microbiota composition, fecal metabolites and plasma bile acid levels (Hibberd et al, 2019). Interestingly, B420 did not colonize the host gut microbiome after ten consecutive days of administration (Lopez-Pier et al, 2021), which may suggest that the beneficial effect could rely on transiently produced metabolites. Indeed, many bacteria-derived metabolites, including short-chain fatty acids, BA and vitamins, are known to promote host health, in part by directly modulating NR activity.

In our study, we provide evidence that the NR LRH-1 can also be regulated by bacterial metabolites, and we describe VA for the first time as an LRH-1 ligand. VA was able to stimulate LRH-1 activity, and its effect in vivo on the regulation of metabolism correlated well with the beneficial effects previously described for known LRH-1 ligands (Lee et al, 2011; Cato et al, 2022). Thus, this study identifies VA as an LRH-1 ligand and health-promoting component in B420 probiotic bacteria.

Targeting LRH-1 with agonistic and antagonistic ligands is an attractive therapeutic approach. Indeed, the small molecule LRH-1 agonist BL001 could prevent autoimmune diabetes development in mouse models of type 1 diabetes, and promote human beta cell survival and function (Cobo-Vuilleumier et al, 2018a, 2018b). Also, the LRH-1 agonist DLPC improved glucose homeostasis, and reduced hepatic lipid accumulation in mouse models of insulin resistance (Lee et al, 2011). The discovery that DLPC interacts with amino acid residues located at the mouth of the LRH-1 LBP (Musille et al, 2012) encouraged the development of PL-based ligands that combine these interactions at the mouth of the LBP with those deep within the pocket, characteristic of RJW100-based ligands. Indeed, the hybrid molecules 6 N, with a sulfamide group at position R^1^ (Mays et al, 2019), and 10CA, where a carboxylic acid tail was added at position R^4^ (Flynn et al, 2018), act as potent LRH-1 ligands and induce expression of LRH-1-regulated anti-inflammatory genes, thereby promoting amelioration of colitis (Flynn et al, 2018; Mays et al, 2022).

Ligands that inhibit LRH-1 activity also have promising therapeutic applications, for example, in cancer, where LRH-1 acts as an oncogene. We successfully employed the LRH-1 inhibitor 3d2 to inhibit proliferation and sensitize T-ALL to GC treatment (Michalek et al, 2022). Interestingly, intestinal GC synthesis, known to be regulated by LRH-1 (Mueller et al, 2006; Coste et al, 2007), as well as blood glucose and insulin levels have already been associated with alterations of the intestinal microbiome in a different context (Mukherji et al, 2013).

LRH-1 has been shown to interact with and/or to be modulated by a variety of structurally different compounds. Examples include the herbicide atrazine (Suzawa and Ingraham, 2008), unconjugated bilirubin (Chapagain et al, 2024) or ivermectin-related compounds, like abamectin and doramectin (Malabanan et al, 2023). Thus, a scenario with no strict binding rules for LRH-1 ligands emerges, where likely multiple interaction possibilities exist within and in proximity of the LBP of LRH-1, that are able to facilitate binding of co-regulators. With VA we identified a novel bacteria-derived molecule that possibly engages key interactions within the LRH-1 LBP, with its carboxylic acid group and/or the acyl chain, similar as described for PL ligands. Interestingly, the position of the double bond appears to be an important factor determining binding to and transactivation of LRH-1. In our study, fatty acids of equal length (C18), but different positions of the double bond than VA, had lower binding activities and failed to activate LRH-1. However, the possibility that these compounds are metabolized or unstable in vitro and in vivo cannot be excluded. Also, whether VA effects in vivo are a consequence solely of direct LRH-1 activation or could involve induced biosynthesis of alternative LRH-1 ligands remains to be investigated. Interestingly, although a tenfold higher K_d_ was estimated for *trans*-VA compared to *cis*-VA, both displayed similar LRH-1-activating capacity in cell-based functional studies. In the cell, multiple determinants of the final functional output, including signal amplification via interaction with cofactors or partners, metabolic conversion to effectors shared with other NRs, potential nonlinear or saturated functional response or improved bioavailability may account for these differences.

VA is also detected in human tissue and plasma, and seems to accumulate primarily due to the consumption of meat and dairy products. Notably, physiological concentrations of *trans*-VA in human blood are reported to be in the µM range, as documented in the Human Metabolome Database (https://hmdb.ca/). Bacteria are also a source of VA, as we have found VA in different bacterial species. Thus, likely cows incorporate bacteria-derived VA in their milk and meat, and upon consumption VA ends up in human tissues.

Interestingly, VA-induced health benefits correlate with those already shown for the LRH-1 ligands DLPC and 10CA (Lee et al, 2011; Mays et al, 2022). Likewise, dietary supplementation of VA in animal models was shown to improve hepatic steatosis, and decrease serum glucose and insulin (Jacome-Sosa et al, 2014). The authors suggested that VA can improve whole-body insulin resistance, although another study reported that long-term feeding of beef fat enriched in *trans*-VA did not improve glucose homeostasis (Xu et al, 2024).

Remarkably, a human study investigating the association of fatty acids in the de novo lipogenesis pathway with metabolic risk factors and development of type 2 diabetes revealed that VA was associated with lower diabetic risk (Wenjie et al, 2015). Indeed, effects of VA on expression of *Scd1*, coding for the rate-limiting enzyme in this pathway, have already been reported (Minville-Walz et al, 2012). We also found downregulation of *Scd1*, together with *Acacb* and *Fasn*, as one of the major gene expression changes induced by VA treatment, that correlate well with the metabolic benefits observed in our mouse model of metabolic disorders. These data are also in agreement with the downregulation of lipogenic genes following LRH-1 activation with DLPC or with 10CA and 6 N (Mays et al, 2022; Cato et al, 2022; Lee et al, 2011). In addition, VA-induced alterations of expression of the LRH-1 targets *Fads2* and *Elovl5* correlate with altered expression of these genes in LRH-1-deficient hepatocytes (Miranda et al, 2018).

In conclusion, we show here that LRH-1 activity can be modulated by the bacteria-derived, exogenous metabolite VA. This not only establishes VA as a novel LRH-1 ligand, but also opens the perspective that LRH-1, similarly to other NRs (Keely, 2017), mediates communication between the gut bacteria and the host, allowing microbiota-derived dynamic regulation of host physiological and metabolic processes, in homeostasis and disease. In this context, modulation of the host microbiota composition may represent a new therapeutic approach in the treatment of LRH-1-regulated disorders.

## Methods

**Table:** Reagents and tools table

Reagent/resource	Reference or source	Identifier or catalog number
**Experimental models**		
Male C57BL/6 mice (*M. musculus*), 8–12 weeks old	Originally obtained from Jackson Laboratory	Strain # 000664 RRID:IMSR_JAX:000664
LRH-1 VilCre and LRH-1 L2L2 mice (*M. musculus*), 8–12 weeks old	Previously described in Coste et al, 2007	N/A
Caco-2 (*H. sapiens*)	ATCC	Cat# HTB-37
HEK 293 T (*H. sapiens*)	ATCC	Cat# CRL-3216; RRID:CVCL_0063
LS174T (*H. sapiens*)	ATCC	Cat# CL-188
LS174T human Flag-tagged LRH-1, 4-HT-inducible	This study	N/A
LS174T human Flag-tagged LRH-1 F342W/I416W, 4-HT-inducible	This study	N/A
LS174T murine LRH-1, 4-HT-inducible	This study	N/A
LS174T EGFP, 4-HT-inducible	This study	N/A
*Bifidobacterium animalis* subsp. *lactis* 420 (B420^TM^)	Danisco Deutschland GmbH	Sample #767, Batch 1102133655
*Escherichia coli* BL21	Previous hosting lab	N/A
*Escherichia coli* Nissle 1917 (EcN) (Mutaflor)	Ardeypharm	N/A
*Escherichia coli* Rosetta^TM^ 2 (DE3)	Novagen	Cat# 71400
*Enterobacter cloacae*	DSMZ	DSM No.: 30054
*Enterococcus faecalis*	DSMZ	DSM No.: 20478
*Klebsiella pneumoniae*	DSMZ	DSM No.: 681
*Pseudomonas aeruginosa* PA01	DSMZ	DSM No.: 22644
*Proteus mirabilis*	DSMZ	DSM No.: 4479
Symbioflor2	Symbiopharm	N/A
*Vibrio cholerae*	DSMZ	DSM No.: 106276
**Recombinant DNA**		
Plasmids		Table 2
**Antibodies**		
Mouse mAB anti-Flag M2	Sigma-Aldrich	Cat# F1804
Mouse mAB anti-GFP	Roche	Cat# 11814460001
Mouse mAB anti-Tubulin	Sigma-Aldrich	Cat# T5168; RRID:AB_477579
Rabbit pAB anti-NR5A2	Sigma-Aldrich	Cat# HPA005455
**Oligonucleotides and other sequence-based reagents**		
Primers		Table 1
**Chemicals, enzymes and other reagents**		
3-(4,5-dimethylthiazol-2-yl)-2,5-diphenyl-tetrazolium bromide (MTT)	Sigma-Aldrich	Cat# M2128
4-hydroxytamoxifen	Sigma-Aldrich	Cat# H7904
Adenosine 5-triphosphate (ATP)	Carl Roth	Cat# K029.2
ammonium thiocyanate	Sigma-Aldrich	Cat# 1012130500
AMPure XP	Beckman Coulter	Cat# A63881
*Cis*-vaccenic acid	Sigma-Aldrich	Cat# V0384
*Cis*-vaccenic acid	Cayman chemical	Cat# 20023
Co-Enzyme A	PJK	Cat# 102211
cOmplete TM, EDTA-free Protease Inhibitor Cocktail	Roche	Cat# 04693132001
Compound 3d2 (3d2)	ChemBridge Corp	ID: 16728690
Direct Red 80	Sigma-Aldrich	Cat# 365548
D-Luciferine Na salt (Luciferin)	PJK	Cat# 102132
DNase I (RNase-free)	New England Biolabs	Cat# M0303
Dulbecco’s Modified Eagle’s Medium	Sigma-Aldrich	Cat# D5796
Fetal Bovine Serum	Sigma-Aldrich	Cat# F7524
Gentamicin	Sigma-Aldrich	Cat # G1272
Hygromycin B	InvivoGen	Cat# ant-hg-1
Iscove’s Modified Dulbecco’s Medium	Sigma-Aldrich	Cat# I3390
Isopropyl β-D-1-thiogalactopyranoside (IPTG)	Carl Roth	Cat# 2316.3
JetPei^TM^	Polyplus Transfection	Cat# 101000020
L-glutamine	Sigma-Aldrich	Cat# G7513
Luminol	Fluka	Cat# 09253
Oil Red O	Sigma-Aldrich	Cat# O0625
O-nitrophenyl-b-D-galactopyranoside (ONPG)	Carl Roth	Cat# CN.22
P-coumaric acid	Sigma-Aldrich	Cat# C-9008
PeqGOLD TriFastTM	VWR	Cat# 30–2030
Puromycin. 2HCl	Enzo Life Sciences	Cat# BML-GR312
SR1848	Sigma-Aldrich	Cat# SML 1513
TEV-protease	Sigma-Aldrich	Cat# T4455
Tissue-Tek O.C.T Compound	Sakura	Cat# 4583
*Trans*- vaccenic acid	Sigma-Aldrich	Cat# V1131
TRIzolTM Reagent	Invitrogen	Cat# 15596026
**Software**		
Bioanalyzer Smear Analysis tool	Agilent Technologies	N/A
MO.Affinity Analysis software	NanoTemper Technologies	version 2.2.4
NextSeq System Suite	Illumina Inc	v2.2.0
Prism	GraphPad Software	V7.0
QuantStudio Design & Analysis Software	Applied Biosystems	V1.5.2
R package “EnhancedVolcano”		version 1.18.0
R package clusterProfiler		version 4.8.3
R package DESeq2		version 1.40.2
R package pheatmap		version 1.0.12
ZEN Software	Carl Zeiss Microscopy	V2.5 (blue edition)
**Other**		
10 kDA Amicon Ultra tube	Millipore	Cat# UFC901024
High-Capacity cDNA Reverse Transcription Kit	Applied Biosystems	Cat# 4368814
High-fat diet with 45% calories from fat	Safe lab	SAFE 245HF, D12451
HiLoad 16/60 Superdex^®^ 200 PG column	Cytiva	Cat# 28-9893-35
HisTrap HP column	Cytiva	Cat# 29-0510-21
Monolith NT Protein Labeling Kit GREEN-NHS	NanoTemper Technologies	Cat# MO-L002
Monolith NT.115 capillaries	NanoTemper Technologies	Cat# MO-K022
PD SpinTrap G-25 column	Cytiva	Cat# 28-9180-04
Pierce™ BCA Assay Kit	Thermo Fisher	Cat# 23225
PVDF membrane	Roche	Cat# 03010040001
QuikChange II XL Site-Directed Mutagenesis Kit	Agilent Technologies	Cat# 200521
Standard diet	Safe lab	SAFE A03
SV Total RNA Isolation System	Promega	Cat# Z3100
SYBR® Green	Applied Biosystems	Cat# 4385614
Triglyceride Quantification Kit	Sigma-Aldrich	Cat# MAK266
TruSeq® Stranded mRNA Sample Preparation Kit	Illumina Inc	Cat# 20020594
Ultra Sensitive Mouse Insulin ELISA Kit	CrystalChem	Cat# 90080
Venor®GeM Classic	Minerva Biolabs	Cat# 11-1050
3.5 KDa Slide-A-Lyzer MINI Dialysis Device	ThermoFisher Scientific	Cat# 88403
Bioanalyzer 2100	Agilent Technologies	N/A
BioAnalyzer 4200 tapeStation	Agilent Technologies	N/A
Glucometer	GlucoCheck	N/A
Hydrax C500 cryostat	Carl Zeiss	N/A
Image Quant LAS4000	GE Healthcare	N/A
Infinite® 200 PRO plate reader	TECAN	N/A
Monolith NT.115 instrument	NanoTemper Technologies	N/A
NextSeq500 System	Illumina Inc	N/A
Reflotron Plus	Roche	N/A
QuantStudio 3 Real-Time-PCR-System	Applied Biosystems	N/A
Zeiss Observer Z.1 microscope	Carl Zeiss	N/A

### Animal studies

All animal experiments were conducted according to the animal experimentation regulations of Germany and were approved by the Ethics Review Committee of the Regional Council Freiburg (reference number: 35-9185.81/G-17/91). Mice were bred and housed under specific pathogen-free conditions in individually ventilated cages at the animal facility of the University of Konstanz and were acclimated to a 12 h light/dark cycle with free access to food and water. Male C57BL/6 mice (originally obtained from Jackson Laboratory) aged 8–12 weeks were randomly assigned to groups fed either a standard diet (SD, SAFE A03, standard diet) or a high-fat diet with 45% calories from fat (HFD, SAFE 245HF, D12451) for 15 weeks. A mixture of *cis*- and *trans*-VA (1:1) dissolved in 40% PEG-400, 2% Tween-80 in PBS or the vehicle was administered daily to mice at 50 mg/kg by intraperitoneal (i.p.) injection (volume = 150 μl/mouse) for 2 weeks, while continuing the previously assigned diet. Blood glucose values were assessed right before the first i.p. injection (day 1) and after 7 and 13 days of treatment with a glucometer (GlucoCheck) by puncturing the tail vein. Mice were sacrificed 4 h after the last treatment (day 14) by CO_2_ inhalation. Blood and tissues (liver, small intestine) were immediately harvested. Part of the tissues were snap-frozen in liquid nitrogen for RNA isolation, cholesterol and triglyceride (TG) analysis or formalin-fixed for paraffin sections. In parallel, tissue was embedded in Tissue-Tek O.C.T. Compound (Sakura) to prepare cryosections. Mice exhibiting a body weight gain of less than 45%, or greater than 75%, relative to their initial value were excluded from subsequent analyses.

Intestinal epithelial cell-specific LRH-1 knockout mice (LRH-1 VilCre) and the isolation of intestinal crypts have been described previously (Noti et al, 2010; Coste et al, 2007; Ahmed et al, 2023).

### Serum analysis

After collection, blood was allowed to clot at RT for 30 min and was then centrifuged for 15 min at 13,000×*g*. Serum was collected and glucose, cholesterol, TG and ALT/GTP values were immediately analyzed with a Reflotron instrument (Roche). An aliquot was stored at −20 °C for determination of insulin content with the Ultra Sensitive Mouse Insulin ELISA Kit (CrystalChem) following the manufacturer´s instructions.

### Histological analysis of liver sections

Tissue biopsies were fixed in formalin and embedded in paraffin. Paraffin sections of 4 µm were stained with hematoxylin and eosin (H&E) and evaluated blindly by light microscopy. Macrovesicular and microvesicular steatosis as well as hepatocellular hypertrophy were scored separately based on the percentage of the total area affected (0: area affected <5%, 1: 5-33%, 2: 34-66%, 3: >66%). Three images/mouse were inspected and the NAFLD Activity Score (NAS) was determined as the sum of the averaged scores, ranging from 0 to 9 (Liang et al, 2014).

### Analysis of liver fibrosis

Connective tissue and collagen deposition in the hepatic tissue was visualized by Picro Sirius Red staining. De-waxed and rehydrated paraffin liver tissue sections were stained using 0.1% (w/v) Direct Red 80 (Sigma-Aldrich) dissolved in an aqueous solution of saturated picric acid for 1 h and then washed with 0.5% acetic acid and water. Stained slides were dehydrated in three changes of 100% ethanol (3 min for each step), mounted and examined by light microscopy with a Zeiss Observer Z.1 microscope. Quantification of fibrosis was determined by measuring the percentage of red-stained area in three non-overlapping images per mouse based on thresholds using ImageJ software (Schindelin et al, 2012).

### Oil Red O staining

Lipid deposition in hepatic tissue was determined by Oil Red O staining (Mehlem et al, 2013). Frozen liver samples were cut with a Hydrax C500 cryostat (Zeiss) at −14 °C with a thickness of 10 μm, fixed in 10% formalin for 15 min and washed twice with water and once with 60% isopropanol for 5 min. Sections were stained with filtered Oil Red O solution (15 mL of 0.3% Oil Red O (Sigma) in pure isopropanol mixed with 10 mL H_2_O) for 5 min, and washed with water until clear. Hematoxylin counterstaining was performed for 1 min, then slides were washed, mounted and sealed. Images were taken with a Zeiss Observer Z.1 microscope.

### Liver triglyceride measurement

Lipid extraction from snap-frozen livers was adapted from Storlien et al and Frayn and Maycock (Frayn and Maycock, 1980; Storlien et al, 1991). Samples (50 mg) were mixed with 1 mL of chloroform/methanol (2:1, vol/vol) and homogenized using a TissueLyser (Qiagen) for 1 min. The mixture was then incubated on a rotator for 6–8 h. After incubation, 0.3 mL of double-distilled water was added, followed by vortexing and centrifugation at RT for 20 min at 2000 rpm. The lower organic phase was transferred to a new 2 mL tube and chloroform was evaporated overnight. A second extraction was performed by adding 0.5 mL of chloroform/methanol (2:1, vol/vol) to the remaining phases, followed by rotation and centrifugation. The samples from both the first and second extractions were combined and left overnight for complete evaporation. Samples were then vortexed in 200 μL of chloroform. Three microliters of the sample were pipetted in duplicate into a 96-well plate. Triglycerides (TG) were quantified using the Triglyceride Quantification Kit (MAK266, Sigma-Aldrich), following the manufacturer’s protocols.

### RNA isolation and quantitative real-time PCR analysis

Tissues (50–100 mg) were harvested and snap-frozen in liquid nitrogen. RNA isolation from adherent cells was performed 24 h after seeding 0.6–1.2 × 10^6^ cells on a six-well plate. Total RNA was isolated with peqGOLD TriFastTM (VWR) or TRIzolTM Reagent (Invitrogen) and subjected to DNase I treatment (New England Biolabs) (PeqLab), or isolated with the SV Total RNA Isolation System (Promega) according to the manufacturer’s instructions. The cDNA was generated from 1 to 2 µg RNA using a High-Capacity cDNA Reverse Transcription Kit (Applied Biosystems). Real-time quantitative PCR was performed using SYBR® Green (Applied Biosystems) with the StepOnePlusTM Real-time PCR System (Applied Biosystems). Gene expression was normalized using murine or human βActin or B2M reference genes, and represented as 2^−∆Ct^ (∆Ct = Ct target gene − Ct reference gene) or as fold change to untreated/mock-treated controls. Nucleotide sequences of primer sets used are indicated in Table 1. When possible, primers were designed to span an exon–exon junction.

**Table 1:** Sequence or source of primer pairs used in the study.

Product		Sequence (5’ → 3’)
**Primers for standard qPCR**		
Human β-actin	Forward	ATGGAGAAGGCTGGGGCTCA
	Reverse	TCTCCATGGTGGTGAAGACA
Human LRH-1	Forward	GGGCAACAAGTGGACTATTC
	Reverse	CCAGCTGGAAGTTTTCAAGG
Mouse β-actin	Forward	GATCAAGATCATTGCTCCTCCTGA
	Reverse	CAGCTCAGTAACAGTCCGCC
Mouse B2M	Forward	TTCTGGTGCTTGTCTCACTG
	Reverse	TATGTTCGGCTTCCCATTCT
Mouse Cyp7a1	Forward	CAGGGAGATGCTCTGTGTTCA
	Reverse	AGGCATACATCCCTTCCGTGA
Mouse Cyp8b1	Forward	CCCATAAGACGCCATCCCTC
	Reverse	AAGTGTGGGTGAGCCATCAG
Mouse Fgf15	Forward	GCCATCAAGGACGTCAGCA
	Reverse	CTTCCTCCGAGTAGCGAATCAG
Mouse Scd1	Forward	GAACACCCATCCCGAGAGTC
	Reverse	GAACTGGAGATCTCTTGGAGCA
Mouse Cyp11a1		QuantiTect Qiagen, #QT00161091
Mouse Cyp11b1		QuantiTect Qiagen, #QT01198575
**Primers for site-directed mutagenesis**		
F342W	Forward	CAAGCACGAAAAGCTGAGCACCTGGGGGCTTATGTGCAAAA
	Reverse	TTTTGCACATAAGCCCCCAGGTGCTCAGCTTTTCGTGCTTG
I416W	Forward	ACTGGGCAACAAGTGGACTATTCCATATGGGCATCACAAGCCGG
	Reverse	CCGGCTTGTGATGCCCATATGGAATAGTCCACTTGTTGCCCAGT

### RNA sequencing

RNA-Seq experiments were conducted at QIAGEN Genomic Services. The library preparation was done using TruSeq® Stranded mRNA Sample preparation kit (Illumina Inc). The starting material (500 ng) of total RNA was mRNA enriched using the oligodT bead system. The isolated mRNA was subsequently fragmented using enzymatic fragmentation. Then first strand synthesis and second strand synthesis were performed, and the double stranded cDNA was purified (AMPure XP, Beckman Coulter). The cDNA was end-repaired, 3’-adenylated, Illumina sequencing adaptors were ligated to the fragments ends and the library was purified (AMPure XP). The mRNA stranded libraries were pre-amplified by PCR and purified (AMPure XP). The libraries size distribution was validated and quality inspected on a Bioanalyzer 2100 or BioAnalyzer 4200 tapeStation (Agilent Technologies). High quality libraries were pooled in equimolar concentrations based on the Bioanalyzer Smear Analysis tool (Agilent Technologies). The library pool(s) were quantified using qPCR and the optimal concentration of the library pool were used to generate the clusters on the surface of a flowcell before sequencing on a NextSeq500 instrument (75 cycles) according to the manufacturer's instructions (Illumina Inc.). Differentially expressed genes (DEGs) were calculated with an adjusted *P* value < 0.05 using the R package DESeq2 (version 1.40.2), and GO analysis was performed with clusterProfiler (version 4.8.3). Visualization was performed using the R package “EnhancedVolcano” (version 1.18.0) and pheatmap (version 1.0.12).

### Lipid extraction from bacteria, chromatographic separation and identification of lipids

Lyophilized *Bifidobacterium animalis* subsp. *lactis* 420 (B420^TM^) was provided by Danisco Deutschland GmbH (Niebull, Germany). The other bacterial strains were grown overnight in 30 mL Luria-Bertani (LB) medium, except for *L. rhamnosus*, which was grown in De Man, Rogosa and Sharpe (MRS) medium and incubated for 24 h at 37 °C and 180 rpm. Bacteria were then pelleted for 5 min at 3000 rpm and the pellet was washed twice with PBS before proceeding to lipid extraction.

For lipid extractions, 10–100 g of fresh or lyophilized bacteria were rehydrated with 3 volumes of H_2_O. After adding 3.75 volumes of chloroform/methanol (1:2) the mixture was stirred and incubated overnight. First 3 volumes of chloroform and then 3 volumes of 40 mM acetic acid were added, each step followed by 1 min vigorous shaking. The solution was filtered and transferred to a separatory funnel to allow separation of the organic and inorganic phases. The organic phase was collected and evaporated and lipid extracts were stored in glass flasks at −20 °C.

### Phospholipid determination

Phospholipid (PL) estimation was performed with a colorimetric method based on formation of complexes between PLs and ammonium ferrothiocyanate (Charles et al, 1980). Bacterial extracts and PL standards were dissolved in 2 mL chloroform; 1 mL ammonium ferrothiocyanate (prepared by dissolving 27 g of ferric chloride (FeCl_3_ · 6 H_2_O) and 30 g of ammonium thiocyanate (NH_4_SCN) in 1000 mL H_2_O) was added and the mixture vortexed for 1 min. To separate the two phases, samples were centrifuged at 700×*g* for 10 min at RT. The lower phase was transferred to a quartz cuvette and absorbance at 488 nm was measured.

### Lipid extraction from bacteria, chromatographic separation and identification of lipids

Assay-guided chromatographic separation of crude lipid extracts from *B. lactis* B420^TM^ was achieved in four steps. First, the crude extract was purified by silica flash column chromatography (Teledyne Rf combiflash, 40 g silica column) in chloroform by using a 0– 100% gradient of chloroform/methanol (7:3) in 50 min and a flow rate of 40 mL/min. Individual vials of 20 mL were collected and screened by thin layer chromatography (TLC) using phosphomolybdic acid stain for detection. Vials with similar content (determined by Rf) were pooled together, evaporated and the residue was solubilized in ethanol and tested for LRH-1 activity by luciferase-based reporter assays. Pool 1 was further fractionated by silica flash column chromatography (Teledyne Rf combiflash, 12 g silica column) using a 0– 100% hexane and ethyl acetate gradient in 50 min and a flow rate of 30 mL/min. Individual vials of 20 mL were collected and analyzed by TLC. Vials with similar content were pooled, evaporated, and tested again for LRH-1 activity. Pool 1.5, was then further purified by HPLC (Shimadzu LC20-AT) with a preparative Reprosil 100 silica column (5 μM, 250 × 10 mm) using 0–100% ethyl acetate in hexane over 90 min and a flow rate of 4 mL/min. Individual vials were collected and analyzed by TLC. Again, vials with similar content (determined by Rf) were combined, evaporated and tested for LRH-1 activity. Pool 1.5.3 was further purified by HPLC (Shimadzu LC20-AT) with a preparative Reprosil 100 silica column (5 μM, 250 × 10 mm) and dichloromethane (A)/methanol (B) as mobile phase (0–2.5% B in 45 min) in a flow rate of 4 mL/min. Fraction 1.5.3.47, eluting at 1% B after 32.1 min, was analyzed by 1D NMR (1H, 13 C), 2D NMR (COSY, HMBC, HSQC) and GC-MS (Appendix Fig S1–S7).

To detect DLPC in bacteria extracts, a MicrOTOF II ESI mass spectrometer was used with an electrospray ionization (ESI) source operating in positive ion mode. Parameters were set as follows: capillary voltage: 4500 V; nebulizer pressure: 12 bar; drying temperature: 180 °C; drying gas flow: 10 L/min; end plate offset: 80 V.

### Lipid analysis by GC-MS/MS

Gas chromatography-mass spectrometry (GC-MS) was performed to assess the lipid profile of *B. lactis* B420. For this aim, lipids were extracted according to a modified Bligh and Dyer procedure (Jensen, 2008). Six hundred mg of lyophilized or fresh bacteria were dissolved in 1 mL 1 M HCl and heated to 80 °C for 1 h. Then, 1.5 mL methanol and 1 mL chloroform were added and the mixture was vortexed for 1 min. Thereafter, 1 mL water and 2 mL chloroform were added, and the mixture was vortexed again for 1 min. The mixture was centrifuged for 10 min at 4500 rpm and the chloroform phase was transferred into a glass vial and evaporated. For fatty acid methylation, 1 mg of extract was dissolved in 50 μL of hexane and 25 μL of trimethylsulfonium hydroxide (TMSH) solution (0.25 M in methanol) were added, and the solution was heated to 100 °C for 10 min. The resulting residue was dissolved in 100 μL hexane and subjected to GC-MS/MS measurement. Gas chromatography was performed on a Trace GC ultra (Thermo Fisher Scientific, Waltham, MA) using an OPTIMA 5 MS, 0.25 μm, 30 m, 25 mm ID column (Macherey-Nagel). Nitrogen was used as carrier gas. Throughout the measurements a constant gas linear velocity of 20 mL/min was maintained. The injection volume was 0.1 μL with a split ratio of 12:1. The initial oven temperature was set to 50 °C, immediately heated up to 80 °C at 20 °C/min, then ramped up to 280 °C with 6 °C/min and held for 2 min. MS/MS analysis was performed with a ISQ Single Quadruple GC-MS System (Thermo Fisher Scientific) mass spectrometer. The emission current was set to 50 μA and an ionization energy of 70 eV was applied. The ion source temperature was set to 200 °C. For ion fragmentation, methane was used as the collision-induced (CI) gas. Data was acquired in a mass range of *m/z* 41–600, and MS/MS acquisition of all compounds was performed in positive mode, fixed collision energy of 30 eV and dwell time of 0.2 s. Qualitative analysis was performed using Thermo Xcalibur 3.1.66.10 software and the NIST Database.

### Protein purification

The ligand binding domain (LBD, residues 299-541) of human LRH-1 (Uniprot O00482) was cloned into the IPTG-inducible pETM-11 vector (EMBL collection) that incorporates a His_6_-ta*g* followed by a tobacco etch virus (TEV) protease cleavage site N-terminally to the inserted gene. This expression plasmid was transformed into *E. coli* Rosetta^TM^ 2 (DE3) (Novagen) and bacterial cultures were grown at 37 °C in LB medium to an OD_600_ of 0.6. Protein expression was induced using 0.5 mM isopropyl β-D-1-thiogalactopyranoside (IPTG) and cultures were grown further at 18 °C for 16 h. Cells were harvested by centrifugation at 5000×*g* at 4 °C for 20 min and the pellets were resuspended in 30 mL of lysis buffer (300 mM NaCl, 20 mM Tris-HCl pH 7.4, 1 mM CHAPS, 10% Glycerol, 5 mM DTT) freshly supplemented with EDTA-free complete protease inhibitor (Roche). Bacteria were lysed via sonication on ice. Cell lysates were centrifuged (40,000×*g* for 90 min at 4 °C) and the supernatant was applied to a HisTrap HP column (Cytiva) pre-equilibrated in buffer (20 mM Tris-HCl pH 7.4, 200 mM NaCl, 1 mM CHAPS, 10% Glycerol, 5 mM DTT). The protein was eluted with an imidazole gradient.

Fractions containing LRH-1 LBD were pooled, treated with TEV-protease to cleave off the His_6_-tag and dialyzed overnight at 4 °C against 500 mL of lysis buffer. The sample was then subjected to subtractive HisTrap chromatography. Fractions containing LRH-1 LBD were concentrated to 1.5 mL by centrifugal ultrafiltration using a 10 kDA Amicon Ultra tube (Millipore). The protein was further purified using a HiLoad 16/60 Superdex^®^ 200 PG column (GE Healthcare) pre-equilibrated in 20 mM Tris-HCl pH 7.4, 150 mM NaCl, 1 mM CHAPS, 10% Glycerol, 10 mM DTT. The protein was flash frozen and stored at −80 °C until use.

### Microscale thermophoresis

To determine binding affinities, microscale thermophoresis (MST) was performed using the Monolith NT.115 instrument (NanoTemper Technologies). The purified LRH-1 LBD was labeled using the Monolith NT Protein Labeling Kit GREEN-NHS following the manufacturer´s procedure. Before labeling, protein buffer was exchanged to 20 mM HEPES, pH 7.4 by gel filtration chromatography with a PD SpinTrap G-25 column (Cytiva). Unbound dye was removed by overnight dialysis with a 3.5 KDa Slide-A-Lyzer MINI Dialysis Device (ThermoFisher Scientific). The labeled LRH-1 LBD concentration was adjusted to 60 nM in assay buffer (150 mM NaCl, 10 mM Tris-HCl pH 7.4, 5% glycerol, 0.1% N-octyl β-D-glucopyranoside, 0.05% Tween-20). Lipid stocks in ethanol were dissolved in assay buffer, vortexed and sonicated. A series of sixteen 1:1 dilutions of 20 μL was prepared for each lipid ligand. Each dilution was mixed with 20 μL of labeled LRH-1 LBD to a final concentration of protein of 30 nM and final lipid concentration ranging from 1 mM to 30.5 nM. Samples were incubated for 30 min at RT in the dark and loaded into Monolith NT.115 capillaries (NanoTemper Technologies). MST was measured using a Monolith NT.115 instrument at 22 °C. Instrument parameters were set to 60% LED power and 40% MST power. MST traces were analyzed with the MO. Affinity Analysis software version 2.2.4 (NanoTemper Technologies) selecting the K_d_ binding model. The cold region was defined as the 1 s region before the T-Jump while the hot region was defined as 0.5–1.5 s after the T-Jump to minimize possible temperature-dependent artifacts (López-Méndez et al, 2021).

### Western blotting

For Western Blot analysis, cells seeded in 6-well plates were lysed in 100 μL RIPA Buffer (50 mM Tris pH 7.4, 150 mM NaCl, 0.1% SDS, 1% NP-40, 0.5% sodium deoxycholate, freshly added Complete™ Protease Inhibitor Cocktail (Roche)) on ice for 15 min. Lysates were centrifuged for 20 min at 14,000×*g* and 4 °C, and total protein concentration of supernatants was determined with the Pierce™ BCA Assay Kit (Thermo Fisher). Samples were boiled in Laemmli buffer for 5 min at 95 °C and equal protein amounts (30–50 µg/sample) were separated on a 12% SDS-polyacrylamide gel. Then, proteins were blotted onto a PVDF membrane (Roche) in Blotting Buffer (25 mM Tris, 192 mM glycine, 0.1% SDS, 21.5% methanol). Non-specific binding was blocked in 5% non-fat dry milk/TBS-T (137 mM NaCl, 2.7 mM KCl, 15 mM Tris, 0.1% Tween-20) for 1 h and afterwards primary antibodies diluted in 5% BSA/TBS-T were incubated overnight at 4 °C. After washing three times for 5 min with TBS-T, incubation with peroxidase-conjugated secondary antibodies in 5% BSA/TBS-T was performed for 2 h at RT followed by three additional washing steps. An ECL solution (250 mM Luminol, 90 mM p-coumaric acid, 10 mM Tris) with 1% hydrogen peroxide was applied on the membranes and proteins were detected with an Image Quant LAS4000 (GE Healthcare).

### Cell culture and treatments

The human cell lines Caco-2 (ATCC HTB-37) and LS174T (ATCC CL-188) were obtained from American Type Culture Collection (ATCC) and cultured in Iscove’s Modified Dulbecco’s Medium supplemented with 10% FCS, 4 mM L-glutamine and 10 μg/mL gentamicin (all from Sigma-Aldrich, Steinheim, Germany). HEK293T were cultured in Dulbecco’s Modified Eagle’s Medium (DMEM) supplemented with 5% FBS, 4 mM L-glutamine and 10 μg/mL gentamicin. Cells were grown at 37 °C and 5% CO_2_ in a humidified atmosphere and passaged at 70-80% confluency every 2–3 days. Cell lines were regularly tested for mycoplasma contamination using the Venor^®^GeM Classic kit according to the manufacturer’s protocol (Minerva Biolabs, Berlin, Germany).

The LRH-1 inhibitor 3d2 (ID: 16728690) was synthesized according to the initial publication from Benod et al (Benod et al, 2013) by ChemBridge Corp (San Diego, USA). SR1848 (SML 1513) was purchased from Sigma-Aldrich. Stock solutions of 3d2 and SR1848 were kept as 20 mM stocks in DMSO and stored at −20 °C or −80 °C. Lipids and bacteria extracts were dissolved in ethanol and stored at −20 °C.

### Luciferase reporter assays

For luciferase-based reporter assays, cells were transiently transfected with expression and luciferase reporter plasmids using JetPei^TM^ (Polyplus Transfection). Plasmids used in this study are listed in Table 2. The LRH-1 reporter containing five copies of the LRH-1 response element in the pGL3 plasmid (LRH-1 5xRE) has been described previously (Schoonjans et al, 2002). The empty vector pGL3 basic was purchased from Promega. Co-transfection of the β-galactosidase expression plasmid (Invitrogen) served for normalization for transfection efficiency. Expression plasmids for nuclear receptors and their cognate luciferase reporters were: mouse PPARα, human FXRα, TK-PPREx3-Luc (kindly provided by David Moore), human PPARγ1, human RXR and human PPARγ1 luciferase reporter (kindly provided by Kristina Schoonjans). One day after transfection, cells were reseeded into 96-well plates, stimulated with medium control or 200 nM 4HT for 4–6 h to induce LRH-1 expression, and then treated for 16–18 h with the indicated substances in cell culture medium containing 10% charcoal-treated FCS, 4 mM L-glutamine and 10 μg/mL gentamicin. Working solutions of bacteria lipid extracts and lipids were prepared by diluting stock solutions with medium containing 10% charcoal-treated FCS, 4 mM L-glutamine and 10 μg/mL gentamicin, vortexed for 1 min and incubated in a bath sonicator for 10 min at 37 °C. For pharmacological inhibition of LRH-1, cells were preincubated with 3d2 (10 μM) for 24 h and the inhibitor was maintained in the medium during treatment with the tested substances. SR1848 (1 μM) was added to cells together with the tested substances 16–18 h before the assay. To assess luciferase and β-galactosidase activity, cells were lysed in lysis buffer (100 mM K_2_HPO_4_, 0.2% Triton X-100, 1 µM DTT, pH 7.8) for 15 min on ice, collected and centrifuged at 500×*g* for 10 min. Supernatants were transferred into white 96-well plates and luminescence was measured with an Infinite® 200 PRO plate reader (TECAN) by automatically injecting an ATP solution (10 mM ATP, 20 mM MgCl_2_, 35 mM glycyl-glycine) and the luciferin substrate (470 µM firefly luciferin, 270 µM coenzyme-A, 20 mM MgCl_2_, 35 mM glycyl-glycine). β-Galactosidase activity was assessed as described previously (Michalek et al, 2022). Luminescence was calculated as relative light units (RLU) by normalizing light units assessed with the luciferase assays to the values obtained with the β-galactosidase assay. Unless otherwise indicated, graphs show luciferase activity normalized to the vehicle-treated condition without LRH-1 induction.

**Table 2:** Source or reference of plasmids used in the study.

Recombinant DNA	Reference or source	Identifier or catalogue number
IPTG-inducible pETM-11 vector	EMBL collection	N/A
pGL3 promoter vector	Gift from Kristina Schoonjans	Promega #E1761
pGL3 hPPARγ1 p3000	Gift from Kristina Schoonjans	N/A
pSG5	Agilent Technologies	#216201
pSG5 hPPARγ1	Gift from Kristina Schoonjans	N/A
pSG5 hRXRα	Gift from Kristina Schoonjans	N/A
pCMX mPPARα	Gift from David Moore	N/A
pTK-luciferase3x(PPRE)	Gift from David Moore	N/A
pGL3 basic (pGL3)	Promega	#E1751
pcDNA3.1 hGR (GR)	This laboratory	N/A
GRE2-tk-luc (GRE)	Liden et al, 1997	N/A
TKpGL3 LRH-1-RE 5x (5xRE)	Schoonjans et al, 2002	N/A

### Site-directed mutagenesis

The LRH-1 double mutant F342W/I416W, which displays reduced ligand binding activity, was generated with the QuikChange II XL Site-Directed Mutagenesis Kit (Agilent Technologies) in the pF-5xUAS-MCS-SV40 expression plasmid containing the human Flag-tagged LRH-1 in two steps, following the manufacturer´s instructions. Primers used are indicated in Table 1. Introduced mutations were confirmed by sequencing.

### Generation of 4-hydroxytamoxifen-inducible cell lines

GEV16-mediated inducible overexpression of LRH-1 was achieved as described previously (Vince et al, 2007; Lambrecht et al, 2023). LS174T cells were transduced with HEK293T-derived lentiviruses containing the GEV16 plasmid (pHCA/GAL4(1-93).ERα.VP16) and a stable cell line was generated by selection with 1 mg/mL hygromycin B. The second step included transduction of GEV16-expressing LS174T cells with lentiviruses containing the pF-5xUAS-MCS-SV40-Puro-based expression plasmid with either human Flag-tagged LRH-1, the ligand binding mutant human Flag-tagged LRH-1 F342W/I416W or murine LRH-1. GEV16-LS174T cells transduced with EGFP-expressing lentiviruses were used as control. Stable cell lines were generated by selection with 8 µg/mL puromycin. Overexpression of LRH-1 or EGFP was achieved by treatment of cells with 200 nM 4-hydroxytamoxifen (4-HT) for 4 h, unless stated differently.

### MTT cell viability assay

Cell viability after indicated treatments was estimated with the 3-(4,5-dimethylthiazol-2-yl)-2,5-diphenyl-tetrazolium bromide (MTT, Sigma) viability assay. After removing the cell culture medium, cells in 96-well plates were incubated in 10% MTT/medium solution for 1 h. Generated violet formazan crystals in living cells were dissolved in DMSO and absorbance was measured at 562 nm with the Infinite® 200 PRO plate reader (TECAN). Values were normalized to those of vehicle-treated cells.

### Statistical analysis

Statistical analysis was performed using GraphPad Prism 7.0. If not stated differently, data are represented as mean ± standard deviation (SD), with the indicated number of independent experiments with 3 or 4 technical replicates. Statistical significance was calculated by unpaired two-tailed Student’s *t* test when comparing two groups, one-way or two-way ANOVA when comparing three or more dependent groups, respectively. Suitable post-hoc tests (Tukey’s or Sidak’s) were applied and are stated in respective figure legends. *P* values are indicated in the figures or figure legends.

### Graphics

The visual abstract was created with BioRender.com.

## Supplementary information

Table EV1

Appendix

Source data Fig. 1

Source data Fig. 2

Source data Fig. 3

Source data Fig. 4

Source data Fig. 5

Source data Fig. 6

Peer Review File

Expanded View Figures

## Data Availability

RNA-seq files generated in this study are openly available in GEO at https://www.ncbi.nlm.nih.gov/sra/PRJNA1462643, reference number PRJNA1462643. The source data of this paper are collected in the following database record: biostudies:S-SCDT-10_1038-S44321-026-00516-3.
