# Supplementary material for: Microbiome-host communication via vaccenic acid modulates LRH-1/NR5A2 activity and ameliorates metabolic liver disease
Source: EMBO Mol Med. 2026 Sep 17;18(10):4059–89. doi: 10.1038/s44321-026-00516-3 (PMC13658251; doi:10.1038/s44321-026-00516-3)
Supplement: Supplementary file 1 [file 44321_2026_516_MOESM10_ESM.pdf]

Expanded View Figures

Figure EV1. (related to Fig. 1): Characterization of the 4-HT-inducible expression system and control experiments related to LRH-1 transactivation.

All experiments were performed in LS174T cells, except those shown in panel H. LRH-1 and EGFP cells refer to cells in which human Flagged-tag LRH-1 or EGFP expression was induced by 4-HT treatment (4 h, unless differently indicated). *P* values are indicated in the figures. (**A**) Quantification of human *LRH-1* mRNA in LRH-1 or EGFP cells. Cells were treated with 4-HT for 6 h and *LRH-1* mRNA was measured by RT-qPCR. A representative result showing mean values ± SD of three replicates of two independent experiments is shown. *P* values were calculated with a two-tailed *t* test, ns: not significant ($P = 0.9514$). (**B**) Immunoblots of human LRH-1 in cell lysates after expression of LRH-1 or EGFP with 4-HT. Cells were treated for 6 h with 4-HT or with its vehicle. LRH-1 expression was detected with anti-LRH-1 or anti-Flag antibodies. Tubulin was used as loading control. (**C**) LRH-1 transcriptional activity in LRH-1 or EGFP cells after o.n. induction with the indicated concentrations of 4-HT. Mean values ± SD of technical triplicates are shown. Two-way ANOVA, *P* values compared to vehicle-treated sample are indicated. RLU relative luciferase units. (**D**) Luciferase activity in LRH-1 or EGFP cells transfected with the pGL3 control vector or LRH-1 reporter. Mean values ± SD. Two-way ANOVA. (**E**) LRH-1 transcriptional activities in cells with 4-HT-inducible LRH-1 or EGFP expression, stimulated o.n. with B420 extract. Mean values of technical triplicates ± SD of a typical experiment ($n = 2$) are shown. Two-way ANOVA. (**F**) Cell viability of LRH-1 and EGFP cells treated with increasing concentrations of B420 extracts. Respiratory activity as measured by MTT is shown (mean ± SD of a representative experiment, $n = 3$). Comparison of all means by two-way ANOVA revealed no significant differences among any of the groups (all $P > 0.1609$). (**G**) Cell viability (MTT assay) of LRH-1 cells treated with the LRH-1 inhibitors 3d2 or SR1848. Mean values ± SD of a representative experiment ($n = 2$) are shown. Paired two-tailed *t* tests were performed to compare each treatment with the control (−). (**H**) Transcriptional activities of endogenous LRH-1 and cell viability (respiratory activity, MTT assay) in Caco-2 cells stimulated with B420 extracts in the absence or presence of the LRH-1 inhibitor 3d2. Mean ± SD ($n = 2$). Two-way ANOVA analysis. Comparison of all cell viability means revealed no differences among any of the groups (all $P > 0.5292$). (**I**) LRH-1 transcriptional activities in LS174T with 4-HT-inducible expression of wild-type LRH-1 or LRH-1 F342W/I416W stimulated with increasing doses of B420 extract. Mean ± SD of one experiment performed in triplicates. Two-way ANOVA (comparison of mean values of B420 treatments to vehicle-treated control, +4-HT group). (**J**) Effect of lipid extracts (50 µg/mL) from different *E. coli* strains on LRH-1 transcriptional activity in LRH-1-expressing LS174T cells. Mean ± SD of at least three independent experiments performed in technical triplicates. Unpaired two-tailed *t* tests were performed to compare each treatment with the control. (**K**) Cell viability (MTT assay) in LRH-1-expressing LS174T cells treated with lipid extracts from bacterial strains indicated (200 µg/mL).

© The Author(s)

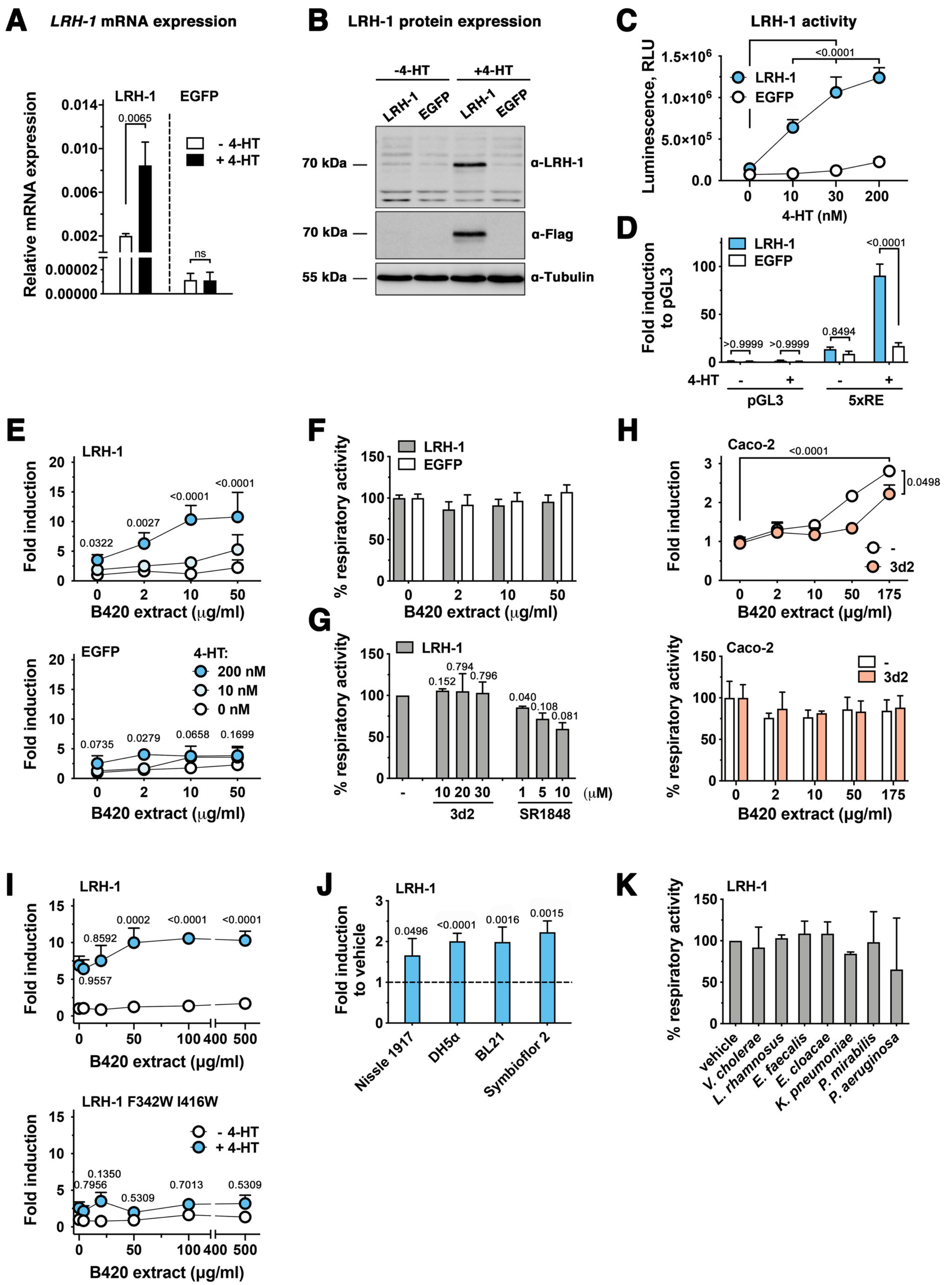
A
LRH-1 mRNA expression
Relative mRNA expression
LRH-1
EGFP
0.0065
ns
- 4-HT
+ 4-HT
B
LRH-1 protein expression
-4-HT
+4-HT
LRH-1
EGFP
70 kDa
α-LRH-1
α-Flag
55 kDa
α-Tubulin
C
LRH-1 activity
Luminescence, RLU
<0.0001
4-HT (nM)
D
Fold induction to pGL3
>0.9999
0.8494
<0.0001
4-HT
pGL3
5xRE
E
Fold induction
B420 extract (μg/ml)
4-HT:
200 nM
10 nM
0 nM
F
% respiratory activity
G
3d2
SR1848
(μM)
H
Caco-2
<0.0001
0.0498
I
LRH-1 F342W I416W
J
Fold induction to vehicle
Nissle 1917
DH5α
BL21
Symbioflor 2
K
vehicle
V. cholerae
L. rhamnosus
E. faecalis
E. cloacae
K. pneumoniae
P. mirabilis
P. aeruginosa

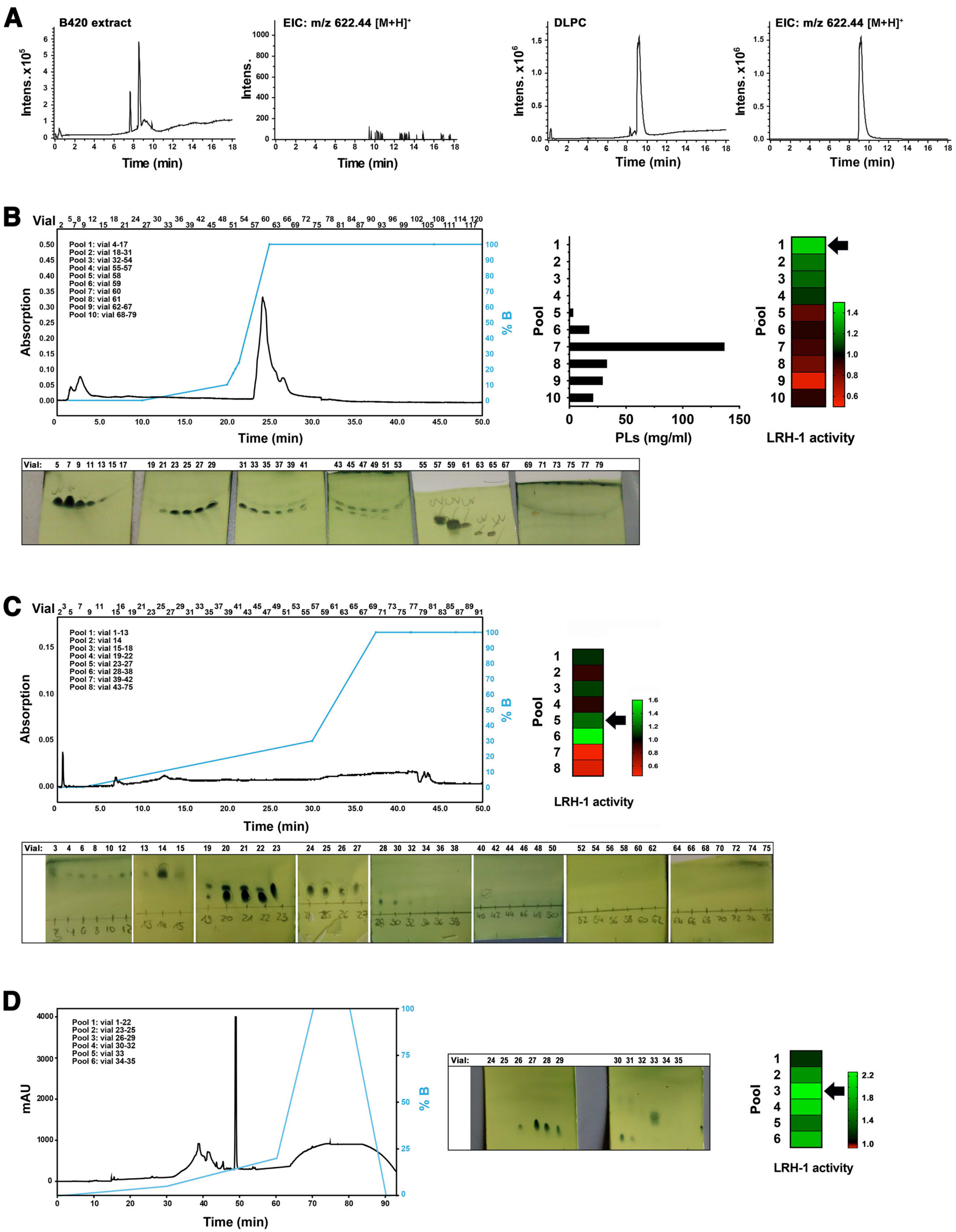
A
B420 extract
EIC: m/z 622.44 [M+H]+
DLPC
EIC: m/z 622.44 [M+H]+
Intens.x10^5
Intens.
Intens. x10^6
Time (min)
B
Vial
Pool 1: vial 4-17
Pool 2: vial 18-31
Pool 3: vial 32-54
Pool 4: vial 55-57
Pool 5: vial 58
Pool 6: vial 59
Pool 7: vial 60
Pool 8: vial 61
Pool 9: vial 62-67
Pool 10: vial 68-79
Absorption
% B
Time (min)
Pool
PLs (mg/ml)
LRH-1 activity
C
Vial
Pool 1: vial 1-13
Pool 2: vial 14
Pool 3: vial 15-18
Pool 4: vial 19-22
Pool 5: vial 23-27
Pool 6: vial 28-38
Pool 7: vial 39-42
Pool 8: vial 43-75
Absorption
% B
Time (min)
Pool
LRH-1 activity
D
Pool 1: vial 1-22
Pool 2: vial 23-25
Pool 3: vial 26-29
Pool 4: vial 30-32
Pool 5: vial 33
Pool 6: vial 34-35
mAU
% B
Time (min)
Vial:
Pool
LRH-1 activity

◀ **Figure EV2. (related to Fig. 2): Chromatographic analysis of B420 extracts and identification of LRH-1-stimulating compounds.**

(**A**) Chromatograms and extracted ion chromatograms (EIC) for *m/z* 622.44 (right panel) of the complete B420 extract (left) or of DLPC (right). A MicrOTOF II ESI mass spectrometer was used with an electrospray ionization (ESI) source operating in positive ion mode. Parameters were set as follows: capillary voltage: 4500 V; nebulizer pressure: 12 bar; drying temperature: 180 °C; drying gas flow: 10 l/min; End plate offset: 80 V. (**B**) Silica flash column chromatography of crude B420 extract using a 0–100% gradient of chloroform (**A**) and chloroform/methanol 7:3 (**B**) in 50 min and a flow rate of 40 ml/min. Detection at 254 nm. An aliquot of every second vial was analyzed by thin layer chromatography (TLC) using phosphomolybdic acid stain. Vials with similar content were combined as indicated resulting in ten pools. Analysis of phospholipid (PL) content in these pools revealed that PL content peaked in pool 7, whereas pool 1 (arrow) promoted the strongest LRH-1 transactivation. (**C**) Silica flash column chromatography of pool 1 using a 0–100% hexane and ethyl acetate gradient (% B) in 50 min and a flow rate of 30 mL/min. Individual vials were collected and analyzed by TLC using phosphomolybdic acid stain. Vials with similar content were combined into 8 pools as indicated. Pools 5 and 6 showed strongest LRH-inducing activity. (**D**) Pool 5 was further purified by HPLC (Shimadzu LC20-AT) with a preparative Reprosil 100 silica column using hexane and ethyl acetate as mobile phase (0–100% in 90 min, % B) and a flow rate of 4 mL/min. Vials containing lipids were combined into six pools as indicated and tested for LRH-1 transactivation. Pool 3 (arrow) was further purified by a second HPLC (see text and Fig. 2A, B).

© The Author(s)

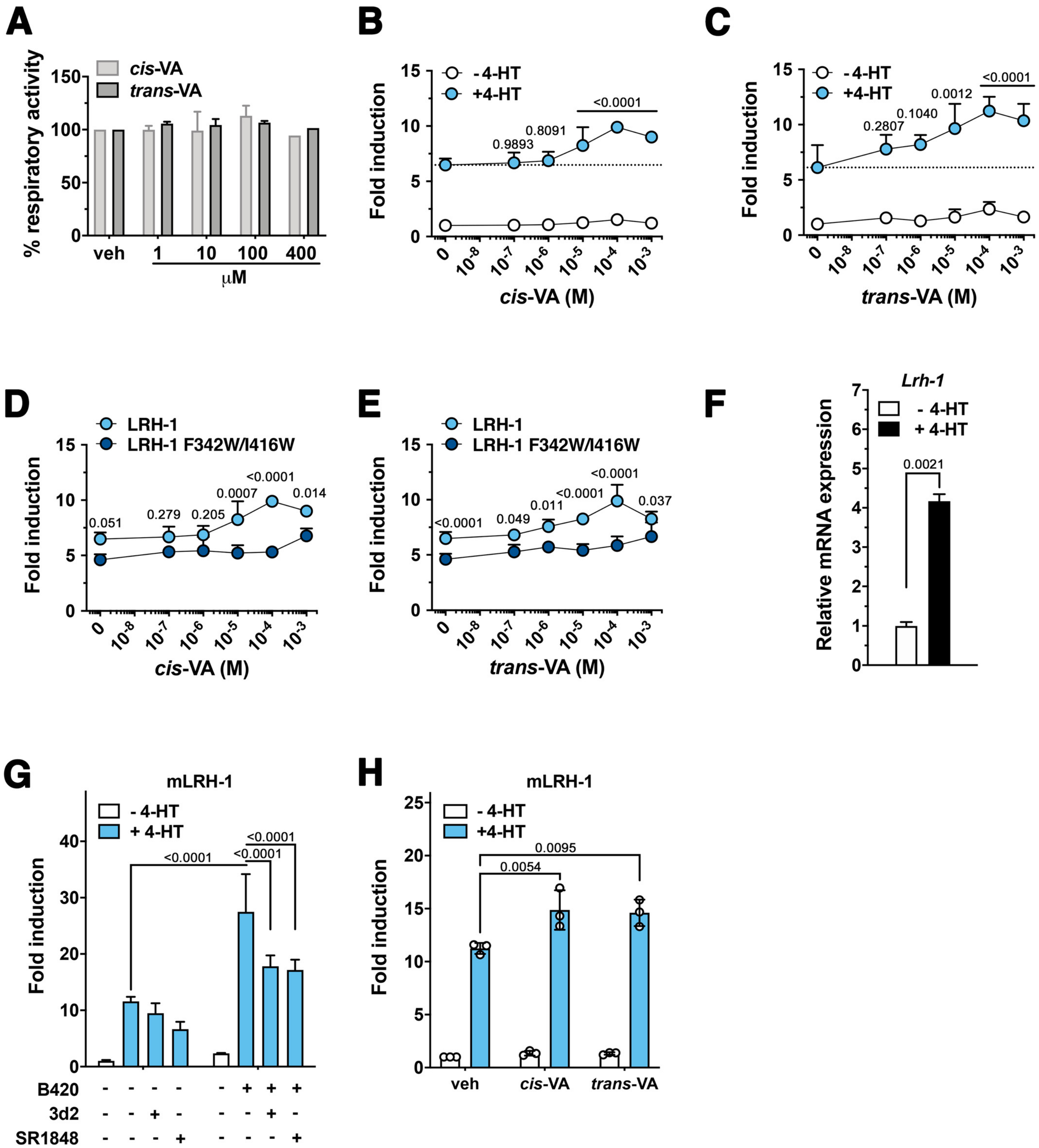
A
% respiratory activity
cis-VA
trans-VA
veh
1
10
100
400
μM
B
Fold induction
- 4-HT
+4-HT
0.9893
0.8091
<0.0001
cis-VA (M)
C
Fold induction
- 4-HT
+4-HT
0.2807
0.1040
0.0012
<0.0001
trans-VA (M)
D
Fold induction
LRH-1
LRH-1 F342W/I416W
0.051
0.279
0.205
0.0007
<0.0001
0.014
cis-VA (M)
E
Fold induction
LRH-1
LRH-1 F342W/I416W
<0.0001
0.049
0.011
<0.0001
<0.0001
0.037
trans-VA (M)
F
Lrh-1
Relative mRNA expression
- 4-HT
+ 4-HT
0.0021
G
mLRH-1
Fold induction
- 4-HT
+ 4-HT
<0.0001
<0.0001
<0.0001
B420
3d2
SR1848
H
mLRH-1
Fold induction
- 4-HT
+4-HT
0.0095
0.0054
veh
cis-VA
trans-VA

Figure EV3. (related to Fig. 2): Characterization of *cis*- and *trans*-VA-mediated LRH-1 transactivation; verification of murine LRH-1 transactivation by B420 and VA.

Experiments were performed in LS174T cells with 4-HT-inducible expression of wild-type or mutant human Flagged-tag LRH-1, or murine LRH-1 (4 h, unless differently indicated). (**A**) Cell viability (MTT assay) in LRH-1 cells treated with the indicated concentrations of *cis*- or *trans*-VA. Mean ± SD of two independent experiments performed in duplicates. (**B**, **C**) Effects of *cis*- (**B**) or *trans*-VA (**C**) on the transcriptional activity of LRH-1 in cells with uninduced (-4-HT) or induced expression of LRH-1 (+ 4-HT). Mean values of technical triplicates ± SD of one representative experiment ($n = 3$) are shown. Two-way ANOVA. (**D**, **E**) Effects of *cis*- (**D**) or *trans*-VA (**E**) on the transcriptional activity of wild-type or F342W/I416W mutant LRH-1. Mean values of technical triplicates ± SD of one experiment are shown. Two-way ANOVA comparing LRH-1 vs LRH-1 F342W/I416W for each concentration. (**F**) Quantification of murine *Lrh-1* mRNA by RT-qPCR in mLRH-1 with 200 nM 4-HT for 6 h. Mean ± SD of one experiment performed in duplicates. Unpaired two-tailed *t* test. (**G**, **H**) LRH-1 transcriptional activity in cells with 4-HT-inducible expression of mLRH-1. Cells were incubated with 200 µg/mL B420 extract (**G**), with 100 µM *cis*-VA or *trans*-VA (**H**) or the vehicle (ethanol, veh) for 16 h. For inhibition of LRH-1 activity, cells were treated with 3d2 or SR1848. Mean ± SD of one experiment performed in triplicates (**G**) or of three independent experiments in triplicates (**H**). Two-way ANOVA.

© The Author(s)

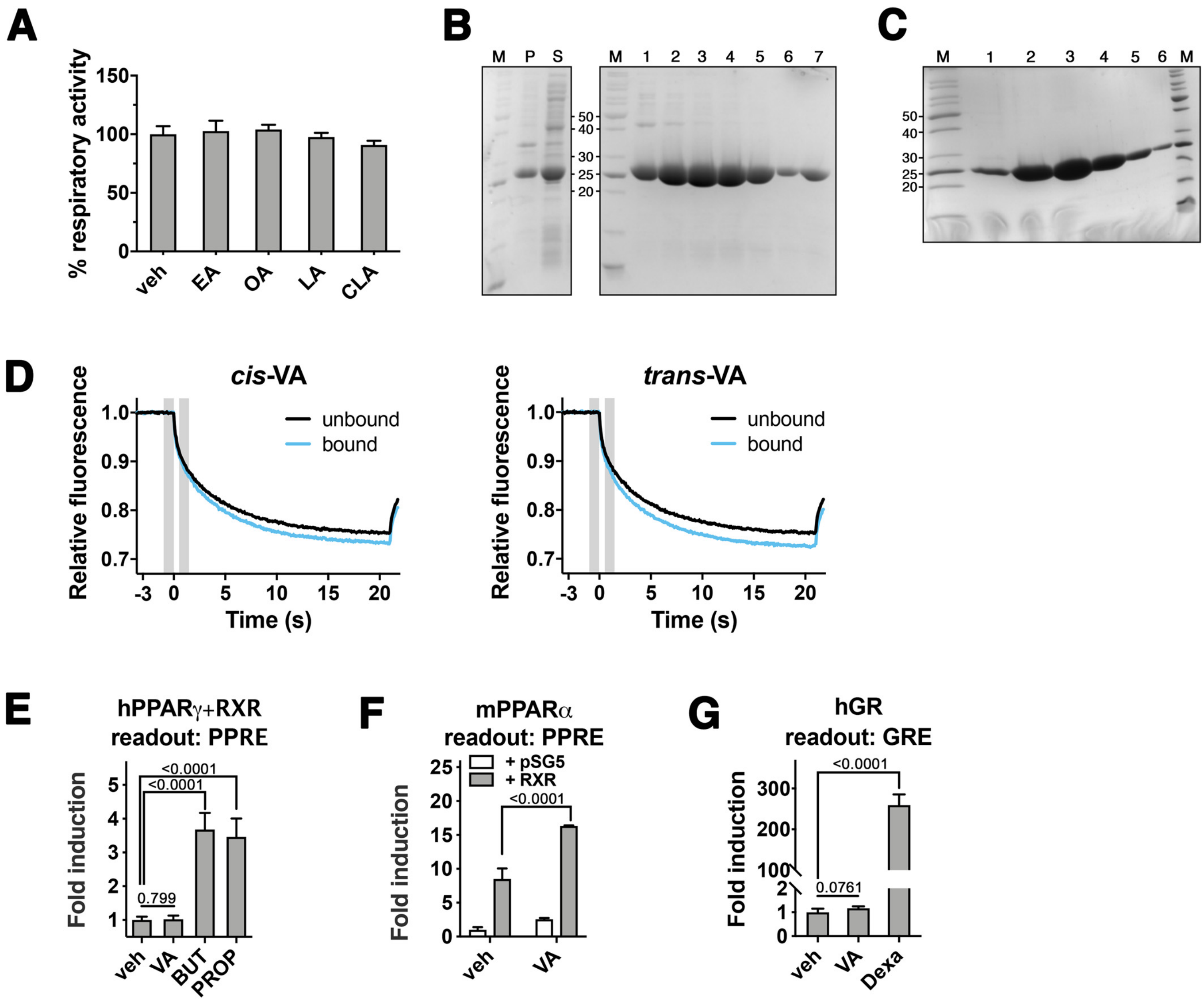

Figure EV4. **(related to Fig. 3): LRH-1 LBD purification and control experiments.**

(**A**) Cell viability (MTT assay) of LRH-1 cells treated with different fatty acids (100 µM) (elaidic acid, EA; oleic acid, OA; linoleic acid, LA; *cis*9, *trans*11-conjugated linoleic acid, CLA) or their vehicle (veh, ethanol). Mean values of triplicates ± SD of one experiment. (**B**) Coomassie-stained SDS-PAGE of different fractions obtained after Ni^{2+}-affinity chromatography to capture His-tagged LRH-1 LBD and elution with increasing concentrations of imidazole (see "Methods" section). M = Unstained Protein Standard, Broad Range (10–200 kDa, New England Biolabs); *P* = Bacterial pellet before purification; S = Supernatant before purification; 1–7 = LRH-1 LBD-containing fractions collected with an increasing gradient of imidazole. (**C**) Coomassie-stained SDS-PAGE of fractions of pure LRH-1 LBD after additional subtractive HisTrap chromatography and gel filtration. (**D**) Representative microscale thermophoresis (MST) traces for *cis*- and *trans*-VA. The "bound" curve represents the trace with 1 mM ligand and the "unbound" one the condition with the lowest ligand concentration (30.5 nM). The change in normalized fluorescence was derived by analyzing the 1 s region before the T-Jump and 0.5–1.5 s after the T-Jump in the MST traces (grey bars) and was plotted against the concentrations of ligand. (**E–G**) Transcriptional activities in LS174T cells after transfection of the indicated NRs and the cognate response element-containing luciferase reporter. *P* values after statistical analysis are indicated in the figures. (**E**) Human PPARγ/RXR activity after incubation with VA (30 µM, mixture of *cis* and *trans*). Butyrate (BUT) and propionate (PROP) (3 mM) were used as positive controls. PPRE PPARγ Response Element. Unpaired *t* test. (**F**) Murine PPARα activity after cotransfection with RXR or empty vector pSG5 control, and incubation with VA (30 µM, mixture of *cis* and *trans*). PPRE: PPAR Response Element. Two-way ANOVA. (**G**) Transcriptional activity of the glucocorticoid receptor (GR) after incubation with VA (150 µM, mixture of *cis* and *trans*) or 500 nM dexamethasone (Dexa) (GR agonist). Unpaired *t* test. GRE Glucocorticoid Response Element.

© The Author(s)

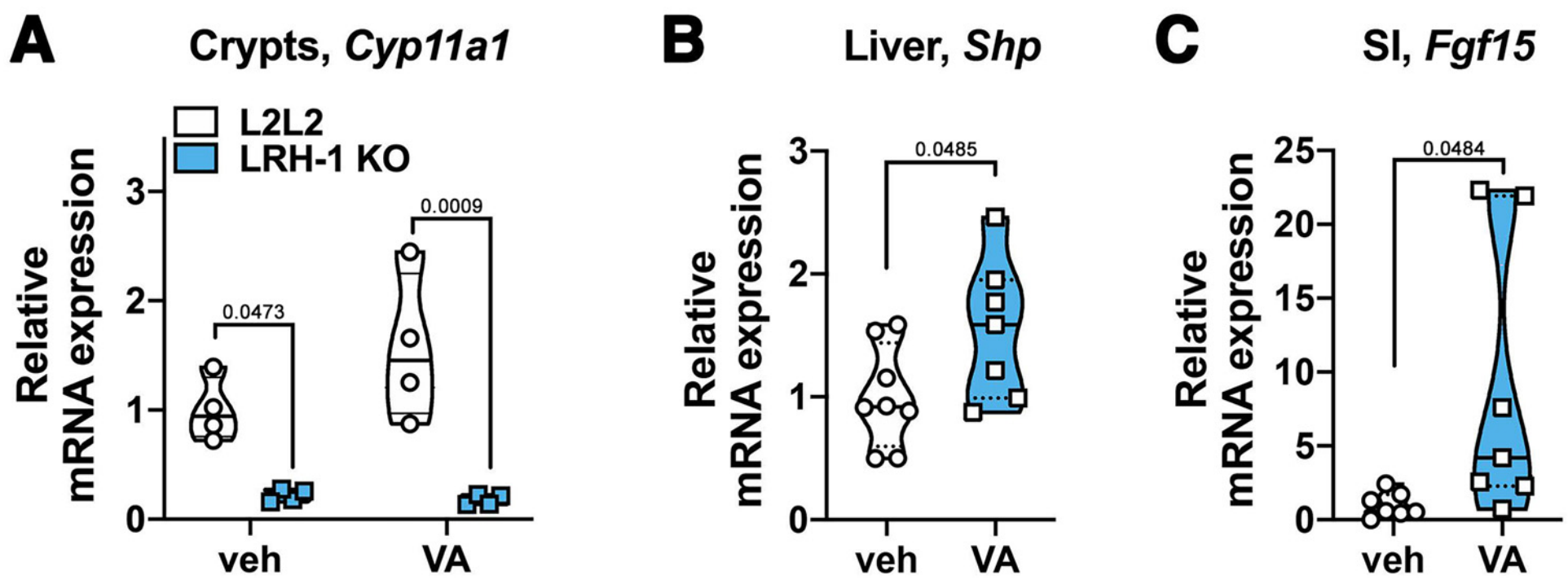

Figure EV5. (related to Fig. 4): VA-induced expression of steroidogenic enzymes in primary intestinal crypt cells requires the presence of LRH-1; investigation on FXR-SHP-FGF15 axis contribution to VA-induced effects.

(**A**) Primary crypt cells from small intestine (SI) of control LRH-1 L2L2 and LRH-1 VilCre mice with LRH-1 deletion in intestinal epithelial cells (LRH-1 KO) were isolated and cultured for 4 h ex vivo with 240 µM mixture of *cis*- and *trans*-VA or its vehicle control (veh). $N = 4$, two-way ANOVA with Tukey´s correction. Isolation and culture of intestinal crypts was done as described in Noti et al, 2010. (**B**, **C**) Increased expression of hepatic *Shp* (**B**) and intestinal *Fgf15* (**C**) in mice treated for 18 h with VA compared to its vehicle control, suggesting a complex crosstalk between VA-induced LRH-1 activation and the FXR-SHP-FGF15 axis. $n = 7$–8, each dot represents an individual mouse. Unpaired two-tailed t test.

 © The Author(s)

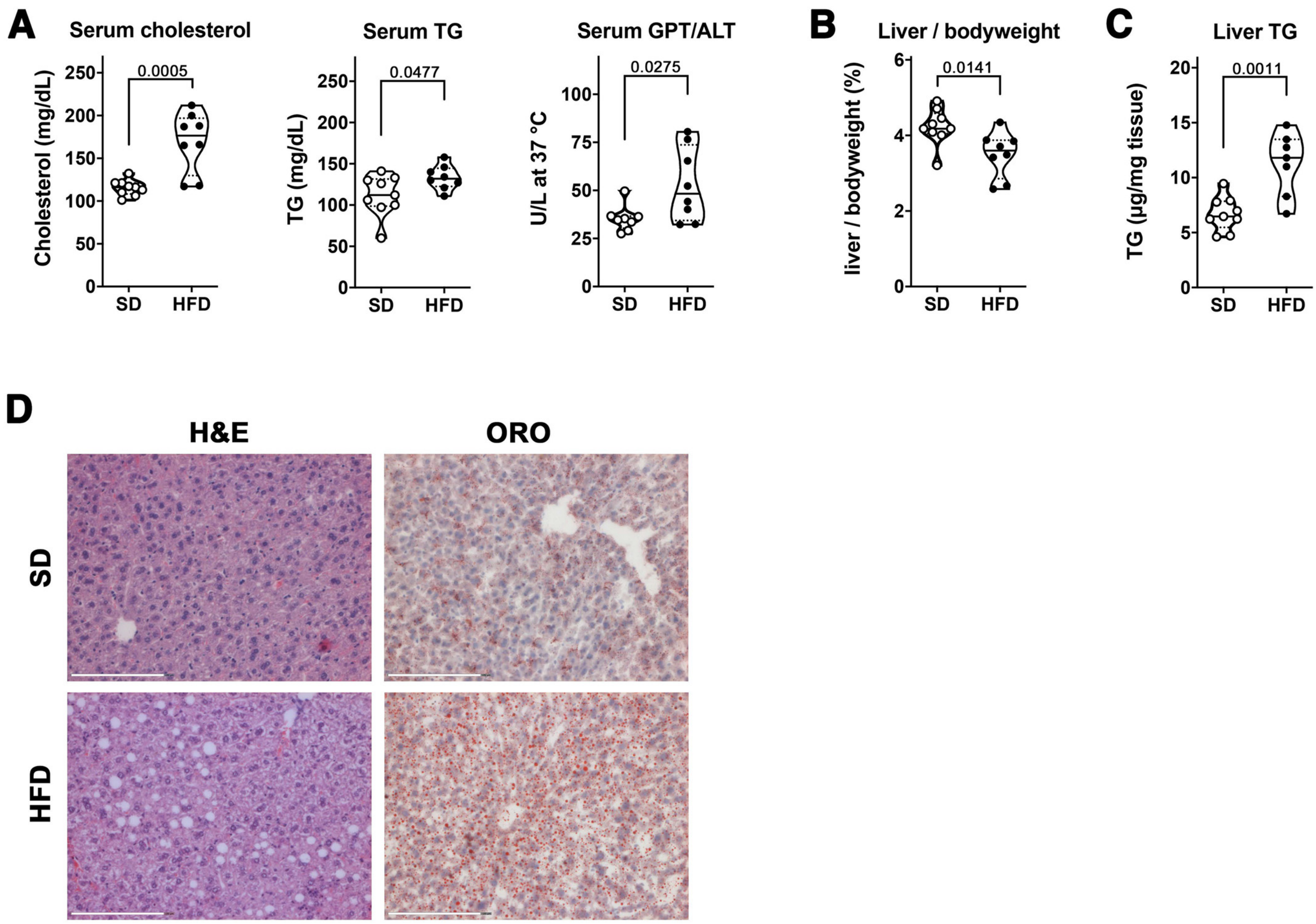

Figure EV6. (related to Fig. 5): Comparison of SD- and HFD-fed mice.

(**A**) Serum cholesterol, triglyceride (TG) and glutamic pyruvic transaminase/alanine transaminase (GPT/ALT) values in male C57BL/6 mice fed either with standard diet (SD) or a high-fat diet (HFD) for 15 weeks. Each dot represents an individual mouse. Unpaired two-tailed *t* test, SD: $n = 8$–9, HFD: $n = 8$. (**B**) Percentage of liver weight normalized by body weight. Unpaired two-tailed *t* test, SD: $n = 9$, HFD: $n = 8$. (**C**) Total liver TG. Unpaired two-tailed *t* test, SD: $n = 9$, HFD: $n = 7$. (**D**) Representative hematoxylin-eosin (H&E)- and Oil Red O (ORO)-stained liver sections from SD- or HFD-fed mice at the end of the 15-week diet. Scale bars represent 150 µm.

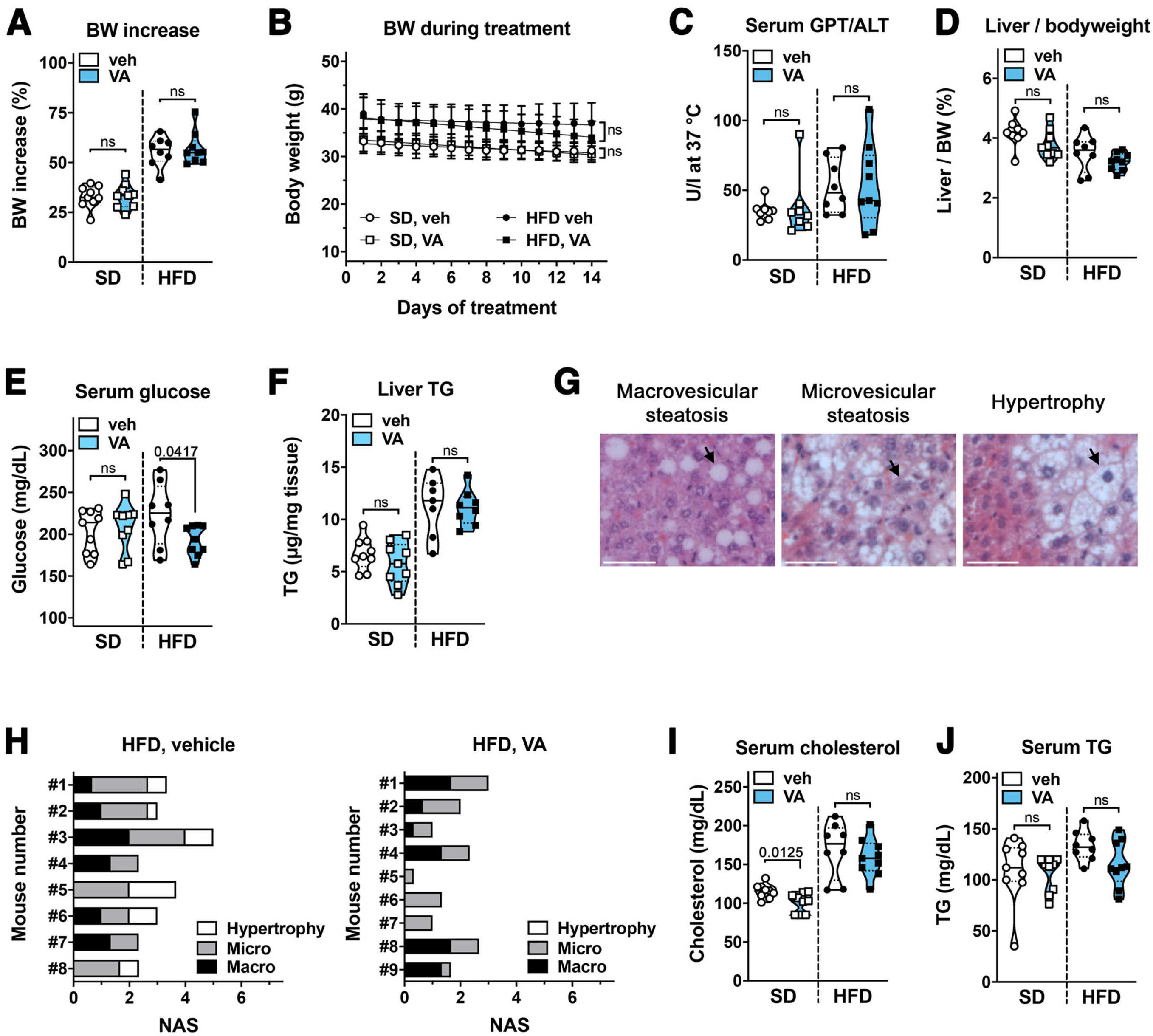

Figure EV7. (related to Fig. 5): Effects of VA administration in SD- or HFD-fed mice.

(**A**) Comparison of the bodyweight (BW) increase at the end of the SD or HFD between vehicle (veh)- and VA-treated mice. Unpaired two-tailed *t* tests, SD: veh $n = 9$, VA $n = 9$; HFD: veh $n = 8$, VA $n = 9$. (**B**) BW changes during the 2 weeks of treatment with vehicle control or VA. Values represent mean ± SD of $n = 18$ (SD) and $n = 17$ (HFD) mice. Mixed-effects analysis with Sidak's multiple comparisons test; ns: not significant. (**C–F**) Serum GPT/ALT levels (**C**), normalized liver weights (**D**), serum glucose (**E**) and liver triglycerides (TG) (**F**) after vehicle or VA treatment in SD- or HFD-fed mice. Unpaired two-tailed *t* tests, ns: not significant, SD: veh $n = 8$–9, VA $n = 9$; HFD: veh $n = 8$, VA $n = 9$. (**G**) Enlargements of H&E-stained liver sections to highlight different aspects of steatosis. Macrovesicular steatosis is characterized by hepatocytes containing a single large fat droplet or multiple smaller, well-defined droplets, which expand in the cell and displace the nucleus to the periphery. Microvesicular steatosis is defined by hepatocytes with distended, foamy cytoplasm and small lipid vesicles. Hypertrophy defines enlarged hepatocytes compared to neighboring normal hepatocytes, with an increase in their cytoplasmic volume. Scale bars represent 50 µm. Arrows indicate representative cells. (**H**) NAFLD activity score (NAS) in individual mice with relative contribution of macrovesicular steatosis, microvesicular steatosis and hepatocyte hypertrophy, as analyzed in H&E-stained liver sections. (**I, J**) Serum cholesterol (**I**) and TG (**J**) levels after vehicle or VA treatment in SD- or HFD-fed mice. Unpaired two-tailed *t* tests, ns: not significant, SD: veh $n = 9$, VA $n = 9$; HFD: veh $n = 8$, VA $n = 9$.

© The Author(s)

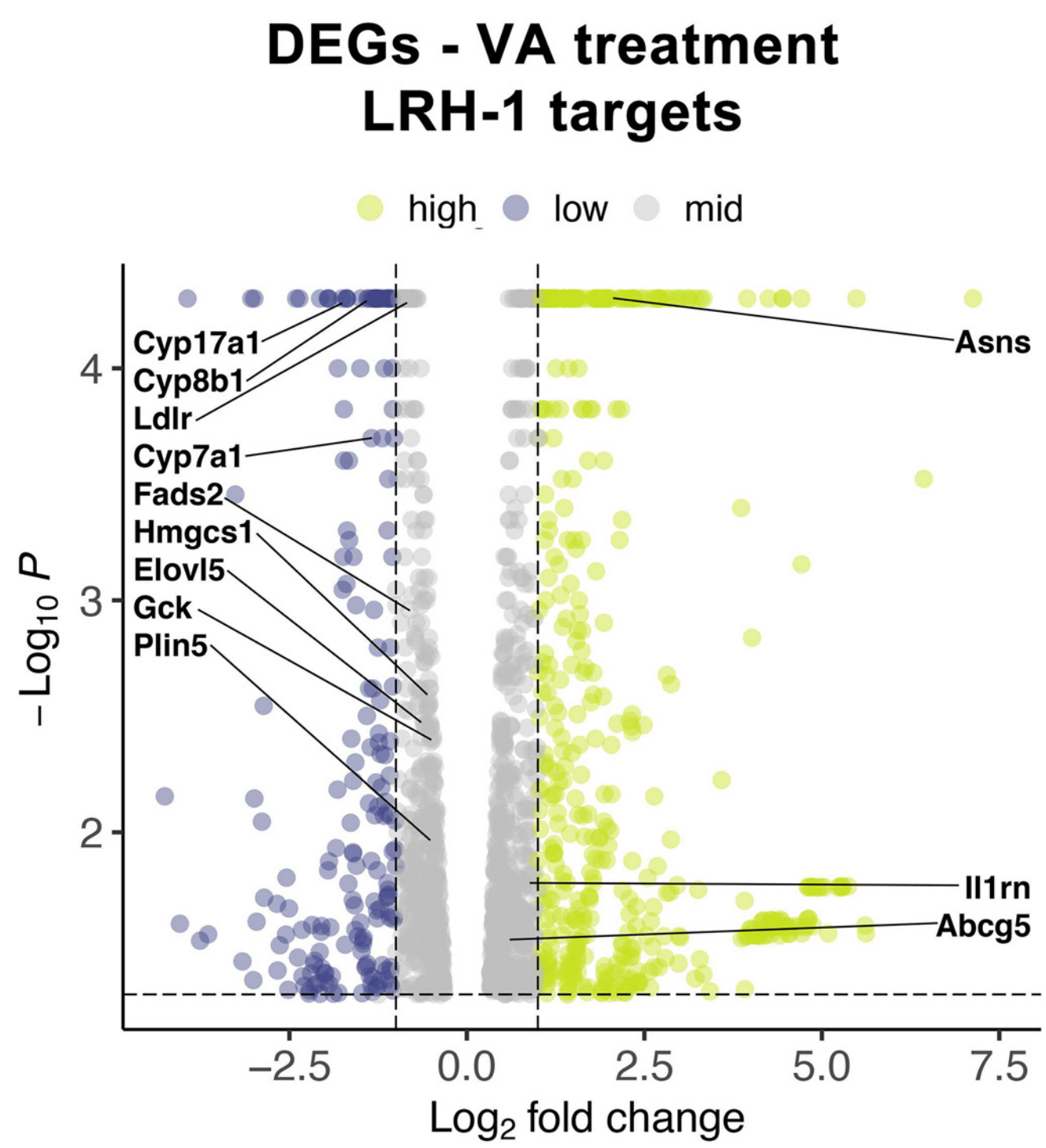

Figure EV8. (related to Fig. 6): VA modulates expression of different LRH-1 target genes.

Volcano plot of the differentially expressed genes (DEGs) in VA-treated HFD-fed mice compared to vehicle-treated mice. Annotated genes indicate established LRH-1 targets and are listed in Table EV1. Thresholds for significance were set at adjusted P value < 0.05 and log2 fold change of ±1 (low: P adj < −1, mid: −1<P adj<1, high: P adj>1).
