# Supplementary material for: Microbiome-host communication via vaccenic acid modulates LRH-1/NR5A2 activity and ameliorates metabolic liver disease
Source: EMBO Mol Med. 2026 Sep 17;18(10):4059–89. doi: 10.1038/s44321-026-00516-3 (PMC13658251; doi:10.1038/s44321-026-00516-3)

Appendix

Table of Content

Appendix Data 2-8
Appendix Figure S1 3
Appendix Figure S2 4
Appendix Figure S3 5
Appendix Figure S4 6
Appendix Figure S5 7
Appendix Figure S6 8
Appendix Figure S7 8

Appendix Data

^{1}H and ^{13}C spectra from fraction 1.5.3.47 (Appendix Figures S1-S2) were used for structure elucidation. Analysis of spectra and comparison with NMR data of commercial standards (Appendix Figures S3-S5) led to the identification of vaccenic acid.

Appendix Figure S1: ^{1}H NMR spectrum (600 MHz, $CDCl_3$)

Appendix Figure S2: ^{13}C NMR spectrum (151 MHz, $CDCl_3$)

Appendix Figure S3: HSQC NMR spectrum

Appendix Figure S4: COSY NMR spectrum

Appendix Figure S5: HMBC NMR spectrum

Appendix Figure S6: EI-MS spectrum

Appendix Figure S7: GC-MS spectrum

Appendix Figure S1: ^{1}H NMR spectrum (600 MHz, $CDCl_3$)

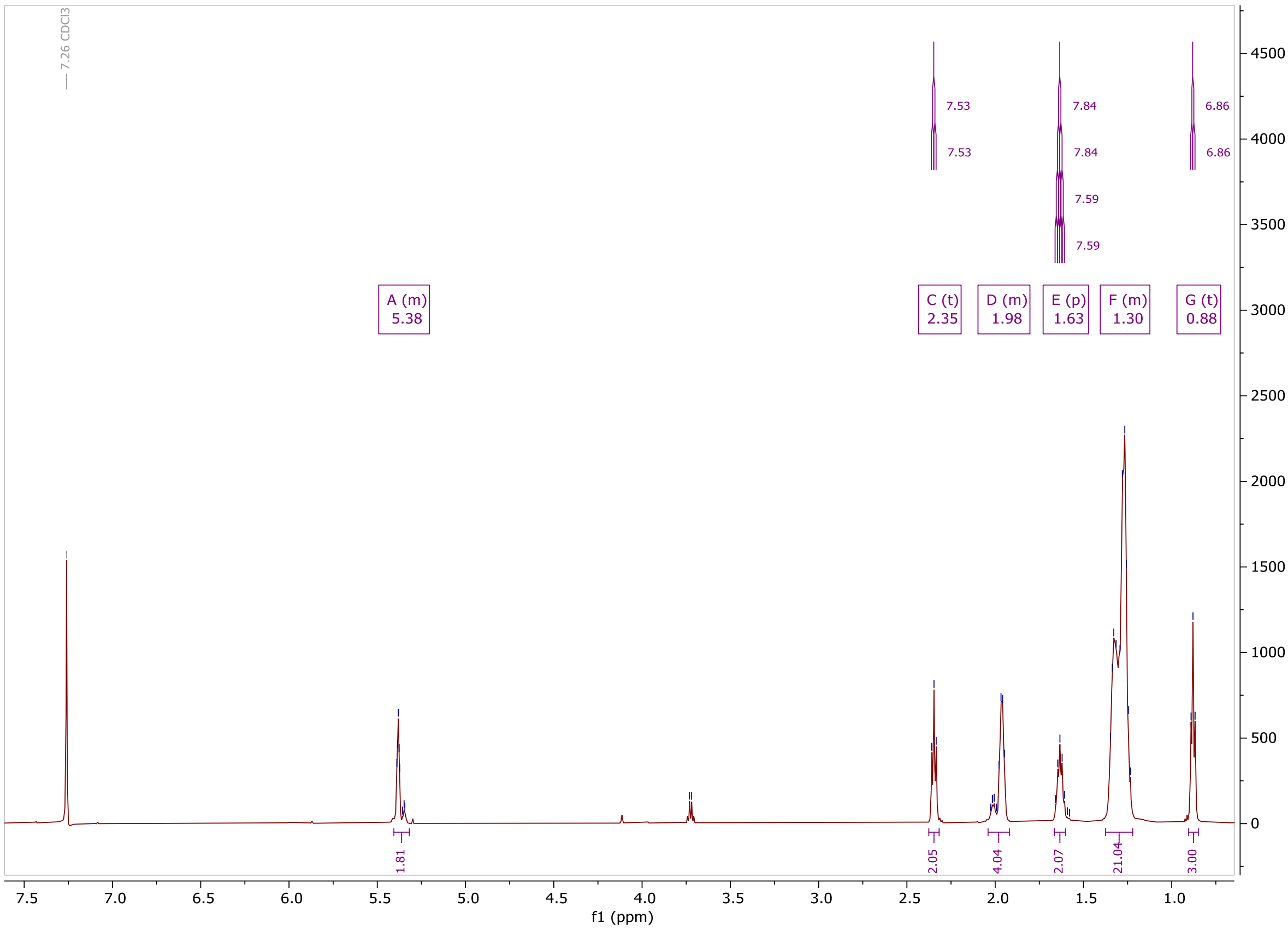

^{1}H NMR (600 MHz, $CDCl_3$) δ 5.43 – 5.33 (m, 2H), 2.35 (t, *J* = 7.5 Hz, 2H), 2.09 – 1.91 (m, 4H), 1.63 (p, *J* = 7.6 Hz, 2H), 1.34 (d, *J* = 6.4 Hz, 2H), 1.33 – 1.22 (m, 18H), 0.88 (t, *J* = 6.9 Hz, 3H).

Appendix Figure S2: ^{13}C NMR spectrum (151 MHz, $CDCl_3$)

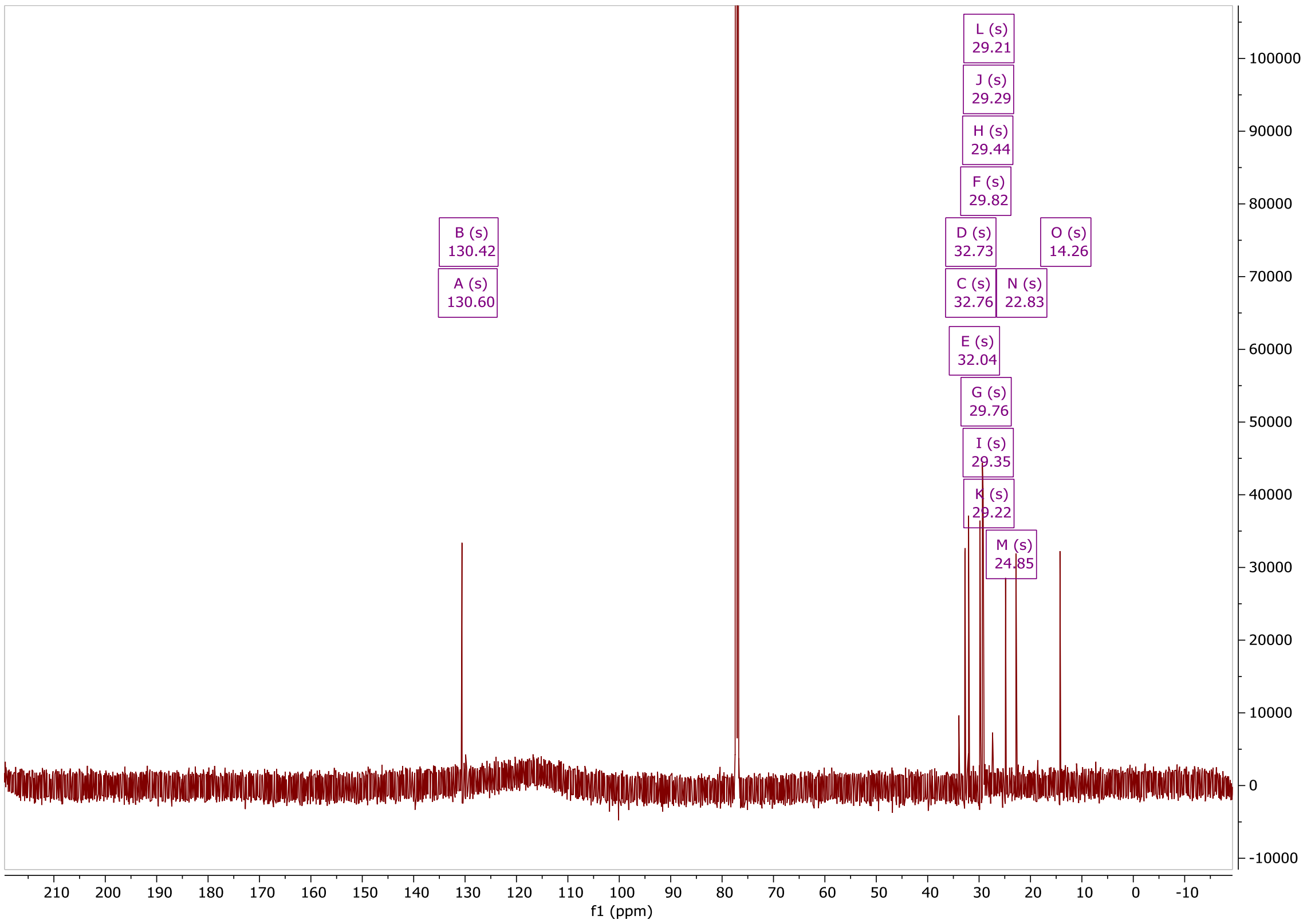

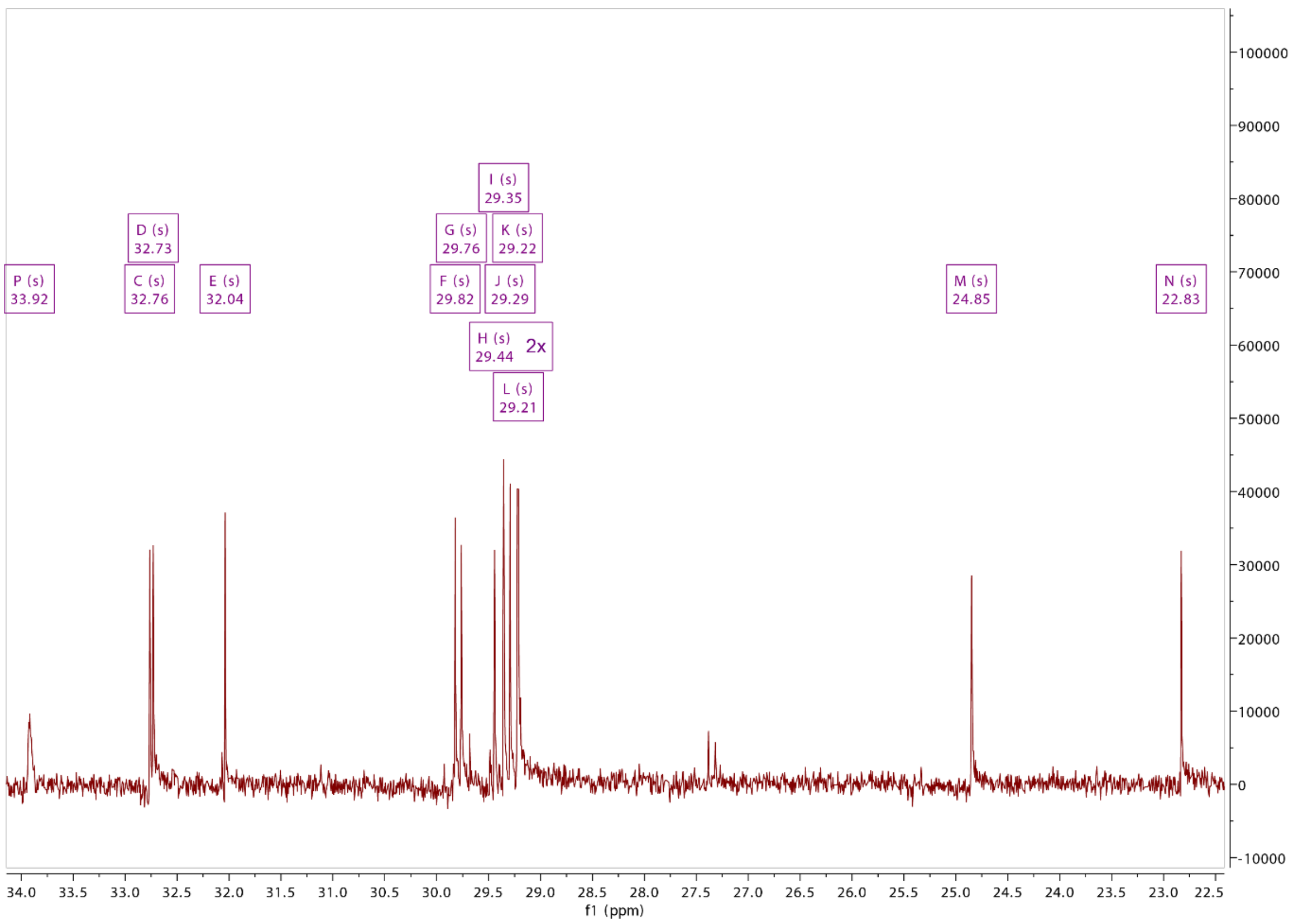

^{13}C NMR (151 MHz, CDCl3) δ 178.7 (derived from HMBC), 130.6, 130.4, 33.9, 32.8, 32.7, 32.0, 29.8, 29.8, 29.4, 29.4 (2x), 29.3, 29.2, 29.2, 24.8, 22.8, 14.3.

Appendix Figure S3: HSQC NMR spectrum

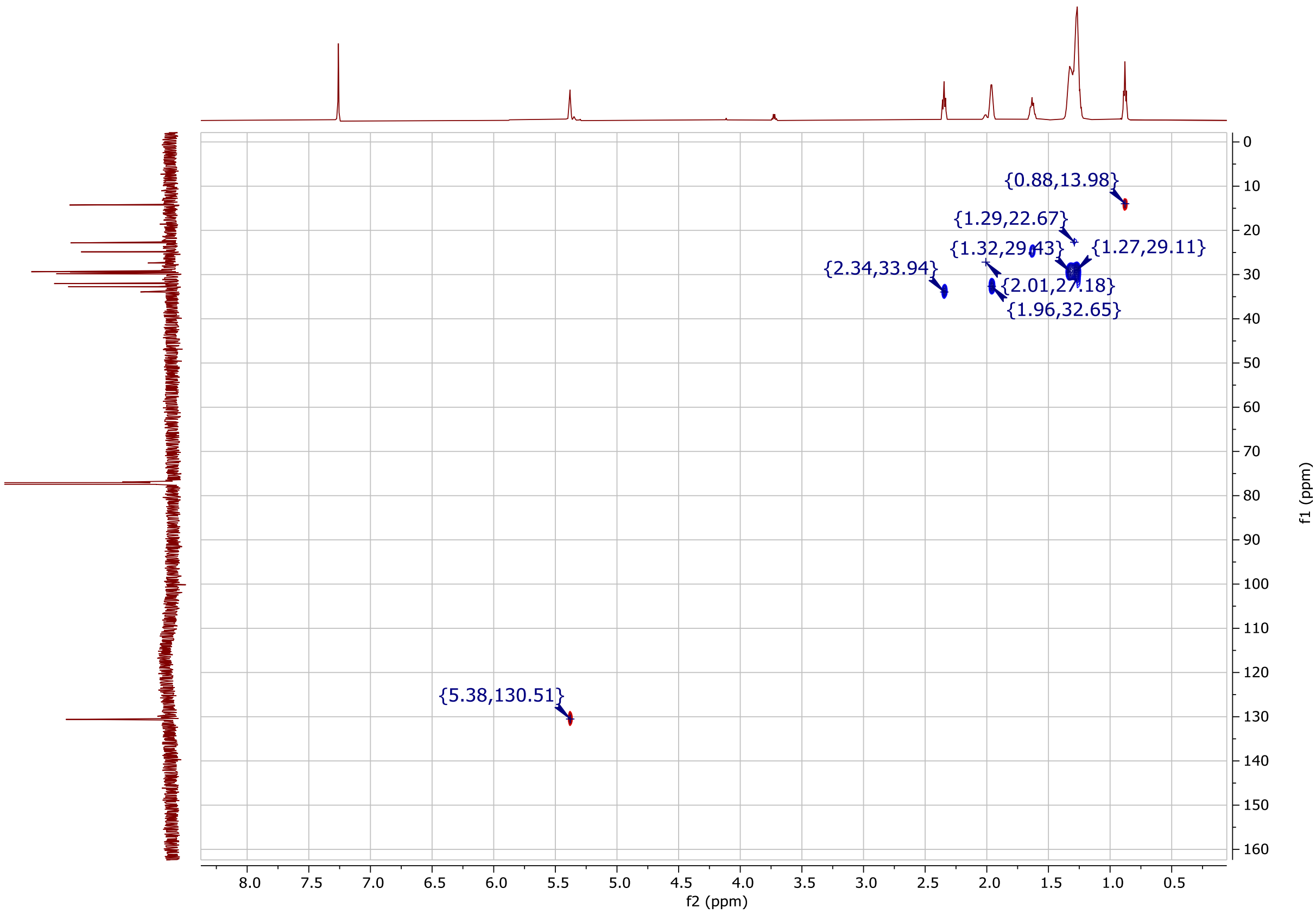

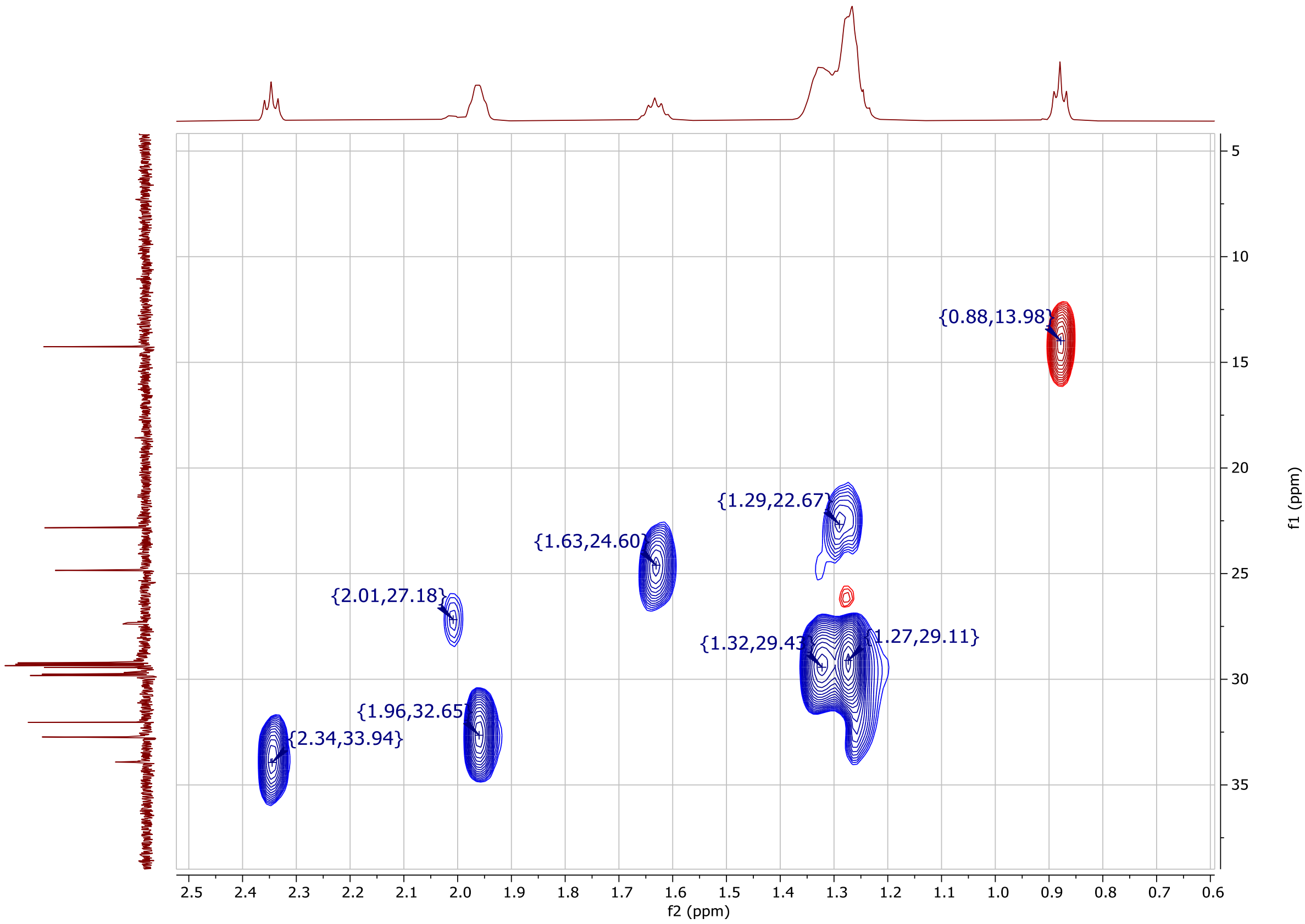

Appendix Figure S4: COSY NMR spectrum

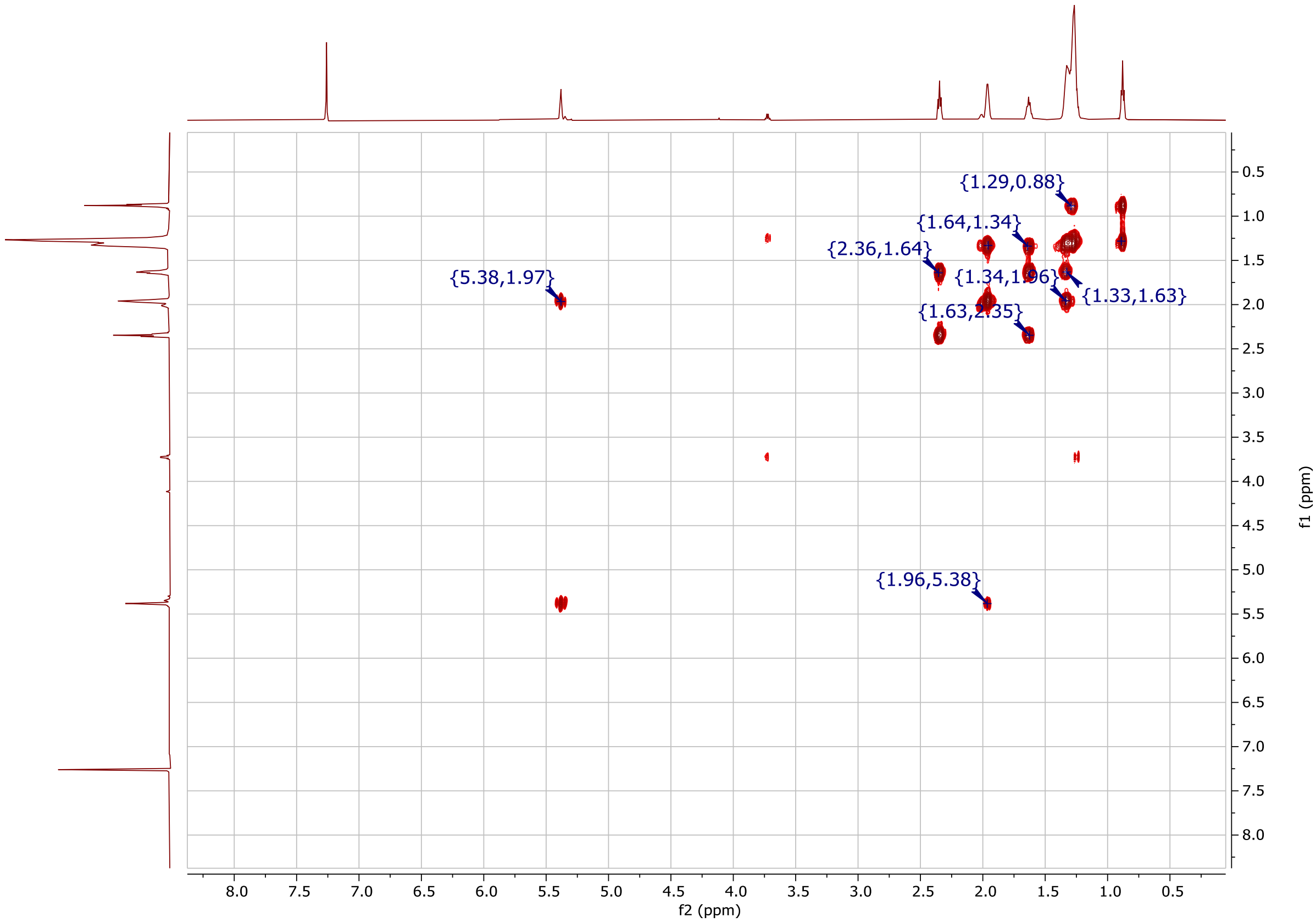

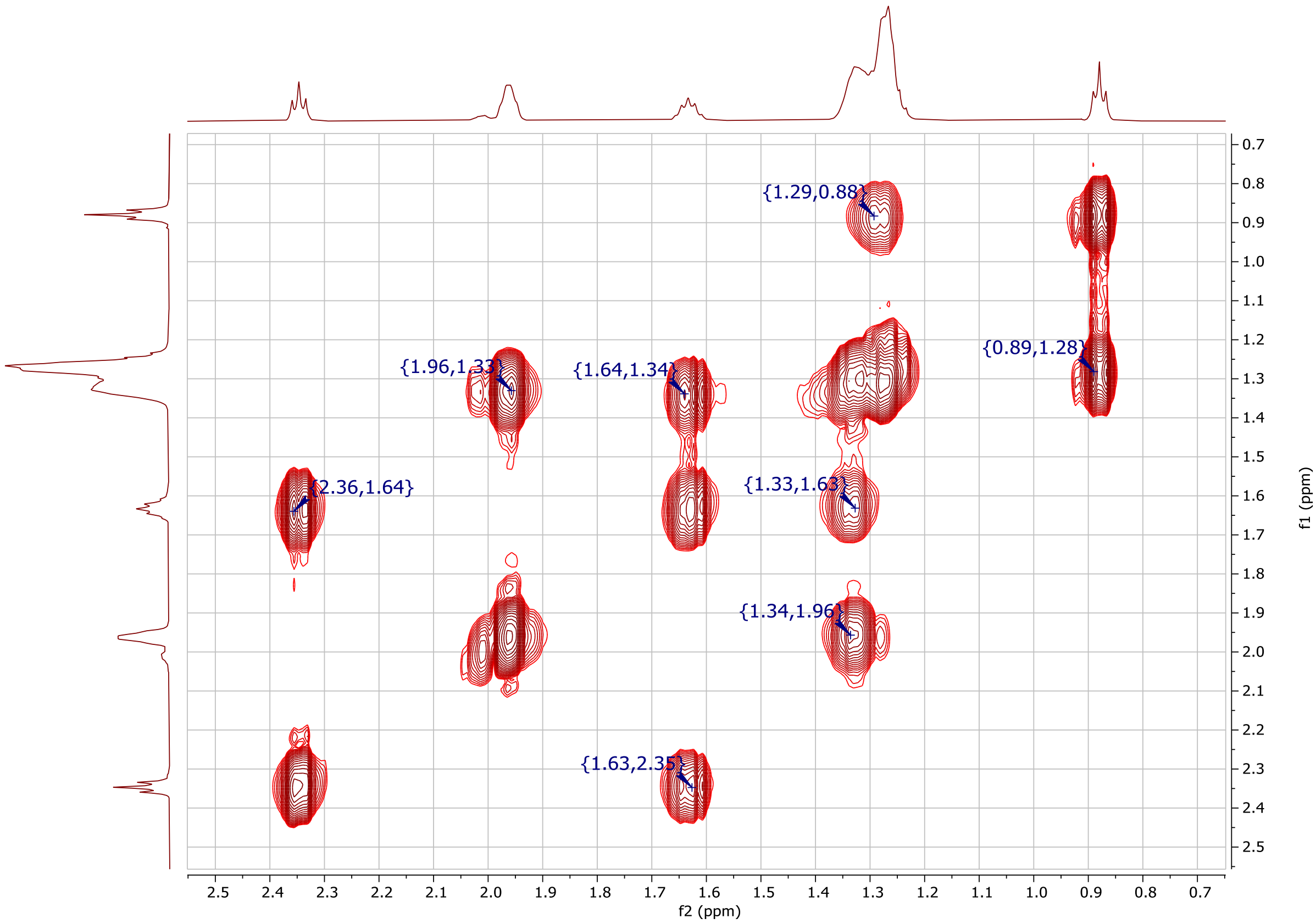

Appendix Figure S5: HMBC NMR spectrum

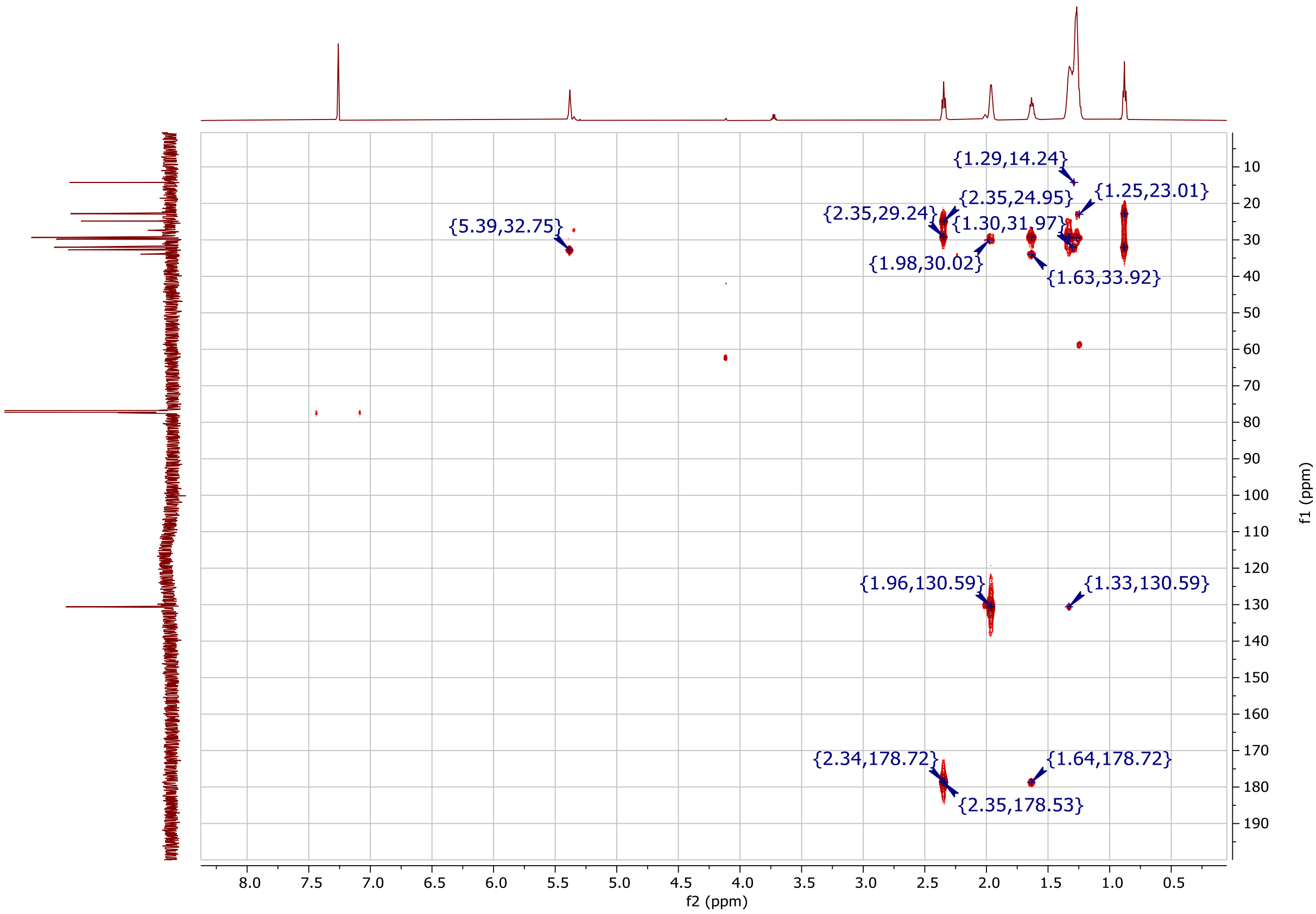

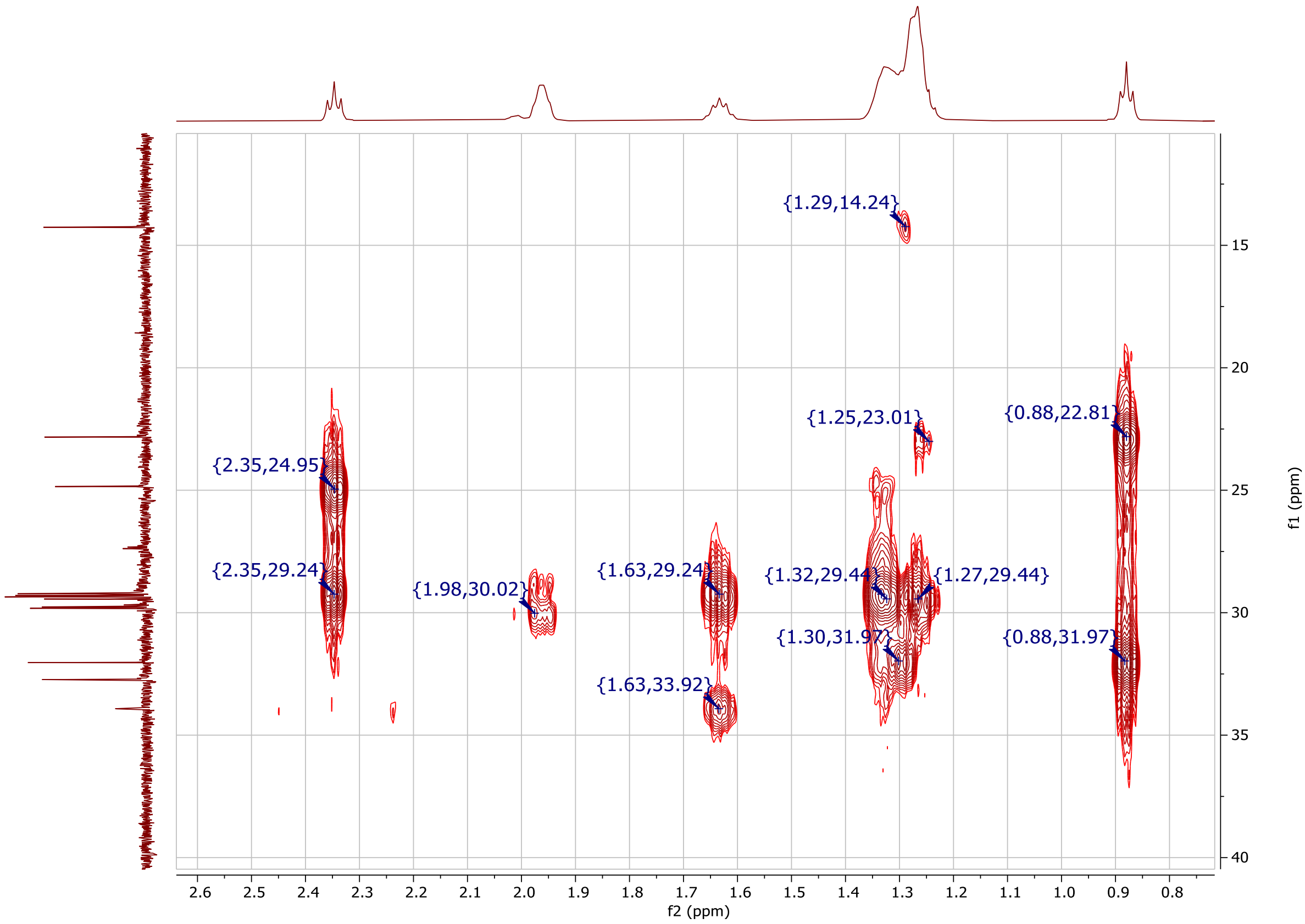

Appendix Figure S6: EI-MS spectrum (top panel) and Appendix Figure S7 (bottom panel): GC-MS spectrum

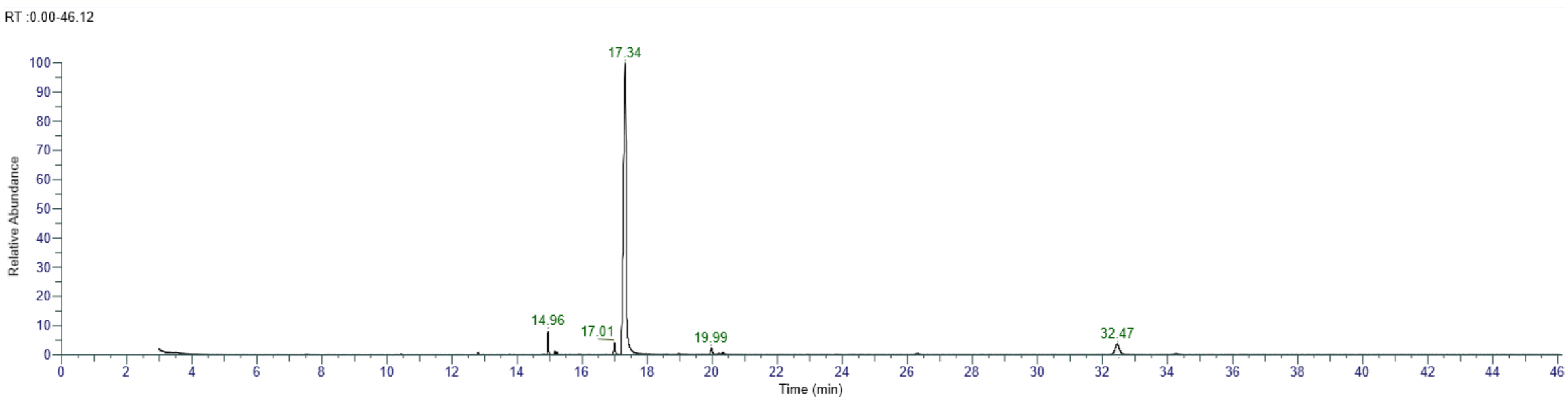

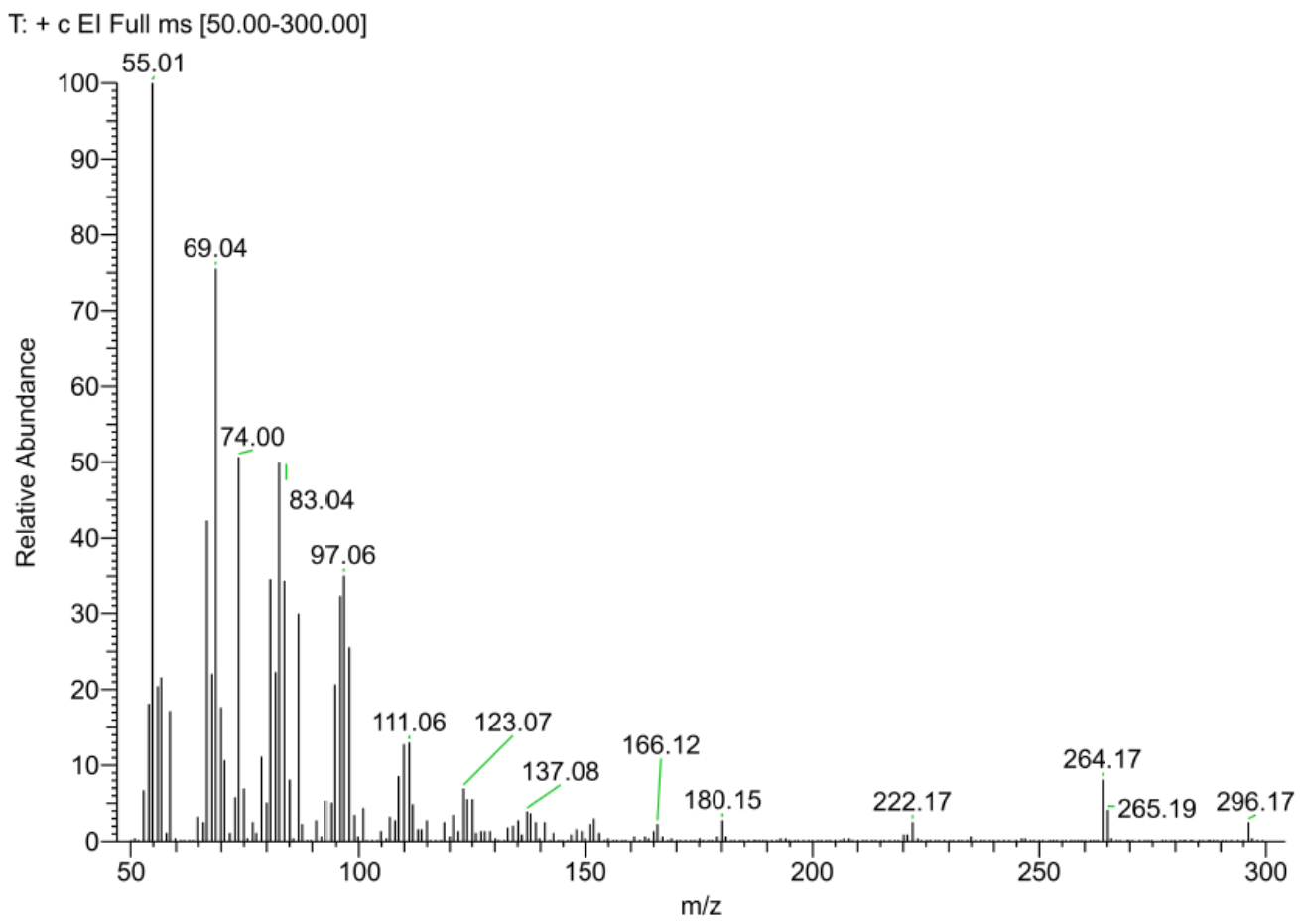

Supplement: Supplementary file 2 [file 44321_2026_516_MOESM2_ESM.pdf]
