# Supplementary material for: Microbiome-host communication via vaccenic acid modulates LRH-1/NR5A2 activity and ameliorates metabolic liver disease
Source: EMBO Mol Med. 2026 Sep 17;18(10):4059–89. doi: 10.1038/s44321-026-00516-3 (PMC13658251; doi:10.1038/s44321-026-00516-3)
Supplement: Supplementary file 3 [file 44321_2026_516_MOESM9_ESM.pdf]

EMBO Molecular Medicine

Microbiome-host communication via vaccenic acid modulates LRH-1/NR5A2 activity and ameliorates metabolic liver disease

Thomas Brunner, Anna Pia Plazzo, Verena Filz, Michaela Prothiwa, Rebekka Lambrecht, M Eugenia Delgado, Jennifer Fleming, Jerome Duschek, Elia Pilgram, Juliane Friedrich, Raphael Eisenring, Lea Kurtz, Kerstin Stemmer, Lotta Stenman, Fabian Amaya Garcia, Miriam Unterlass, Olga Mayans, and Thomas Böttcher

Corresponding author: Thomas Brunner (thomas.brunner@uni-konstanz.de)

Review Timeline:

Submission Date:	3rd Feb 26
Editorial Decision:	23rd Feb 26
Revision Received:	20th May 26
Editorial Decision:	3rd Jul 26
Revision Received:	13th Aug 26
Accepted:	20th Aug 26

Editor: Zeljko Durdevic

Transaction Report:

(Note: With the exception of the correction of typographical or spelling errors that could be a source of ambiguity, letters and reports are not edited. Depending on transfer agreements, referee reports obtained elsewhere may or may not be included in this compilation. Referee reports are anonymous unless the Referee chooses to sign their reports.)

23rd Feb 2026

Dear Prof. Brunner,

Thank you for the submission of your manuscript to EMBO Molecular Medicine. We have now received feedback from the three reviewers who agreed to evaluate your manuscript. All three referees recognize interest of the study but also raise importnat concerns that should be addressed in a major revision. If you would like to discuss further the points raised by the referees, I am available to do so via email or video. Let me know if you are interested in this option.

Further consideration of a revision that addresses reviewers' concerns in full will entail a second round of review. EMBO Molecular Medicine encourages a single round of revision only and therefore, acceptance or rejection of the manuscript will depend on the completeness of your responses included in the next, final version of the manuscript. For this reason, and to save you from any frustrations in the end, I would strongly advise against returning an incomplete revision.

We would welcome the submission of a revised version within three months for further consideration. Please let us know if you require longer to complete the revision.

I look forward to receiving your revised manuscript.

Yours sincerely,

Zeljko Durdevic

Zeljko Durdevic
Senior Editor
EMBO Molecular Medicine

*****

When preparing your revised manuscript, please refer to our guidelines: https://link.springer.com/journal/44321/submission-guidelines#cms-Revised-submissions. We perform an initial quality control of all revised manuscripts before re-review; failure to include requested items will delay the evaluation of your revision.

We require:

1) A .docx formatted version of the manuscript text (including legends for main figures, EV figures and tables). Please make sure that the changes are highlighted to be clearly visible.

2) Individual production quality figure files as .eps, .tif, .jpg (one file per figure). For guidance, download the 'Figure Guide PDF': https://media.springernature.com/original/springer-cms/rest/v1/content/27825798/data/v1.

3) A .docx formatted letter INCLUDING the reviewers' reports and your detailed point-by-point responses to their comments. As part of the EMBO Press transparent editorial process, the point-by-point response is part of the Review Process File (RPF), which will be published alongside your paper.

4) A complete author checklist, which you can download from our author guidelines. Please insert information in the checklist that is also reflected in the manuscript. The completed author checklist will also be part of the RPF.

5) Please note that all corresponding authors are required to supply an ORCID ID for their name upon submission of a revised manuscript.

6) It is mandatory to include a 'Data Availability' section after the Materials and Methods. Before submitting your revision, primary datasets produced in this study need to be deposited in an appropriate public database, and the accession numbers and database listed under 'Data Availability'. Please remember to provide a reviewer password if the datasets are not yet public.

In case you have no data that requires deposition in a public database, please state so in this section. Note that the Data Availability Section is restricted to new primary data that are part of this study.

7) For data quantification: please specify the name of the statistical test used to generate error bars and P values, the number (n) of independent experiments (specify technical or biological replicates) underlying each data point and the test used to calculate p-values in each figure legend. The figure legends should contain a basic description of n, P and the test applied. Graphs must include a description of the bars and the error bars (s.d., s.e.m.).

8) At EMBO Press we ask authors to provide source data for the main manuscript figures. You will receive a separate email with instructions for providing source data with your revised manuscript, including how to upload and organize the files.

9) Our journal encourages inclusion of *data citations in the reference list* to directly cite datasets that were re-used and obtained from public databases. Data citations in the article text are distinct from normal bibliographical citations and should directly link to the database records from which the data can be accessed. In the main text, data citations are formatted as follows: "Data ref: Smith et al, 2001" or "Data ref: NCBI Sequence Read Archive PRJNA342805, 2017". In the Reference list, data citations must be labeled with "[DATASET]". A data reference must provide the database name, accession number/identifiers and a resolvable link to the landing page from which the data can be accessed at the end of the reference.

10) We replaced Supplementary Information with Expanded View (EV) Figures and Tables that are collapsible/expandable online. A maximum of 5 EV Figures can be typeset. EV Figures should be cited as 'Figure EV1, Figure EV2" etc... in the text and their respective legends should be included in the main text after the legends of regular figures.

- For the figures that you do NOT wish to display as Expanded View figures, they should be bundled together with their legends in a single PDF file called *Appendix*, which should start with a short Table of Content. Appendix figures should be referred to in the main text as: "Appendix Figure S1, Appendix Figure S2" etc.

- Additional Tables/Datasets should be labeled and referred to as Table EV1, Dataset EV1, etc. Legends have to be provided in a separate tab in case of .xls files. Alternatively, the legend can be supplied as a separate text file (README) and zipped together with the Table/Dataset file.

11) The paper explained: EMBO Molecular Medicine articles are accompanied by a summary of the articles to emphasize the major findings in the paper and their medical implications for the non-specialist reader. Please provide a draft summary of your article highlighting

- the medical issue you are addressing,

- the results obtained and

- their clinical impact.

This may be edited to ensure that readers understand the significance and context of the research. Please refer to any of our published articles for an example.

12) Author contributions: You will be asked to provide CRediT (Contributor Role Taxonomy) terms in the submission system. These replace a narrative author contribution section in the manuscript.

13) A Conflict of Interest statement should be provided in the main text.

14) Every published paper includes a 'Synopsis' to further enhance discoverability. Synopses are displayed on the journal webpage and are freely accessible to all readers. They include a short stand first (maximum of 300 characters, including space) as well as 2-5 one-sentences bullet points that summarizes the paper. Please write the bullet points to summarize the key NEW findings. They should be designed to be complementary to the abstract - i.e. not repeat the same text. We encourage inclusion of key acronyms and quantitative information (maximum of 30 words / bullet point). Please use the passive voice. Please attach these in a separate file or send them by email, we will incorporate them accordingly.

Please also suggest a striking image or visual abstract to illustrate your article as a PNG file 550 px wide x 300-600 px high.

15) Include a Reagents and Tools Table as part of the Methods section, which can be downloaded from our author guidelines.

EMBO Molecular Medicine has a "scooping protection" policy, whereby similar findings that are published by others during review or revision are not a criterion for rejection. Should you decide to submit a revised version, I do ask that you get in touch after three months if you have not completed it, to update us on the status.

***** Reviewer's comments *****

Referee #1 (Remarks for Author):

The authors present a well-designed and logically structured study addressing the role of microbiota-derived vaccenic acid as a modulator of LRH-1 activity and metabolic homeostasis. The manuscript integrates biochemical, cellular and in vivo experiments in a coherent and progressive manner, moving from the identification of bacterial lipid activity to functional characterization in diet-induced obese mice. Nevertheless, some considerations should be made:

- Since vaccenic acid was shown to stimulate transcriptional activity of PPARα in vitro (Fig EV4E) and given the established role of PPARα in regulating hepatic fatty acid oxidation and lipogenesis, it would be important to better delineate the relative contribution of PPARα to the observed hepatic transcriptional changes. Reporting canonical PPARα target genes such as Acox1, Cpt1a, Hmgcs2, Fgf21 (Kersten et al., 2014) from the existing RNA-seq dataset would help to clarify whether the metabolic improvements and suppression of lipogenic genes are exclusively related to LRH-1 activation or may involve parallel PPARα signalling. In addition, the quantification of hepatic Fgf21 expression and circulating FGF21 levels would serve as robust functional indicator of PPARα activation in vivo, as FGF21 is a well-established PPARα target and circulating hepatokine.
- The downregulation of Cyp7a1 and Cyp8b1 following VA treatment is intriguing, as these genes are subject to complex regulation by the FXR-SHP-FGF15 axis and are strongly influenced by bile acid status and nutritional state. To better contextualize these findings, the authors are encouraged to clarify the feeding/fasting conditions and timing of tissue collection. If available, reporting hepatic SHP and intestinal Fgf15 expression would further help determine whether FXR signaling contributes to the observed regulation of bile acid synthesis genes.
- The authors show that bacterial lipid extracts fail to activate the LRH-1 LBP mutant (F342W/I416W) (Fig 1D), supporting ligand-dependent activation. However, it is not reported whether VA was tested on this mutant receptor. Since VA was evaluated in the LRH-1 reporter assay (Fig 2F), it would be important to confirm in parallel that VA does not activate the F342W/I416W mutant, which was already used to validate bacterial extract-mediated activation. Demonstrating reduced or absent responsiveness of the mutant to pure VA would further strengthen the conclusion that VA directly engages the ligand-binding pocket of LRH-1.
- The authors report that cis-VA is detected in multiple bacterial species, whereas trans-VA appears to be specific to B420, and that the bioactive fraction contained both isomers. However, the in vivo experiments were performed using a 1:1 mixture of cis- and trans-VA. Given that cis-VA displays a markedly higher binding affinity to LRH-1 compared to trans-VA, the relative proportion of the two isomers may influence the overall biological effect. It would therefore be helpful to clarify the rationale for selecting a 1:1 mixture and whether this reflects the relative abundance of the isomers in the active bacterial extract. Providing this information would improve transparency and strengthen the physiological interpretation of the in vivo data.
- The Discussion mentions that physiological concentrations of VA in humans are in the micromolar range. It would be helpful to briefly relate this information to the dosing used in the present in vivo experiments (50 mg/kg i.p.), and to discuss whether comparable micromolar concentrations are expected to be achieved in plasma or liver under these conditions.
- The number of animals included in each experimental group should be clearly indicated in the figure legends or Methods section. Providing the exact n per group is essential to assess statistical power and interpret the robustness of the reported metabolic and histological outcomes.

Referee #2 (Remarks for Author):

This elegant study, led by the Brunner group in collaboration with several international teams, reports the identification of vaccenic acid, a metabolite derived from intestinal bacteria, as a novel LRH-1/NR5A2 agonist implicated in the regulation of hepatic metabolism. The authors employ a broad and complementary range of approaches, spanning in vitro metabolite screening and mechanistic characterization to in vivo metabolic analyses in multiple mouse models. The study is carefully designed and rigorously executed, with potential caveats thoughtfully addressed and the conclusions supported by a strong and coherent experimental framework. I only have a few comments:

In Figure EV1 and 1, please provide rationale of using the epithelial-like LS174T cell line established from the colon of a colorectal adenocarcinoma rather than a hepatic-derived cell line.

In Figure EV2, please clarify why Caco-2 cells rather than the LS174T cells with the inducible LRH-1 construct.

In Figure 3D and E the authors shown that the Kd of trans- VA is 10 X higher that for cis-VA (144.4 uM 14.2) yet in Figure 2F both cis and trans-VA display the same capacity for activating LRH-1. Could they provide an explanation as one would expect less activation potential for trans-VA.

In vivo administration of VA to HFD as well as to SD fed mice resulted in decreased glucose serum levels (Figure 5D). Yet insulin levels were only decreased in the HFD group (Figure 5E) suggesting that lowering of glycemia was not due to improved glucose tolerance by peripheral tissues but rather improved live function (which would make sense), albeit the RNAseq did not reveal any genes involved in glucose metabolism (Figure 6A). Did the authors perform any oral glucose (or insulin) tolerance test to rule out the role of other peripheral tissues such as muscle?

Figure 6A and its description in the Results section are unclear. The dot plot displays Gene Ratio values, which represent enrichment magnitude but do not convey directionality. The term "VA-induced" implies pathway activation; however, it is not specified whether these pathways are enriched among upregulated genes, downregulated genes, or the full gene set. The authors should clarify the gene list used for enrichment and revise the terminology accordingly.

Referee #3 (Remarks for Author):

In this manuscript, Plazzo et al present new evidence for a microbially produced modulator of LRH-1 activity with impact on hepatic steatosis and glucose homeostasis. This work brings new insight into LRH-1 ligand binding while linking receptor activity with host-microbiome communication. The findings are impactful in that they 1) establish fatty acids (FA) as a novel ligand for this orphan receptor, 2) provide a new mechanism that links a microbially-produced FA to host metabolism, 3) supports FA ligand binding by the murine LRH-1 isoform, and 4) adds to the growing literature supporting the therapeutic targeting of LRH-1 for metabolic disorders including MASLD.

Strengths of this work include the breadth of study from biochemical analysis of FA binding to the LRH-1 LBD through in vitro and in vivo studies using mouse and human receptor isoforms. In particular, the use of the LRH-1 LBD double mutant supports the VA binding within LRH-1's binding pocket. Specificity is further supported through the use of inducible LRH-1 expression cell models, analysis of non-VA microbially produced FAs, and use of MST to demonstrate physical association of VA with the LRH-1 LBD. Together, this work supports the conclusion that VA agonizes LRH-1 in cellular and animal contexts with physiological consequences.

Minor suggestions/concerns for the study are as follows:
LRH-1 biochemical specificity. In the MST assay, data are provided for VA and a panel of FA associating with LRH-1 LBD. Parallel experiments with the mutant LRH-1 LBD would provide further support for specific LRH-1-VA binding in the LBD binding pocket.

Hepatic LRH-1 engagement in vivo. The authors show improvements in hepatic steatosis, blood glucose, and insulin in HFD model with C57/Bl6 mice. The glucose measurements, however, are provided at a single time point. Glucose and insulin tolerance tests could be considered to better define the impact of VA treatment on glucose homeostasis. Additionally, use of the group's Lrh1-flox mice with AAV-delivered Cre (as done in Miranda et al JCI Insights 2018) would further validate in vivo hepatic LRH-1 engagement.

Hepatic Steatosis. Liver histology provided in Fig. 5 is reminiscent of Miranda et al JCI Insights 2018 findings describing LRH-1-mediated lipid droplet remodeling related to Elovl5 and Fads2 expression. These genes are labeled in EV8 but the relationship to the current study should be discussed in the main text.

LRH-1 Target Gene Expression. Gene targets for LRH-1 are assessed and found to variable, which may relate to how specifically VA activates LRH-1 or impacts co-factor recruitment. The clear decrease in Cyp7a1 and Cyp8b1, however, raises the possibility that LRH-1 agonism broadly impacts bile acids that feed-back on these Cyp genes. Have the authors measured total bile acids?

***** Reviewer's comments *****

Referee #1 (Remarks for Author):

The authors present a well-designed and logically structured study addressing the role of microbiota-derived vaccenic acid as a modulator of LRH-1 activity and metabolic homeostasis. The manuscript integrates biochemical, cellular and in vivo experiments in a coherent and progressive manner, moving from the identification of bacterial lipid activity to functional characterization in diet-induced obese mice. Nevertheless, some considerations should be made:

- Since vaccenic acid was shown to stimulate transcriptional activity of PPARα in vitro (Fig EV4E) and given the established role of PPARα in regulating hepatic fatty acid oxidation and lipogenesis, it would be important to better delineate the relative contribution of PPARα to the observed hepatic transcriptional changes. Reporting canonical PPARα target genes such as Acox1, Cpt1a, Hmgcs2, Fgf21 (Kersten et al., 2014) from the existing RNA-seq dataset would help to clarify whether the metabolic improvements and suppression of lipogenic genes are exclusively related to LRH-1 activation or may involve parallel PPARα signalling. In addition, the quantification of hepatic Fgf21 expression and circulating FGF21 levels would serve as robust functional indicator of PPARα activation in vivo, as FGF21 is a well-established PPARα target and circulating hepatokine.

- The downregulation of Cyp7a1 and Cyp8b1 following VA treatment is intriguing, as these genes are subject to complex regulation by the FXR-SHP-FGF15 axis and are strongly influenced by bile acid status and nutritional state. To better contextualize these findings, the authors are encouraged to clarify the feeding/fasting conditions and timing of tissue collection. If available, reporting hepatic SHP and intestinal Fgf15 expression would further help determine whether FXR signaling contributes to the observed regulation of bile acid synthesis genes.

- The authors show that bacterial lipid extracts fail to activate the LRH-1 LBP mutant (F342W/I416W) (Fig 1D), supporting ligand-dependent activation. However, it is not reported whether VA was tested on this mutant receptor. Since VA was evaluated in the LRH-1 reporter assay (Fig 2F), it would be important to confirm in parallel that VA does not activate the F342W/I416W mutant, which was already used to validate bacterial extract-mediated activation. Demonstrating reduced or absent responsiveness of the mutant to pure VA would further strengthen the conclusion that VA directly engages the ligand-binding pocket of LRH-1.

- The authors report that cis-VA is detected in multiple bacterial species, whereas trans-VA appears to be specific to B420, and that the bioactive fraction contained both isomers. However, the in vivo experiments were performed using a 1:1 mixture of cis- and trans-VA. Given that cis-VA displays a markedly higher binding affinity to LRH-1 compared to trans-VA, the relative proportion of the two isomers may influence the overall biological effect. It would therefore be helpful to clarify the rationale for selecting a 1:1 mixture and whether

this reflects the relative abundance of the isomers in the active bacterial extract. Providing this information would improve transparency and strengthen the physiological interpretation of the in vivo data.

- The Discussion mentions that physiological concentrations of VA in humans are in the micromolar range. It would be helpful to briefly relate this information to the dosing used in the present in vivo experiments (50 mg/kg i.p.), and to discuss whether comparable micromolar concentrations are expected to be achieved in plasma or liver under these conditions.

- The number of animals included in each experimental group should be clearly indicated in the figure legends or Methods section. Providing the exact n per group is essential to assess statistical power and interpret the robustness of the reported metabolic and histological outcomes.

Referee #2 (Remarks for Author):

This elegant study, led by the Brunner group in collaboration with several international teams, reports the identification of vaccenic acid, a metabolite derived from intestinal bacteria, as a novel LRH-1/NR5A2 agonist implicated in the regulation of hepatic metabolism. The authors employ a broad and complementary range of approaches, spanning in vitro metabolite screening and mechanistic characterization to in vivo metabolic analyses in multiple mouse models. The study is carefully designed and rigorously executed, with potential caveats thoughtfully addressed and the conclusions supported by a strong and coherent experimental framework. I only have a few comments:

In Figure EV1 and 1, please provide rationale of using the epithelial-like LS174T cell line established from the colon of a colorectal adenocarcinoma rather than a hepatic-derived cell line.

In Figure EV2, please clarify why Caco-2 cells rather than the LS174T cells with the inducible LRH-1 construct.

In Figure 3D and E the authors shown that the Kd of trans- VA is 10 X higher that for cis-VA (144.4 uM 14.2) yet in Figure 2F both cis and trans-VA display the same capacity for activating LRH-1. Could they provide an explanation as one would expect less activation potential for trans-VA.

In vivo administration of VA to HFD as well as to SD fed mice resulted in decreased glucose serum levels (Figure 5D). Yet insulin levels were only decreased in the HFD group (Figure 5E) suggesting that lowering of glycemia was not due to improved glucose tolerance by peripheral tissues but rather improved live function (which would make sense), albeit the RNAseq did not reveal any genes involved in glucose metabolism (Figure 6A). Did the authors perform any oral glucose (or insulin) tolerance test to rule out the role of other peripheral tissues such as muscle?

Figure 6A and its description in the Results section are unclear. The dot plot displays Gene Ratio values, which represent enrichment magnitude but do not convey directionality. The term "VA-induced" implies pathway activation; however, it is not specified whether these pathways are enriched among upregulated genes, downregulated genes, or the full gene set. The authors should clarify the gene list used for enrichment and revise the terminology accordingly.

Referee #3 (Remarks for Author):

In this manuscript, Plazzo et al present new evidence for a microbially produced modulator of LRH-1 activity with impact on hepatic steatosis and glucose homeostasis. This work brings new insight into LRH-1 ligand binding while linking receptor activity with host-microbiome communication. The findings are impactful in that they 1) establish fatty acids (FA) as a novel ligand for this orphan receptor, 2) provide a new mechanism that links a microbially-produced FA to host metabolism, 3) supports FA ligand binding by the murine LRH-1

isoform, and 4) adds to the growing literature supporting the therapeutic targeting of LRH-1 for metabolic disorders including MASLD.

Strengths of this work include the breadth of study from biochemical analysis of FA binding to the LRH-1 LBD through in vitro and in vivo studies using mouse and human receptor isoforms. In particular, the use of the LRH-1 LBD double mutant supports the VA binding within LRH-1's binding pocket. Specificity is further supported through the use of inducible LRH-1 expression cell models, analysis of non-VA microbially produced FAs, and use of MST to demonstrate physical association of VA with the LRH-1 LBD. Together, this work supports the conclusion that VA agonizes LRH-1 in cellular and animal contexts with physiological consequences.

Minor suggestions/concerns for the study are as follows:
LRH-1 biochemical specificity. In the MST assay, data are provided for VA and a panel of FA associating with LRH-1 LBD. Parallel experiments with the mutant LRH-1 LBD would provide further support for specific LRH-1-VA binding in the LBD binding pocket.

Hepatic LRH-1 engagement in vivo. The authors show improvements in hepatic steatosis, blood glucose, and insulin in HFD model with C57/Bl6 mice. The glucose measurements, however, are provided at a single time point. Glucose and insulin tolerance tests could be considered to better define the impact of VA treatment on glucose homeostasis. Additionally, use of the group's Lrh1-flox mice with AAV-delivered Cre (as done in Miranda et al JCI Insights 2018) would further validate in vivo hepatic LRH-1 engagement.

Hepatic Steatosis. Liver histology provided in Fig. 5 is reminiscent of Miranda et al JCI Insights 2018 findings describing LRH-1-mediated lipid droplet remodeling related to Elovl5 and Fads2 expression. These genes are labeled in EV8 but the relationship to the current study should be discussed in the main text.

LRH-1 Target Gene Expression. Gene targets for LRH-1 are assessed and found to variable, which may relate to how specifically VA activates LRH-1 or impacts co-factor recruitment. The clear decrease in Cyp7a1 and Cyp8b1, however, raises the possibility that LRH-1 agonism broadly impacts bile acids that feed-back on these Cyp genes. Have the authors measured total bile acids?

Authors´ Response to Reviewers

All responses are marked in BLUE

We are very grateful to all of the reviewers for their very positive comments and constructive criticism on our manuscript (EMM-2026-23418) titled "**Microbiome-host communication via vaccenic acid modulates LRH-1/NR5A2-regulated liver metabolism**". We appreciate also the important suggestions, which helped to considerably improve the quality of our revised manuscript. We have incorporated many of these suggestions, as well as new experimental data, into the revised manuscript. A point-to-point reply to all inquiries is presented below.

Referee #1 (Remarks for Author):

The authors present a well-designed and logically structured study addressing the role of microbiota-derived vaccenic acid as a modulator of LRH-1 activity and metabolic homeostasis. The manuscript integrates biochemical, cellular and in vivo experiments in a coherent and progressive manner, moving from the identification of bacterial lipid activity to functional characterization in diet-induced obese mice. Nevertheless, some considerations should be made:

Answer: We thank the reviewer for both the positive comments and for raising important points that should be integrated and discussed in the manuscript.

- Since vaccenic acid was shown to stimulate transcriptional activity of PPARα in vitro (Fig EV4E) and given the established role of PPARα in regulating hepatic fatty acid oxidation and lipogenesis, it would be important to better delineate the relative contribution of PPARα to the observed hepatic transcriptional changes. Reporting canonical PPARα target genes such as Acox1, Cpt1a, Hmgcs2, Fgf21 (Kersten et al., 2014) from the existing RNA-seq dataset would help to clarify whether the metabolic improvements and suppression of lipogenic genes are exclusively related to LRH-1 activation or may involve parallel PPARα signalling.

Answer: We thank the reviewer for this important suggestion. We have now analyzed our RNAseq dataset for PPARα targets in general, and Acox1, Cpt1a, Hmgcs2, Fgf21 as recommended specifically. While the general bioinformatical analysis of the RNA sequencing revealed that many PPARα targets might indeed be induced by VA, more detailed analysis of Acox1, Cpt1a, Hmgcs2, Fgf21 expression confirmed only a significant induction of Fgf21. This significant induction is, however, mostly due to a single very high expression value (Figure 1). More detailed analysis of Fgf21 expression by qRT-PCR failed to confirm this induction (Figure 2).

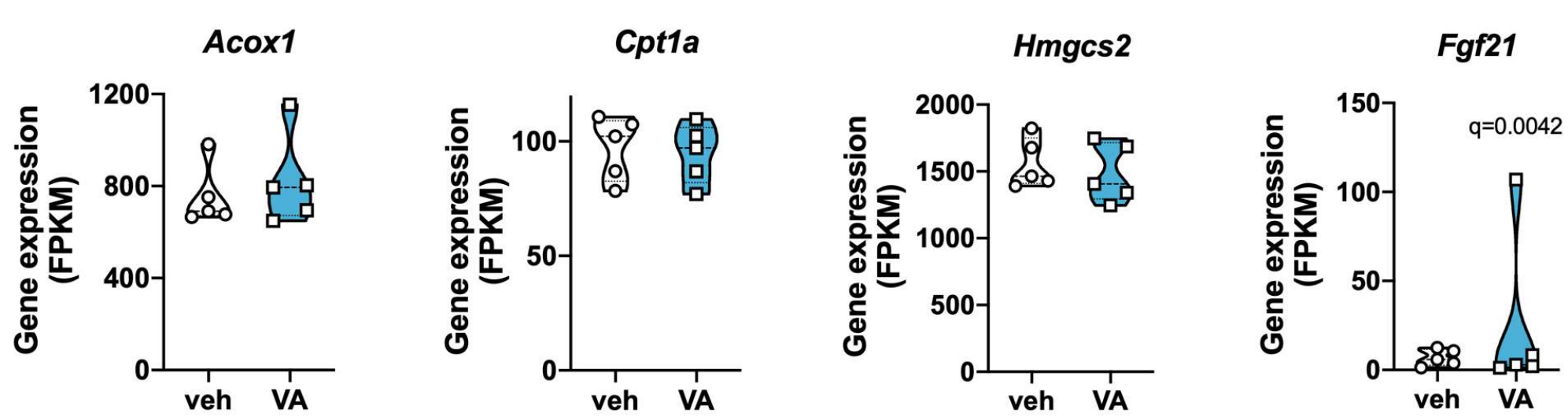

***Figure 1:** Analysis VA-induced expression of the PPARα targets Acox1, Cpt1a, Hmgcs2, Fgf21 in liver samples by RNA sequencing. q: FDR-adjusted p-value (multiple testing correction).*

In addition, the quantification of hepatic Fgf21 expression and circulating FGF21 levels would serve as robust functional indicator of PPARα activation in vivo, as FGF21 is a well-established PPARα target and circulating hepatokine.

Answer: In addition to reanalyzing our RNA seq data set, we have now also analyzed the Fgf21 expression in liver tissue 4 h (left panel) and 18 h (right panel) after injection of VA or its vehicle control by RT-qPCR. As can be seen in Figure 2, although we observed a trend for increased Fgf21 expression in VA-treated liver samples after 4 h, this could not be confirmed at 18 h. Thus, although we cannot exclude that VA administration may also induce PPARα-regulated processes in vivo, the quantitative analysis of PPARα target genes did not support a major contribution of PPARα in the observed VA-induced effects on liver metabolism.

Since these analyses of PPARα target genes resulted in negative findings, which did not alter the key messages of our study, we decided to show these results here for review only and not to include them in the revised manuscript, also due to space limitations.
Unfortunately, we had been using up most of our serum samples generated in the *in vivo* experiments for the various analyses reported in our manuscript. Thus, only few microliters remained in the storage tubes, which freeze-dried during storage at -80°C. Thus, analysis of Fgf21 protein levels by ELISA was not possible.

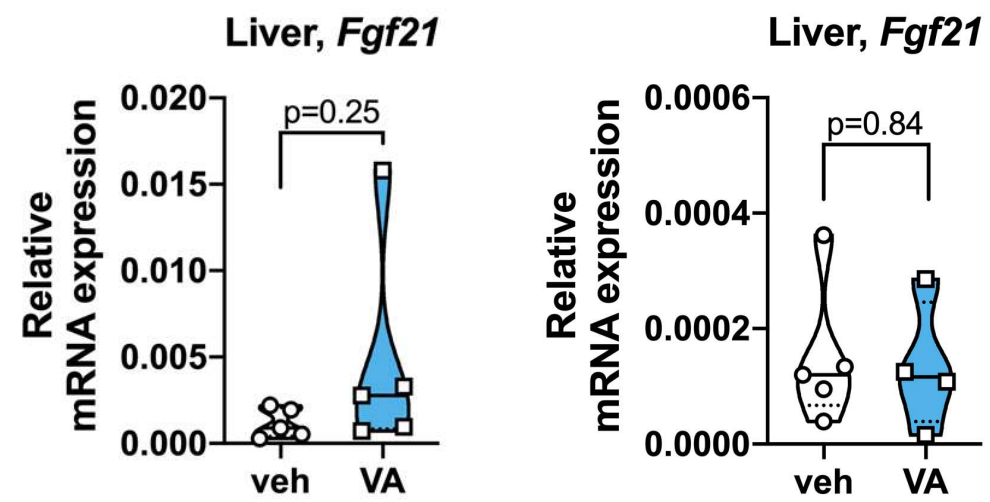

***Figure 2:** Quantitative RT-PCR analysis of Fgf21 expression the liver at 4 h (left panel) and 18 h (right panel) after vehicle or VA administration.*

- The downregulation of Cyp7a1 and Cyp8b1 following VA treatment is intriguing, as these genes are subject to complex regulation by the FXR-SHP-FGF15 axis and are strongly influenced by bile acid status and nutritional state. To better contextualize these findings, the authors are encouraged to clarify the feeding/fasting conditions and timing of tissue collection.

Answer: We agree with the reviewer that downregulation of bile acid-regulating Cyp7a1 and Cyp8b1 is very interesting. Although these are established LRH-1 target genes, stimulation of LRH-1 activity by different ligands was reported to result in opposite outcomes, where expression levels of Cyp7a1 and Cyp8b1 are either up- or downregulated. We agree that clarifying the feeding/fasting conditions and timing of tissue collection is important, and have therefore included this information in the result section:

Page 11: "[…] To evaluate whether VA could function as an LRH-1 agonist *in vivo*, we administered a mixture of *cis*- and *trans*-VA (50 mg/kg) or its vehicle via intraperitoneal (i.p.) injection into male C57Bl/6 mice (**Fig 4A**). **Mice had *ad libitum* access to food and water. Tissue was collected either 8 h or 18 h post-injection, as indicated in the figure legend."**

If available, reporting hepatic SHP and intestinal Fgf15 expression would further help determine whether FXR signaling contributes to the observed regulation of bile acid synthesis genes.

Answer: We thank the reviewer for pointing this out. As suggested, we have measured expression of *Shp* and *Fgf15* in hepatic and small intestinal tissue, respectively, of male C57BL/6 mice 18 h after i.p. injection of VA or its vehicle (same experimental conditions as used for Fig 4A and described in the methods section). We observed upregulation of both *Shp* and *Fgf15* after VA treatment. As pointed out by the reviewer, this suggests that FXR signaling contributes to the observed effects of VA, including downregulation of *Cyp7a1* and *Cyp8b1*. This reveals the existence of an intriguing, complex cross-talk with the FXR-SHP-FGF15 axis.
We have added these gene expression data in the revised manuscript in panel B and C of Fig. EV5:

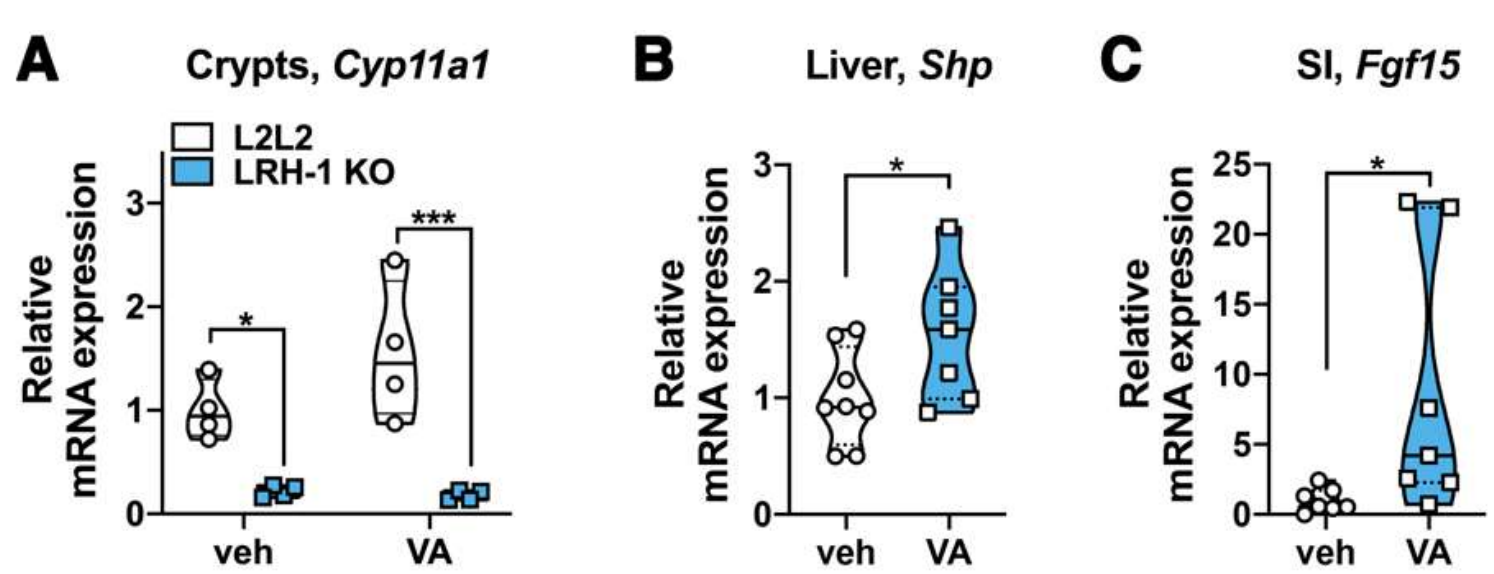

Figure EV5 *(related to Fig 4): VA-induced expression of steroidogenic enzymes in primary intestinal crypt cells requires the presence of LRH-1; investigation on FXR-SHP-FGF15 axis contribution to VA-induced effects.*

(A) *Primary crypt cells from small intestine (SI) of control LRH-1 L2L2 and LRH-1 VilCre mice with LRH-1 deletion in intestinal epithelial cells (LRH-1 KO) were isolated and cultured for 4 h ex vivo with 240 µM mixture of cis- and trans-VA or its vehicle control (veh). N= 4, two-way ANOVA with Tukey´s correction, *: P < 0.05; ***: P < 0.001. Isolation and culture of intestinal crypts was done as described in Noti et al. 2010.*
(B, C) *Increased expression of hepatic Shp (B) and intestinal Fgf15 (C) in mice treated for 18 h with VA compared to its vehicle control suggesting a complex crosstalk between VA-induced LRH-1 activation and the FXR-SHP-FGF15 axis. n=7-8, each dot represents an individual mouse.*

We have also included a sentence in the result section:

Page 12: **Interestingly, hepatic *Shp* and intestinal *Fgf15* were upregulated after VA treatment (Fig EV5B, C), suggesting involvement of FXR signaling and the existence of a complex cross-talk between LRH-1 and the FXR-SHP-FGF15 axis.**

- The authors show that bacterial lipid extracts fail to activate the LRH-1 LBP mutant (F342W/I416W) (Fig 1D), supporting ligand-dependent activation. However, it is not reported whether VA was tested on this mutant receptor. Since VA was evaluated in the LRH-1 reporter assay (Fig 2F), it would be important to confirm in parallel that VA does not activate the F342W/I416W mutant, which was already used to validate bacterial extract-mediated activation. Demonstrating reduced or absent responsiveness of the mutant to pure VA would further strengthen the conclusion that VA directly engages the ligand-binding pocket of LRH-1.

Answer: The reviewer is correct, a reduced responsiveness of the LRH-1 mutant F342W/I416W to VA stimulation would strengthen the conclusion that VA directly interacts with the LRH-1 ligand-binding pocket. We have thus analyzed the activation of the LRH-1 mutant F342W/I416W by VA, and have indeed found that the LRH-1 ligand-binding mutant shows a largely reduced responsiveness to both *cis*- and *trans*-VA. We have added these new data as panel D and E in Fig. EV3, as well as this sentence in the results section (page 9):
"Basal activity and responsiveness to both *cis*- and *trans*-VA was reduced in mutant LRH-1 F342W/I416W (Fig EV3D, E) compared to wild type LRH-1 (Fig EV3B, C), validating the hypothesis that VA directly engages the LBP of LRH-1."

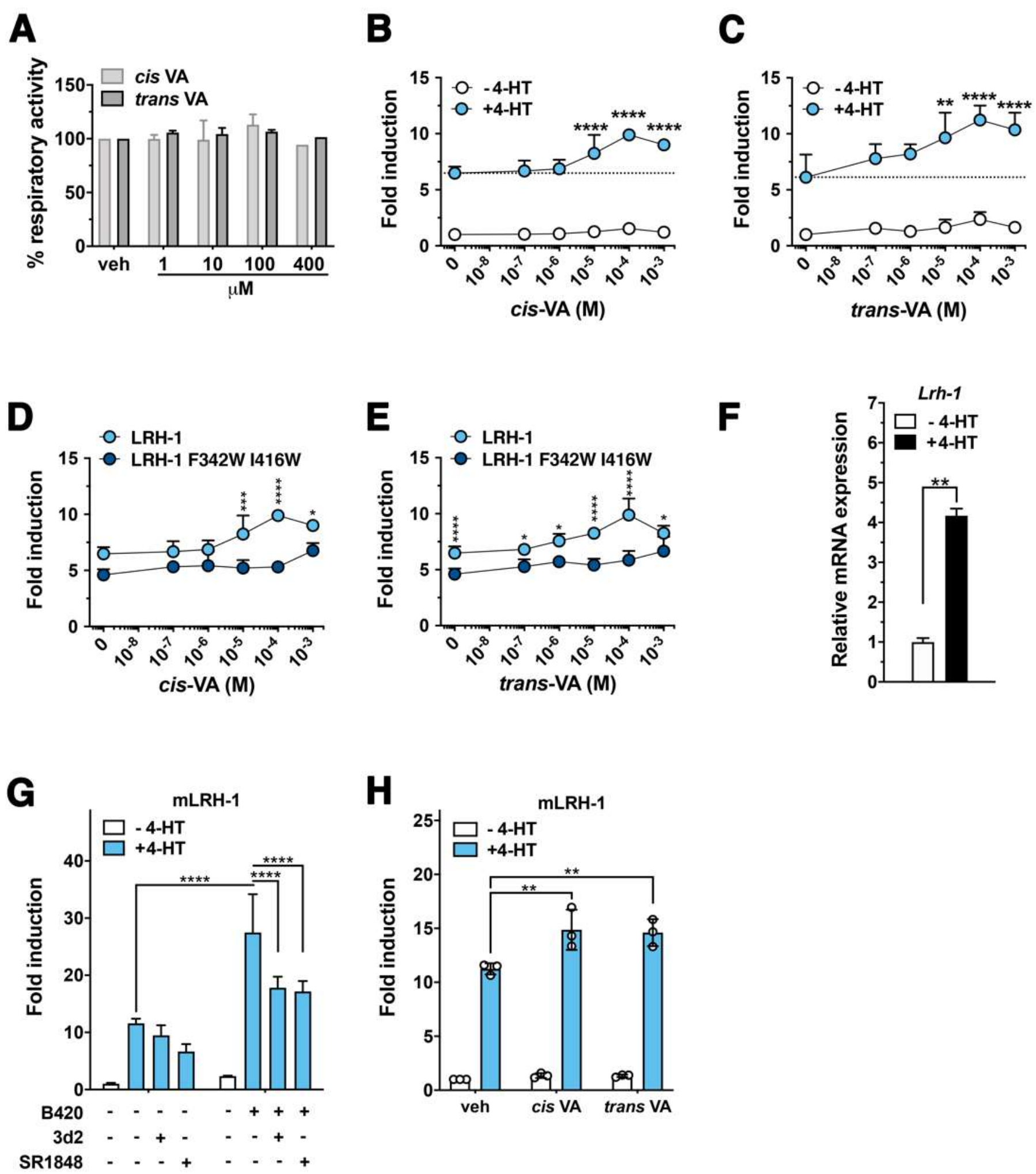

Figure EV3 (related to Fig 2): Characterization of cis- and trans-VA-mediated LRH-1 transactivation; verification of murine LRH-1 transactivation by B420 and VA

Experiments were performed in LS174T cells with 4-HT-inducible expression of wild type or mutant human Flagged-tag LRH-1, or murine LRH-1 (4 h, unless differently indicated).

(A) *Cell viability (MTT assay) in LRH-1 cells treated with the indicated concentrations of cis- or trans-VA. Mean ± SD of two independent experiments performed in duplicates.*

(B, C) *Effects of cis- (B) or trans-VA (C) on the transcriptional activity of LRH-1 in cells with uninduced (- 4-HT) or induced expression of LRH-1 (+ 4-HT). Mean values of technical triplicates ± SD of one representative experiment (n=3) are shown. Two-way ANOVA, **: P-value < 0.01; ****: P-value < 0.0001.*

(D, E) *Effects of cis- (D) or trans-VA (E) on the transcriptional activity of wild type or F342W I416W mutant LRH-1. Mean values of technical triplicates ± SD of one experiment are shown. Two-way ANOVA comparing LRH-1 vs LRH-1 F342W I416W for each concentration, *: P-value < 0.05; **: P-value < 0.01; ***: P-value < 0.001; ****: P-value < 0.0001.*

(F) *Quantification of murine Lrh-1 mRNA by RT-qPCR in mLRH-1 with 200 nM 4-HT for 6 h. Mean ± SD of one experiment performed in duplicates.*

(G, H) *LRH-1 transcriptional activity in cells with 4-HT-inducible expression of mLRH-1. Cells were incubated with 200 µg/ml B420 extract (G), with 100 µM cis-VA or trans-VA (H) or the vehicle (ethanol, veh) for 16 h. For inhibition of LRH-1 activity, cells were treated with 3d2 or SR1848. Mean ± SD of one experiment performed in triplicates (G) or of 3 independent experiments in triplicates (H). Two-way ANOVA, **: P-value < 0.01; ****: P-value < 0.0001.*

- The authors report that cis-VA is detected in multiple bacterial species, whereas trans-VA appears to be specific to B420, and that the bioactive fraction contained both isomers. However, the in vivo experiments

were performed using a 1:1 mixture of cis- and trans-VA. Given that cis-VA displays a markedly higher binding affinity to LRH-1 compared to trans-VA, the relative proportion of the two isomers may influence the overall biological effect. It would therefore be helpful to clarify the rationale for selecting a 1:1 mixture and whether this reflects the relative abundance of the isomers in the active bacterial extract. Providing this information would improve transparency and strengthen the physiological interpretation of the in vivo data.

Answer: We thank the reviewer for this insightful comment. The main reason why we chose to use a 1:1 mixture of both *cis*- and *trans*-VA for the animal experiments is that both VA isomers showed to be similarly active in our cell-based functional assay, despite the different binding constants (Kd) assessed *in vitro* by microscale thermophoresis. This is likely due to the influence of the more physiological cellular environment, compared to direct binding of *cis*- and *trans*-VA to the LRH-1 ligand binding domain *in vitro*, where the functional outcome is likely shaped by membrane partitioning, signal amplification via interaction with cofactors or partners, intracellular metabolic conversion to effectors shared with other NRs, potential saturation of the functional response or differences in solubility in the different buffers. In addition, *cis-trans* isomerization in mice, for example by the resident microbiota, cannot be excluded.
Encouraged by the reviewer's suggestion we have now analysed the relative levels of *cis*- and *trans*-VA in B420 extracts, and detected comparable ratios.

- The Discussion mentions that physiological concentrations of VA in humans are in the micromolar range. It would be helpful to briefly relate this information to the dosing used in the present in vivo experiments (50 mg/kg i.p.), and to discuss whether comparable micromolar concentrations are expected to be achieved in plasma or liver under these conditions.

Answer: We thank the reviewer for raising this important point. In the present study, mice received an intraperitoneal administration of 50 mg/kg, which corresponds to approximately 1.75 mg per animal with approx. 35 g body weight (HFD), and to approx. 6 µmol VA per mouse. Assuming a distribution within a blood volume of ~2.4 mL, a theoretical peak plasma concentration in the range of 2–3 mM could be estimated. However, circulating fatty acids are subject to rapid clearance and redistribution. A study showed that injected fatty acids exhibit clearance from plasma and uptake by tissues, including the liver, within less than one minute (Li *et al*, 2022). Consequently, plasma concentrations are expected to fall rapidly into a similar micromolar range to that reported to total plasma free FA, approx. 100-200 µM (Tomoo *et al*, 2024). After absorption, long-chain FA are efficiently taken up by the liver and peripheral tissues. However, intracellular FA are extensively bound to proteins, incorporated into membranes, or esterified into lipid pools, such that total tissue levels do not directly reflect the free concentration of VA available for receptor activation, which would likely fall into the low micromolar range.

We hope that these calculations/estimations helped to clarify this issue and support the suggestion that biologically active VA concentrations can be achieved *in vivo*.

- The number of animals included in each experimental group should be clearly indicated in the figure legends or Methods section. Providing the exact n per group is essential to assess statistical power and interpret the robustness of the reported metabolic and histological outcomes.

Answer: We apologize that we forgot to indicate the number of animals for a few figures. We have added the missing information in the relevant figure legends: Fig 5 (B to H) and Fig 6D.

Referee #2 (Remarks for Author):

This elegant study, led by the Brunner group in collaboration with several international teams, reports the identification of vaccenic acid, a metabolite derived from intestinal bacteria, as a novel LRH-1/NR5A2 agonist implicated in the regulation of hepatic metabolism. The authors employ a broad and complementary range of approaches, spanning in vitro metabolite screening and mechanistic characterization to in vivo metabolic analyses in multiple mouse models. The study is carefully designed and rigorously executed, with potential caveats thoughtfully addressed and the conclusions supported by a strong and coherent experimental framework. I only have a few comments:

Answer: We appreciate the reviewer's positive feedback as well as the valuable points raised, which we addressed and incorporated in the revised manuscript. Our detailed, point-by-point responses are provided below.

In Figure EV1 and 1, please provide rationale of using the epithelial-like LS174T cell line established from the colon of a colorectal adenocarcinoma rather than a hepatic-derived cell line.

Answer: The main reason why we used intestinal epithelial-like LS174T cells is that this tool was already developed and characterize in the lab. In the past our LRH-1-based research had been strongly focused on the role of LRH-1 in regulating glucocorticoid synthesis in intestinal epithelial cells, and we had developed this cell line as a screening tool to measure LRH-1 activity. At the outset of this project, we pursued both, intestinal glucocorticoid synthesis as well as hepatic metabolism, but due to published data on DLPC-induced LRH-1 activation *in vivo* and its effects on liver metabolism we decided to primarily focus on VA-induced and LRH-1 regulated liver metabolism. Equally important is the fact that liver cells have very high basal LRH-1 expression, which makes them less suitable for screening applications, whereas LS174T cells have very low basal LRH-1 expression levels, allowing to inducibly express wild type and mutant LRH-1.

We have added this clarification before Fig 1 and EV1:
Page 6: **This colorectal epithelial cell line, unlike hepatic cell lines, has the advantage of low endogenous LRH-1 expression, making it well suited for investigating the effects of induced LRH-1 overexpression.**

In Figure EV2, please clarify why Caco-2 cells rather than the LS174T cells with the inducible LRH-1 construct.

Answer: Caco-2 cells, differently from LS174T cells, have high endogenous LRH-1 expression. We preferred to use these cells for the identification of bioactive compounds in the bacterial extracts to reduce the risk of potential artifacts due to massive overexpression of the Flag-tagged LRH-1 with the inducible system.
To clarify this point, we have replaced the sentence:

Page 8: "These were evaporated, resuspended in ethanol and used to treat Caco-2 cells transfected with the 5xRE LRH-1 luciferase reporter (**Fig EV2B**)."

with the following one: "**These were evaporated, resuspended in ethanol and used to screen for LRH-1 transactivation in Caco-2 cells transfected with the 5xRE LRH-1 luciferase reporter, which express high levels of endogenous LRH-1 compared to LS174T cells,** (**Fig EV2B**)."

In Figure 3D and E the authors shown that the Kd of trans- VA is 10 X higher that for cis-VA (144.4 uM 14.2) yet in Figure 2F both cis and trans-VA display the same capacity for activating LRH-1. Could they provide an explanation as one would expect less activation potential for trans-VA.

Answer: We thank the reviewer for pointing this out, and we agree that clarification of this point is important. While the microscale thermophoresis measures direct interactions of cis- and trans-VA with the ligand binding domain of LRH-1 in vitro, the situation within a cell is already far more complex and the transcriptional activity of LRH-1 is not only affected by the binding affinity of different LRH-1 ligands, but by various other aspects, such as metabolism, co-regulators, bioavailability, etc. To address this aspect, we have added the following sentence to the discussion section:

Page 18: "**Interestingly, although a 10-fold higher K_d was estimated for *trans*-VA compared to *cis*-VA, both displayed similar LRH-1-activating capacity in cell-based functional studies. In the cell, multiple determinants of the final functional output, including signal amplification via interaction with cofactors or partners, metabolic conversion to effectors shared with other NRs, potential nonlinear or saturated functional response or improved bioavailability may account for these differences.**"

In vivo administration of VA to HFD as well as to SD fed mice resulted in decreased glucose serum levels (Figure 5D). Yet insulin levels were only decreased in the HFD group (Figure 5E) suggesting that lowering of glycemia was not due to improved glucose tolerance by peripheral tissues but rather improved liver function (which would make sense), albeit the RNAseq did not reveal any genes involved in glucose metabolism (Figure 6A). Did the authors perform any oral glucose (or insulin) tolerance test to rule out the role of other peripheral tissues such as muscle?

Answer: We thank the reviewer for raising this interesting point. We had not done glucose or insulin tolerance experiments during the course of our study, as the primary focus was the identification of bacteria-derived

LRH-1 ligands and their effects in an LRH-1-regulated mouse model. At this stage, unfortunately, neither our lab nor coauthors have an approved ongoing animal protocol to perform such animal experiments and address these certainly interesting research questions. Filing a new animal experimentation protocol and its approval by the government authorities can currently take easily up to 9 months. Thus, to conduct these experiments would have been outside of the time frame for these revisions.
We believe, however, that in the current literature there is already sufficient evidence that VA, which we here describe for the first time as an LRH-1 ligand, has beneficial effects on blood glucose levels. Thus, these published data, investigated in a completely different context, match very well with our own observations and support our overall findings and message. The decrease in serum insulin levels only in the HFD group suggests that lower glycemia is not due to improved insulin sensitivity in peripheral tissues after VA administration, but rather to improved liver function. Indeed, VA was already shown to reduce insulin and glucose responses after a meal tolerance test (MTT) in obese JCR:LA-cp rats (Jacome-Sosa 2014). Similarly, trans-VA-enriched beef or milk fats alleviated insulin resistance and glucose intolerance in obese JCR:LA-cp rats (Diane et al 2006 Lipids 51, 821-831) and high-fat-fed Wistar rats (Sain et al 2023 Eur J Nutr 62, 1535), respectively. Yet, another study found that trans-VA in beef fat did not improve glucose homeostasis and hepatic lipid accumulation in HFD-induced obese mice (Xu 2024 British J of Nutr. 131).

We have included discussion of this point based on the available literature in the manuscript:
Page 18: “Interestingly, VA-induced health benefits correlate with those already shown for the LRH-1 ligands DLPC and 10CA (Lee et al, 2011; Mays et al, 2022). Likewise, dietary supplementation of VA in animal models was shown to improve hepatic steatosis, and decrease serum glucose and insulin (Jacome-Sosa *et al*, 2014). **The authors suggested that VA can improve whole-body insulin resistance, although another study reported that long-term feeding of beef fat enriched in trans-VA did not improve glucose homeostasis (Xu et al, 2024).”**

Figure 6A and its description in the Results section are unclear. The dot plot displays Gene Ratio values, which represent enrichment magnitude but do not convey directionality. The term "VA-induced" implies pathway activation; however, it is not specified whether these pathways are enriched among upregulated genes, downregulated genes, or the full gene set. The authors should clarify the gene list used for enrichment and revise the terminology accordingly.

Answer: We thank the reviewer for this important comment. We agree that GO enrichment based on Gene Ratio does not convey directionality. In our analysis, the GO enrichment shown in Figure 6A was performed on the combined set of significantly differentially expressed genes (both up- and downregulated; $log_2FC > 1 / -1$, $p < 0.05$). Therefore, the identified biological processes reflect pathways that are modulated by VA treatment, rather than exclusively activated or repressed.
We have revised the wording in the results section and figure legend to clarify this point and avoid implying directionality. Importantly, although the enrichment analysis does not distinguish activation from repression, the affected pathways, particularly lipid and fatty acid metabolism, are consistent with the observed transcriptional changes in key metabolic regulators and the phenotypic effects on hepatic steatosis *in vivo*. Together, these findings support a biologically meaningful remodeling of hepatic lipid metabolism in response to VA treatment.
We have made the following changes in the text:

Fig 6A: we have changed the title from “VA-induced Biological Processes” to “**VA-modulated Biological Processes**”.

Results section, page 14: we have changed “Gene ontology analysis revealed that VA treatment predominantly affected lipid biosynthetic processes, pathways related to lipid and fatty acids metabolism (**Fig 6A**), and modulated several well-known LRH-1 target genes (**Fig EV8**).”
to: “**Gene ontology analysis of significantly differentially expressed genes revealed that VA treatment modulates pathways related to lipid biosynthesis and fatty acid metabolism (Fig 6A), alongside altered expression of LRH-1 target genes (Fig EV8).”**

Page 37, Figure legend Fig 6:
from: **(A, B)** Top 20 gene ontologies (A) and heatmap of the top 50 differentially expressed genes (B) induced by VA treatment analyzed with bulk RNA sequencing of liver samples from HFD mice treated with either vehicle control (veh, n=5) or VA (n=5).
Modified to: **(A, B) Top 20 enriched gene ontologies (GO) (A) and heatmap of the top 50 differentially expressed genes (DEGs, both up- and downregulated, B) identified from bulk RNA sequencing of liver**

samples from HFD mice treated with either vehicle control (veh, n=5) or VA (n=5). GO enrichment was performed using set of significantly up- and downregulated genes, reflecting biological processes modulated by VA treatment.

Referee #3 (Remarks for Author):

In this manuscript, Plazzo et al present new evidence for a microbially produced modulator of LRH-1 activity with impact on hepatic steatosis and glucose homeostasis. This work brings new insight into LRH-1 ligand binding while linking receptor activity with host-microbiome communication. The findings are impactful in that they 1) establish fatty acids (FA) as a novel ligand for this orphan receptor, 2) provide a new mechanism that links a microbially-produced FA to host metabolism, 3) supports FA ligand binding by the murine LRH-1 isoform, and 4) adds to the growing literature supporting the therapeutic targeting of LRH-1 for metabolic disorders including MASLD.

Strengths of this work include the breadth of study from biochemical analysis of FA binding to the LRH-1 LBD through in vitro and in vivo studies using mouse and human receptor isoforms. In particular, the use of the LRH-1 LBD double mutant supports the VA binding within LRH-1's binding pocket. Specificity is further supported through the use of inducible LRH-1 expression cell models, analysis of non-VA microbially produced FAs, and use of MST to demonstrate physical association of VA with the LRH-1 LBD. Together, this work supports the conclusion that VA agonizes LRH-1 in cellular and animal contexts with physiological consequences.

Answer: We thank the reviewer for the positive feedback and suggestions that we have incorporated at our best into the revised manuscript.

Minor suggestions/concerns for the study are as follows:

LRH-1 biochemical specificity. In the MST assay, data are provided for VA and a panel of FA associating with LRH-1 LBD. Parallel experiments with the mutant LRH-1 LBD would provide further support for specific LRH-1-VA binding in the LBD binding pocket.

Answer: We are grateful to the reviewer for this important suggestion. Indeed, parallel MST experiments with the mutant LRH-1 LBD F342W/I418W would have provided further support for specific binding of VA into the LBP. Unfortunately, we are not able to express and purify the mutant LRH-1 LBD, since the coauthor responsible for the expression and purification of the LRH-1 LBD had left the University of Konstanz and academia, and the optimization of the whole procedure would not have been possible in the time frame of the revision. However, to address this important point we have performed experiments in our cell line with inducible expression of the LBD mutant LRH-1 to characterize the functional effects of VA. We believe these data are relevant to support our cellular and *in vivo* experiments.

Hepatic LRH-1 engagement in vivo. The authors show improvements in hepatic steatosis, blood glucose, and insulin in HFD model with C57/Bl6 mice. The glucose measurements, however, are provided at a single time point. Glucose and insulin tolerance tests could be considered to better define the impact of VA treatment on glucose homeostasis.

Answer: We agree with this reviewer that assessing the effect of VA in glucose and insulin tolerance test would have been very interesting and informative. However, as already discussed in our response to the comments of reviewer 2, these analyses would not have been possible within the time frame for the revisions of this manuscript. Currently we do not have an approved animal protocol to perform these glucose or insulin tests. Filling a new animal experimentation protocol and approval by the government authorities can currently easily take up to 9 months. There is, however, sufficient information available in the current literature that support beneficial effects of VA in glucose and insulin tolerance tests (e.g. Diane et al 2006 Lipids 51, 821-831; Sain eta l 2023 Eur J Nutr 62, 1535).

Additionally, use of the group's Lrh1-flox mice with AAV-delivered Cre (as done in Miranda et al JCI Insights 2018) would further validate in vivo hepatic LRH-1 engagement.

Answer: This is certainly an interesting approach to further substantiate the specific role of LRH-1 in the liver in the regulation of VA-induced effects *in vivo*. Unfortunately, these experiments would suffer from the same

logistic problems as discussed above, and would not only involve the government authorities approving new animal experimentations, but also the government authorities approving work with recombinant viruses. Furthermore, this approach represents a technology, which is not yet established in our lab and would thus take considerable time until it would produce meaningful results. We thus hope that the reviewer understands that we have been unbale to pursue this experimental suggestion in the time remaining for the revisions.

Hepatic Steatosis. Liver histology provided in Fig. 5 is reminiscent of Miranda et al JCI Insights 2018 findings describing LRH-1-mediated lipid droplet remodeling related to Elovl5 and Fads2 expression. These genes are labeled in EV8 but the relationship to the current study should be discussed in the main text.

Answer: We thank the reviewer for this insightful comment. We have now added a sentence in the discussion (page 19) referring to this publication and the relationship with our own data reported in this study.

LRH-1 Target Gene Expression. Gene targets for LRH-1 are assessed and found to variable, which may relate to how specifically VA activates LRH-1 or impacts co-factor recruitment. The clear decrease in Cyp7a1 and Cyp8b1, however, raises the possibility that LRH-1 agonism broadly impacts bile acids that feed-back on these Cyp genes. Have the authors measured total bile acids?

Answer: We thank the reviewer for raising this interesting point. As discussed above in our response to reviewer 1, there seems to be indeed an interesting link between VA-induced LRH-1 activation and bile acid synthesis, which may be possibly related to a crosstalk between LRH-1 and FXR. In the course of this study, we have not been able to measure bile acids, as the volume of plasma obtained in our experimental models has been limited, and our primary focus has been the analysis of plasma glucose, insulin, triglycerides, cholesterol and liver transaminases. While the effects of VA on bile acid synthesis are interesting, we feel that at this stage this research question is outside the scope of our study.

3rd Jul 2026

Dear Prof. Brunner,

Thank you for the submission of your revised manuscript to EMBO Molecular Medicine. I am pleased to inform you that we will be able to accept your manuscript pending the following final amendments:

1) Authors: Please indicate the corresponding author on the title page and provide contact information.
2) Please consider revising the title to better highlight the main findings, e.g. "Microbiota-derived vaccenic acid activates LRH-1/NR5A2 and ameliorates metabolic liver disease"
3) Abstract: I have gone through your text and revised it (see below). Please review it and amend as you see fit:

Microbiota-derived metabolites influence host physiology and contribute to the development of metabolic diseases, yet the molecular mechanisms underlying microbiome-host communication remain incompletely understood. Here, we identify bacterial lipids as modulators of the nuclear receptor Liver Receptor Homolog-1 (LRH-1/NR5A2), a regulator of hepatic metabolism. Lipid extracts from multiple bacterial species, including the probiotic Bifidobacterium animalis subsp. lactis B420{trade mark, serif}, activated LRH-1, leading to the identification of the long-chain fatty acid vaccenic acid (VA) as a previously unrecognized endogenous LRH-1 ligand. VA directly bound the LRH-1 ligand-binding domain and stimulated receptor-dependent transcriptional activity. In high-fat diet-induced obese and hyperglycemic mice, VA-mediated LRH-1 activation improved glucose homeostasis and attenuated metabolic liver disease. Transcriptomic analyses revealed suppression of hepatic de novo lipogenesis as a principal downstream response to LRH-1 activation. Together, these findings establish a microbiota-LRH-1 signaling axis that links bacterial lipid metabolism to host metabolic regulation, identify vaccenic acid as a functional LRH-1 agonist, and provide a mechanistic rationale for targeting LRH-1 in metabolic liver disease.

4) In the main manuscript file, please do the following:
- Please address all comments suggested by our data editors listed below:
o Figure legends:
1. Please note that the legend for figure 1 is not provided in the sequential manner. This needs to be rectified.
2. Please note that the legends for figures 3E-H is missing in the manuscript. This needs to be rectified.
3. Please define the annotated p values ****/***/**/* as well as provide the exact p-values for the same in the legend of figure 4C, D as appropriate.
4. Please note that the exact p values are not provided in the legends of figures 1A, B, D, E; 2F, 4B, 5A, B, D, E, G; 6D, EV1 A, C, D, E, F, G, H, I, J; EV3 B, C, D, E, F, G, H; EV4 E-G; EV5 A-C; EV6 A-C; EV7 E.
5. Please note that information related to n is missing in the legends of figures 6C, EV7 C-F, I, J.
6. Please indicate the statistical test used for data analysis in the legends of figures 4C, D; 6C.
- Rename Material and Methods to Methods.
- In Methods, provide the antibody dilutions that were used for each antibody.
- In Methods, add a dedicated graphics paragraph to the Methods as follows:
Graphics:
(some of the... OR Figure #... OR synopsis) Graphics were created with BioRender.com.
- Indicate in legends number and nature of replicates and exact p= values, not a range, along with the statistical test used. To keep the figures "clear" some authors found providing an Appendix table Sx with all exact p-values preferable. You are welcome to do this if you want to.
- In Disclosure and competing interests statement please state employment in a biotech company (affiliation #7).
- Please move "Acknowledgments" between "Data availability statement" and "Disclosure and competing interests statement".
- Please rename Table I and Table II to Table 1 and Table 2.
5) Funding: Make sure that information about all sources of funding are complete in both our submission system and in the manuscript. Currently the Brigitte Schlieben Lange-Program, Baden Würrttemberg is missing in our submission system. Please correct.
6) Appendix:
- Please move all suppl. methods to the main "Methods" section.
- Rename suppl. data to "Appendix Data" and cite it at the appropriate place(s) in the main manuscript text. Figure 1 etc. should be removed while description of the spectra should remain.
- Please add page number in the table of content on the title page.
7) The Paper Explained: Please add I tot the main manuscript file.
8) Synopsis:
- Synopsis image: Please resize the image exactly 550 px-wide x 300 pixels high and upload it as a high-resolution jpeg file.
- Please check your synopsis text and image before submission with your revised manuscript. Please be aware that in the proof stage minor corrections only are allowed (e.g., typos).
9) As part of the EMBO Publications transparent editorial process (see our Editorial at http://embomolmed.embopress.org/content/2/9/329), EMBO Molecular Medicine will publish online a Review Process File (RPF) to accompany accepted manuscripts. This file will be published in conjunction with your paper and will include the anonymous

referee reports, your point-by-point response and all pertinent correspondence relating to the manuscript. Let us know if you want to remove or not any figures from it prior to publication. Please note that the Authors checklist will be published at the end of the RPF.
10) Please provide a point-by-point letter INCLUDING my comments as well as the reviewer's reports and your detailed responses (as Word file).

I look forward to reading a new revised version of your manuscript as soon as possible.

Yours sincerely,

Zeljko Durdevic

Zeljko Durdevic
Senior Editor
EMBO Molecular Medicine

*** Instructions to submit your revised manuscript ***

*** PLEASE NOTE *** As part of the EMBO Publications transparent editorial process initiative (see our Editorial at https://www.embopress.org/doi/pdf/10.1002/emmm.201000094), EMBO Molecular Medicine will publish online a Review Process File to accompany accepted manuscripts.

In the event of acceptance, this file will be published in conjunction with your paper and will include the anonymous referee reports, your point-by-point response and all pertinent correspondence relating to the manuscript. If you do NOT want this file to be published, please inform the editorial office at contact@embomolmed.org.

*****

When preparing your revised manuscript, please refer to our guidelines: https://link.springer.com/journal/44321/submission-guidelines#cms-Revised-submissions. We perform an initial quality control of all revised manuscripts before re-review; failure to include requested items will delay the evaluation of your revision.

We require:

1) A .docx formatted version of the manuscript text (including legends for main figures, EV figures and tables). Please make sure that the changes are highlighted to be clearly visible.

2) Individual production quality figure files as .eps, .tif, .jpg (one file per figure). For guidance, download the 'Figure Guide PDF': https://media.springernature.com/original/springer-cms/rest/v1/content/27825798/data/v1.

3) A .docx formatted letter INCLUDING the reviewers' reports and your detailed point-by-point responses to their comments. As part of the EMBO Press transparent editorial process, the point-by-point response is part of the Review Process File (RPF), which will be published alongside your paper.

4) A complete author checklist, which you can download from our author guidelines. Please insert information in the checklist that is also reflected in the manuscript. The completed author checklist will also be part of the RPF.

5) Please note that all corresponding authors are required to supply an ORCID ID for their name upon submission of a revised manuscript.

6) It is mandatory to include a 'Data Availability' section after the Materials and Methods. Before submitting your revision, primary datasets produced in this study need to be deposited in an appropriate public database, and the accession numbers and database listed under 'Data Availability'. Please remember to provide a reviewer password if the datasets are not yet public.

In case you have no data that requires deposition in a public database, please state so in this section. Note that the Data Availability Section is restricted to new primary data that are part of this study.

7) For data quantification: please specify the name of the statistical test used to generate error bars and P values, the number (n) of independent experiments (specify technical or biological replicates) underlying each data point and the test used to calculate p-values in each figure legend. The figure legends should contain a basic description of n, P and the test applied. Graphs must include a description of the bars and the error bars (s.d., s.e.m.).

8) At EMBO Press we ask authors to provide source data for the main manuscript figures. You will receive a separate email with instructions for providing source data with your revised manuscript, including how to upload and organize the files.

9) Our journal encourages inclusion of *data citations in the reference list* to directly cite datasets that were re-used and obtained from public databases. Data citations in the article text are distinct from normal bibliographical citations and should directly link to the database records from which the data can be accessed. In the main text, data citations are formatted as follows: "Data ref: Smith et al, 2001" or "Data ref: NCBI Sequence Read Archive PRJNA342805, 2017". In the Reference list, data citations must be labeled with "[DATASET]". A data reference must provide the database name, accession number/identifiers and a resolvable link to the landing page from which the data can be accessed at the end of the reference.

10) We replaced Supplementary Information with Expanded View (EV) Figures and Tables that are collapsible/expandable online. A maximum of 5 EV Figures can be typeset. EV Figures should be cited as 'Figure EV1, Figure EV2" etc... in the text and their respective legends should be included in the main text after the legends of regular figures.

- For the figures that you do NOT wish to display as Expanded View figures, they should be bundled together with their legends in a single PDF file called *Appendix*, which should start with a short Table of Content. Appendix figures should be referred to in the main text as: "Appendix Figure S1, Appendix Figure S2" etc.

- Additional Tables/Datasets should be labeled and referred to as Table EV1, Dataset EV1, etc. Legends have to be provided in a separate tab in case of .xls files. Alternatively, the legend can be supplied as a separate text file (README) and zipped together with the Table/Dataset file.

11) The paper explained: EMBO Molecular Medicine articles are accompanied by a summary of the articles to emphasize the major findings in the paper and their medical implications for the non-specialist reader. Please provide a draft summary of your article highlighting

- the medical issue you are addressing,

- the results obtained and

- their clinical impact.

This may be edited to ensure that readers understand the significance and context of the research. Please refer to any of our published articles for an example.

12) Author contributions: You will be asked to provide CRediT (Contributor Role Taxonomy) terms in the submission system. These replace a narrative author contribution section in the manuscript.

13) A Conflict of Interest statement should be provided in the main text.

14) Every published paper includes a 'Synopsis' to further enhance discoverability. Synopses are displayed on the journal webpage and are freely accessible to all readers. They include a short stand first (maximum of 300 characters, including space) as well as 2-5 one-sentences bullet points that summarizes the paper. Please write the bullet points to summarize the key NEW findings. They should be designed to be complementary to the abstract - i.e. not repeat the same text. We encourage inclusion of key acronyms and quantitative information (maximum of 30 words / bullet point). Please use the passive voice. Please attach these in a separate file or send them by email, we will incorporate them accordingly.

Please also suggest a striking image or visual abstract to illustrate your article as a PNG file 550 px wide x 300 px high.

15) Include a Reagents and Tools Table as part of the Methods section, which can be downloaded from our author guidelines.

EMBO Molecular Medicine has a "scooping protection" policy, whereby similar findings that are published by others during review or revision are not a criterion for rejection. Should you decide to submit a revised version, I do ask that you get in touch after three months if you have not completed it, to update us on the status.

*Additional important information regarding Figures

Each figure should be given in a separate file and should have the following resolution:
Graphs 800-1,200 DPI
Photos 400-800 DPI
Colour (only CMYK) 300-400 DPI"

Figures are not edited by the production team. All lettering should be the same size and style; figure panels should be indicated by capital letters (A, B, C etc). Gridlines are not allowed except for log plots. Figures should be numbered in the order of their appearance in the text with Arabic numerals. Each Figure must have a separate legend and a caption is needed for each panel.

*Additional important information regarding figures and illustrations can be found at https://media.springernature.com/original/springer-cms/rest/v1/content/27825798/data/v1

***** Reviewer's comments *****

Referee #2 (Remarks for Author):

The authors have addressed, to the best of their ability and within ethical and practical constraints, the concerns raised by both Reviewers 1 and 2. Many of these points were addressed through additional experiments that not only reinforce but also complement the main conclusions of the study. I would like to congratulate the authors on this elegant and thoroughly revised manuscript, which is now significantly strengthened.

Referee #3 (Remarks for Author):

I am satisfied by the author's responses and additional data supplied in this resubmission and have no further concerns.

The authors addressed the remaining editorial issues.

20th Aug 2026

Dear Prof. Brunner,

We are pleased to inform you that your manuscript is accepted for publication and is now being sent to our publisher to be included in the next available issue of EMBO Molecular Medicine.

Your manuscript will be processed for publication by EMBO Press. It will be copy edited and you will receive page proofs prior to publication. Please note that you will be contacted by Springer Nature Author Services to complete licensing and payment information.

You may qualify for financial assistance for your publication charges - either via a Springer Nature fully open access agreement or an EMBO initiative. Check your eligibility: https://link.springer.com/journal/44321/how-to-publish-with-us

Should you be planning a Press Release on your article, please notify the production team via this form in order to coordinate publication and release dates: https://forms.cloud.microsoft/e/eH4A8JVP6g

If you have any questions, please do not hesitate to contact the Editorial Office. Thank you for your contribution to EMBO Molecular Medicine.

Yours sincerely,
Zeljko Durdevic

Zeljko Durdevic
Senior Editor
EMBO Molecular Medicine

>>> Please note that it is EMBO Molecular Medicine policy for the transcript of the editorial process (containing referee reports and your response letter) to be published as an online supplement to each paper. If you do NOT want this, you will need to inform the Editorial Office via email immediately. More information is available here: https://link.springer.com/partners/embo-press/editorial-policies#Peer%20review

EMBO Press Author Checklist

Corresponding Author Name: Thomas Brunner
Journal Submitted to: EMBO Molecular Medicine
Manuscript Number: EMM-2026-23418

USEFUL LINKS FOR COMPLETING THIS FORM
The EMBO Journal - Author Guidelines
EMBO Reports - Author Guidelines
Molecular Systems Biology - Author Guidelines
EMBO Molecular Medicine - Author Guidelines

Reporting Checklist for Life Science Articles (updated January
This checklist is adapted from Materials Design Analysis Reporting (MDAR) Checklist for Authors. MDAR establishes a minimum set of requirements in transparent reporting in the life sciences (see Statement of Task: 10.31222/osf.io/9sm4x). Please follow the journal's guidelines in preparing your
Please note that a copy of this checklist will be published alongside your article.

Abridged guidelines for figures
1. Data
The data shown in figures should satisfy the following conditions:
- the data were obtained and processed according to the field's best practice and are presented to reflect the results of the experiments in an accurate and unbiased manner.
- ideally, figure panels should include only measurements that are directly comparable to each other and obtained with the same assay.
- plots include clearly labeled error bars for independent experiments and sample sizes. Unless justified, error bars should not be shown for technical
- if n<5, the individual data points from each experiment should be plotted. Any statistical test employed should be justified.
- Source Data should be included to report the data underlying figures according to the guidelines set out in the authorship guidelines on Data

2. Captions
Each figure caption should contain the following information, for each panel where they are relevant:
- a specification of the experimental system investigated (eg cell line, species name).
- the assay(s) and method(s) used to carry out the reported observations and measurements.
- an explicit mention of the biological and chemical entity(ies) that are being measured.
- an explicit mention of the biological and chemical entity(ies) that are altered/varied/perturbed in a controlled manner.
- the exact sample size (n) for each experimental group/condition, given as a number, not a range;
- a description of the sample collection allowing the reader to understand whether the samples represent technical or biological replicates (including how many animals, litters, cultures, etc.).
- a statement of how many times the experiment shown was independently replicated in the laboratory.
- definitions of statistical methods and measures:
  - common tests, such as t-test (please specify whether paired vs. unpaired), simple $\chi2$ tests, Wilcoxon and Mann-Whitney tests, can be unambiguously identified by name only, but more complex techniques should be described in the methods section;
  - are tests one-sided or two-sided?
  - are there adjustments for multiple comparisons?
  - exact statistical test results, e.g., P values = x but not P values < x;
  - definition of 'center values' as median or average;
  - definition of error bars as s.d. or s.e.m.

Please complete ALL of the questions below.
Select "Not Applicable" only when the requested information is not relevant for your study.

Materials

Newly Created Materials	Information included in the manuscript?	In which section is the information available? (Reagents and Tools Table, Materials and Methods, Figures, Data Availability Section)
New materials and reagents need to be available; do any restrictions apply?	Yes	Materials and Methods

Antibodies	Information included in the manuscript?	In which section is the information available? (Reagents and Tools Table, Materials and Methods, Figures, Data Availability Section)
For **antibodies** provide the following information:<br>- Commercial antibodies: RRID (if possible) or supplier name, catalogue number and or/clone number<br>- Non-commercial: RRID or citation	Yes	Reagents and Tools Table

DNA and RNA sequences	Information included in the manuscript?	In which section is the information available? (Reagents and Tools Table, Materials and Methods, Figures, Data Availability Section)
**Short novel DNA or RNA including primers, probes**: provide the sequences.	Yes	Table I

Cell materials	Information included in the manuscript?	In which section is the information available? (Reagents and Tools Table, Materials and Methods, Figures, Data Availability Section)
**Cell lines:** Provide species information, strain. Provide accession number in repository **OR** supplier name, catalog number, clone number, and/**OR** RRID.	Yes	Reagents and Tools Table
**Primary cultures:** Provide species, strain, sex of origin, genetic modification status.	Not Applicable	
Report if the cell lines were recently **authenticated** (e.g., by STR profiling) and tested for mycoplasma contamination.	Yes	Materials and Methods

Experimental animals	Information included in the manuscript?	In which section is the information available? (Reagents and Tools Table, Materials and Methods, Figures, Data Availability Section)
**Laboratory animals or Model organisms:** Provide species, strain, sex, age, genetic modification status. Provide accession number in repository **OR** supplier name, catalog number, clone number, **OR** RRID.	Yes	Reagents and Tools Table
**Animal observed in or captured from the field:** Provide species, sex, and age where possible.	Not Applicable	
Please detail **housing and husbandry conditions**.	Yes	Materials and Methods

Plants and microbes	Information included in the manuscript?	In which section is the information available? (Reagents and Tools Table, Materials and Methods, Figures, Data Availability Section)
**Plants:** provide species and strain, ecotype and cultivar where relevant, unique accession number if available, and source (including location for collected wild specimens).	Not Applicable	
**Microbes:** provide species and strain, unique accession number if available, and source.	Yes	Reagents and Tools Table

Human research participants	Information included in the manuscript?	In which section is the information available? (Reagents and Tools Table, Materials and Methods, Figures, Data Availability Section)
If collected and within the bounds of privacy constraints report on age, sex and gender or ethnicity for all study participants.	Not Applicable	

Core facilities	Information included in the manuscript?	In which section is the information available? (Reagents and Tools Table, Materials and Methods, Figures, Data Availability Section)
If your work benefited from core facilities, was their service mentioned in the acknowledgments section?	Not Applicable	

Design

Study protocol	Information included in the manuscript?	In which section is the information available? (Reagents and Tools Table, Materials and Methods, Figures, Data Availability Section)
If study protocol has been **pre-registered, provide DOI in the manuscript**. For clinical trials, provide the trial registration number **OR** cite DOI.	Not Applicable	
Report the **clinical trial registration number** (at ClinicalTrials.gov or equivalent), where applicable.	Not Applicable	

Laboratory protocol	Information included in the manuscript?	In which section is the information available? (Reagents and Tools Table, Materials and Methods, Figures, Data Availability Section)
Provide DOI OR other citation details if **external detailed step-by-step protocols** are available.	Not Applicable	

Experimental study design and statistics	Information included in the manuscript?	In which section is the information available? (Reagents and Tools Table, Materials and Methods, Figures, Data Availability Section)
Include a statement about **sample size** estimate even if no statistical methods were used.	Yes	Figure legends
Were any steps taken to minimize the effects of subjective bias when allocating animals/samples to treatment (e.g. **randomization procedure**)? If yes, have they been described?	Yes	Materials and Methods
Include a statement about **blinding** even if no blinding was done.	Yes	Materials and Methods
Describe **inclusion/exclusion criteria** if samples or animals were excluded from the analysis. Were the criteria pre-established?<br><br>If sample or data points were omitted from analysis, report if this was due to attrition or intentional exclusion and provide justification.	Yes	Materials and Methods
For every figure, are **statistical tests** justified as appropriate? Do the data meet the assumptions of the tests (e.g., normal distribution)? Describe any methods used to assess it. Is there an estimate of variation within each group of data? Is the variance similar between the groups that are being statistically compared?	Yes	Figure legends

Sample definition and in-laboratory replication	Information included in the manuscript?	In which section is the information available? (Reagents and Tools Table, Materials and Methods, Figures, Data Availability Section)
In the figure legends: state number of times the experiment was **replicated** in laboratory.	Yes	
In the figure legends: define whether data describe **technical or biological replicates**.	Yes	

Ethics

Ethics	Information included in the manuscript?	In which section is the information available? (Reagents and Tools Table, Materials and Methods, Figures, Data Availability Section)
Studies involving **human participants**: State details of **authority granting ethics approval** (IRB or equivalent committee(s), provide reference number for approval.	Not Applicable	
Studies involving **human participants**: Include a statement confirming that **informed consent** was obtained from all subjects and that the experiments conformed to the principles set out in the WMA Declaration of Helsinki and the Department of Health and Human Services Belmont Report.	Not Applicable	
Studies involving **human participants: For publication of patient photos**, include a statement confirming that consent to publish was obtained.	Not Applicable	
Studies involving experimental **animals**: State details of **authority granting ethics approval** (IRB or equivalent committee(s), provide reference number for approval. Include a statement of compliance with ethical regulations.	Yes	Materials and Methods
Studies involving **specimen and field samples:** State if relevant **permits** obtained, provide details of authority approving study; if none were required, explain why.	Not Applicable	

Dual Use Research of Concern (DURC)	Information included in the manuscript?	In which section is the information available? (Reagents and Tools Table, Materials and Methods, Figures, Data Availability Section)
Could your study fall under dual use research restrictions? Please check biosecurity documents and list of **select agents and toxins** (CDC): https://www.selectagents.gov/sat/list.htm	Not Applicable	
If you used a select agent, is the security level of the lab appropriate and reported in the manuscript?	Not Applicable	
If a study is subject to dual use research of concern regulations, is the name of the **authority granting approval and reference number** for the regulatory approval provided in the manuscript?	Not Applicable	

Reporting

The MDAR framework recommends adoption of discipline-specific guidelines, established and endorsed through community initiatives. Journals have their own policy about requiring specific guidelines and recommendations to complement MDAR.

Adherence to community standards	Information included in the manuscript?	In which section is the information available? (Reagents and Tools Table, Materials and Methods, Figures, Data Availability Section)
State if relevant guidelines or checklists (e.g., **ICMJE, MIBBI, ARRIVE, PRISMA**) have been followed or provided.	Not Applicable	
For **tumor marker prognostic studies,** we recommend that you follow the **REMARK** reporting guidelines (see link list at top right). See author guidelines, under 'Reporting Guidelines'. Please confirm you have followed these guidelines.	Not Applicable	
For **phase II and III randomized controlled trials**, please refer to the **CONSORT** flow diagram (see link list at top right) and submit the CONSORT checklist (see link list at top right) with your submission. See author guidelines, under 'Reporting Guidelines'. Please confirm you have submitted this list.	Not Applicable	

Data Availability

Data availability	Information included in the manuscript?	In which section is the information available? (Reagents and Tools Table, Materials and Methods, Figures, Data Availability Section)
Have **primary datasets** been deposited according to the journal's guidelines (see 'Data Deposition' section) and the respective accession numbers provided in the Data Availability Section?	Yes	Data Availability Section
Were **human clinical and genomic datasets** deposited in a public access-controlled repository in accordance to ethical obligations to the patients and to the applicable consent agreement?	Not Applicable	
Are **computational models** that are central and integral to a study available without restrictions in a machine-readable form? Were the relevant accession numbers or links provided?	Not Applicable	
If publicly available data were reused, provide the respective **data citations in the reference list**.	Not Applicable	
