# Supplementary material for: Microbiome-host communication via vaccenic acid modulates LRH-1/NR5A2 activity and ameliorates metabolic liver disease
Source: EMBO Mol Med. 2026 Sep 17;18(10):4059–89. doi: 10.1038/s44321-026-00516-3 (PMC13658251; doi:10.1038/s44321-026-00516-3)
Supplement: Supplementary file 4 [file 44321_2026_516_MOESM1_ESM.docx]

**Table EV1**

| Gene | Reference (Author, Journal, Year) | Identifier (PMID, PMCID, DOI) |
| --- | --- | --- |
| Cyp7a1 | Goodwin et al., Mol Cell 2000 | PMID:11030332 |
| Cyp7a1 | Lu et al., Mol Cell 2000 | PMID:11030331 |
| Cyp8b1 | Lee et al., PNAS 2008 | PMCID:PMC2409274 |
| Abcg5 | Freeman et al., J Lipid Res 2004 | PMID:15121760 |
| Gck | Oosterveer et al., J Clin Invest 2012 | PMCID:PMC3408738 |
| Plin5 | Pantha et al., BMB Reports 2021 | PMCID:PMC8505233 |
| Asns | Sun et al., J Clin Invest 2023 | PMID: 36719750 |
| Il1rn | Venteclef et al., J Biol Chem 2007 | PMID:17158876 |
| Fads2 | Miranda et al., JCI Insight 2018 | DOI:10.1172/jci.insight.96151 |
| Elovl5 | Miranda et al., JCI Insight 2018 | DOI:10.1172/jci.insight.96151 |
| Cyp17a1 | Xiao et al., Cancer Res 2018 | DOI:10.1158/0008-5472.CAN-17-2341 |
| Cyp17a1 | Zhou et al., Oncogene 2021 | DOI:10.1038/s41388-021-01737-1 |
| Cyp17a1 | Camats et al., Sex Dev 2015 | DOI:10.1159/000381575 |
| Hmgcs1 | Chong et al., BMC Genomics 2012 | DOI:10.1186/1471-2164-13-51 |
| Ldlr | Chong et al., BMC Genomics 2012 | DOI:10.1186/1471-2164-13-51 |
| Ldlr | Miranda et al., JCI Insight 2018 | DOI:10.1172/jci.insight.96151 |

LRH-1 target genes, whose expression is affected by VA treatment in HFD mice, annotated in the Volcano plot of Fig EV8.
