# Supplementary material for: Microbiome-host communication via vaccenic acid modulates LRH-1/NR5A2 activity and ameliorates metabolic liver disease
Source: EMBO Mol Med. 2026 Sep 17;18(10):4059–89. doi: 10.1038/s44321-026-00516-3 (PMC13658251; doi:10.1038/s44321-026-00516-3)
Supplement: Supplementary file 6 [file 44321_2026_516_MOESM4_ESM.zip › Fig 2/2A, 2D, 2E/Fig 2A, 2D, 2E_chromatograms.docx]

**Raw Data**

**Fig 2A**: chromatographic separation of B420 extract

**Fig 2D**: Full view of chromatogram from standard *cis*- and *trans*-VA:

Full view of standard cis and trans VA with integrated peaks and peak table:

**Fig 2E**:

Full view of chromatogram from *E. faecalis* extract

Full view of chromatogram from *E. cloacae* extract

Full view of chromatogram from *P. aeruginosa* extract

Full view of chromatogram from *P. mirabilis* extract

Full view of chromatogram from *V. cholera* extract
