# Supplementary material for: Microbiome-host communication via vaccenic acid modulates LRH-1/NR5A2 activity and ameliorates metabolic liver disease
Source: EMBO Mol Med. 2026 Sep 17;18(10):4059–89. doi: 10.1038/s44321-026-00516-3 (PMC13658251; doi:10.1038/s44321-026-00516-3)
Supplement: Supplementary file 7 [file 44321_2026_516_MOESM5_ESM.zip › Fig 3/3A/Fig 3A_chromatogram.docx]

**Fig 3A**: full GC-MS chromatogram of B420 extract

Integrated *cis*- and *trans*-VA from extract with peak table:

Integrated *cis*- and *trans*-VA from extract with peak table:
